# Shared Neural Phenotypes for Mood and Anxiety Disorders: A Meta-analysis of 226 Task-Related Functional Imaging Studies

**DOI:** 10.1001/jamapsychiatry.2019.3351

**Published:** 2019-10-30

**Authors:** Delfina Janiri, Dominik A. Moser, Gaelle E. Doucet, Maxwell J. Luber, Alexander Rasgon, Won Hee Lee, James W. Murrough, Gabriele Sani, Simon B. Eickhoff, Sophia Frangou

**Affiliations:** 1 Icahn School of Medicine at Mount Sinai, New York, New York; 2 Faculty of Medicine and Psychology, Sapienza University of Rome, Rome, Italy; 3 School of Medicine and Psychology, Department of Neuroscience, Mental Health and Sensory Organs, Sapienza University, Sant'Andrea Hospital, Rome, Italy; 4 Centro Lucio Bini-Aretæus, Rome, Italy; 5 Institute of Neuroscience and Medicine (Brain and Behavior), Research Centre Jülich, Jülich, Germany; 6 Institute of Systems Neuroscience, Medical Faculty, Heinrich-Heine-University Düsseldorf, Düsseldorf, Germany

## Abstract

**Importance:**

Major depressive disorder, bipolar disorder, posttraumatic stress disorder, and anxiety disorders are highly comorbid and have shared clinical features. It is not yet known whether their clinical overlap is reflected at the neurobiological level.

**Objective:**

To detect transdiagnostic convergence in abnormalities in task-related brain activation.

**Data Source:**

Task-related functional magnetic resonance imaging articles published in PubMed, Web of Science, and Google Scholar during the last decade comparing control individuals with patients with mood, posttraumatic stress, and anxiety disorders were examined.

**Study Selection:**

Following Preferred Reporting Items for Systematic Reviews and Meta-analyses reporting guidelines, articles were selected if they reported stereotactic coordinates of whole-brain–based activation differences between adult patients and control individuals.

**Data Extraction and Synthesis:**

Coordinates of case-control differences coded by diagnosis and by cognitive domain based on the research domain criteria were analyzed using activation likelihood estimation.

**Main Outcomes and Measures:**

Identification of transdiagnostic clusters of aberrant activation and quantification of the contribution of diagnosis and cognitive domain to each cluster.

**Results:**

A total of 367 experiments (major depressive disorder, 149; bipolar disorder, 103; posttraumatic stress disorder, 55; and anxiety disorders, 60) were included comprising observations from 4507 patients and 4755 control individuals. Three right-sided clusters of hypoactivation were identified centered in the inferior prefrontal cortex/insula (volume, 2120 mm^3^), the inferior parietal lobule (volume, 1224 mm^3^), and the putamen (volume, 888 mm^3^); diagnostic differences were noted only in the putamen (χ^2^_3_ = 8.66; *P* = .03), where hypoactivation was more likely in bipolar disorder (percentage contribution = 72.17%). Tasks associated with cognitive systems made the largest contribution to each cluster (percentage contributions >29%). Clusters of hyperactivation could only be detected using a less stringent threshold. These were centered in the perigenual/dorsal anterior cingulate cortex (volume, 2208 mm^3^), the left amygdala/parahippocampal gyrus (volume, 2008 mm^3^), and the left thalamus (volume, 1904 mm^3^). No diagnostic differences were observed (χ^2^_3_ < 3.06; *P* > .38), while tasks associated with negative valence systems made the largest contribution to each cluster (percentage contributions >49%). All findings were robust to the moderator effects of age, sex, and magnetic field strength of the scanner and medication.

**Conclusions and Relevance:**

In mood disorders, posttraumatic stress disorder, and anxiety disorders, the most consistent transdiagnostic abnormalities in task-related brain activity converge in regions that are primarily associated with inhibitory control and salience processing. Targeting these shared neural phenotypes could potentially mitigate the risk of affective morbidity in the general population and improve outcomes in clinical populations.

## Key Points

**Question:**

Is the clinical overlap seen in major depressive disorder, bipolar disorder, anxiety disorders, and posttraumatic stress disorder reflected at the neurobiological level?

**Findings:**

In this meta-analysis of 226 task-related functional imaging studies, transdiagnostic clusters of hypoactivation were identified in the inferior prefrontal cortex/insula, inferior parietal lobule, and putamen.

**Meaning:**

Across mood and anxiety disorders, the most consistent transdiagnostic abnormalities in task-related brain activity converge in regions that are primarily associated with inhibitory control and salience processing.

## Teaser

This meta-analysis evaluates the presence of transdiagnostic convergence in abnormalities in task-related brain activation in patients with mood disorders, anxiety disorders, and posttraumatic stress disorder.

## Introduction

Mood disorders (major depressive disorder and bipolar disorder), posttraumatic stress disorder, and anxiety disorders (generalized anxiety disorder, panic disorder, agoraphobia, and specific and social phobia) are highly comorbid^1^ and collectively account for more than 65% of nonfatal disease burden attributable to psychiatric disorders.^2^ Up to 90% of patients with an anxiety disorder meet criteria for a concurrent mood disorder,^2,3^ and as many as 70% of individuals with mood disorders meet criteria for an anxiety disorder during their lifetime.^4,5^ Negative affective states are shared and central clinical features of these disorders,^6^ including bipolar disorder, where depressive symptoms are the dominant psychopathology.^7^

Meta-analyses of brain imaging studies on mood, posttraumatic stress, and anxiety disorders have shown that each of these disorders is associated with abnormalities in task-related brain engagement (summarized in eTable 1 in the Supplement). The findings of these diagnosis-specific meta-analyses show conspicuous divergence (eTable 1 in the Supplement) that has been attributed to low numbers of contributing studies, reporting bias from region-of-interest (ROI) analyses, and inadequate correction for multiple comparisons.^8,9^ Of note, methodological improvements over time have led to a progressive reduction in the number clusters of case-control differences reported in diagnosis-specific meta-analyses (eFigure 1 in the Supplement). Using data from task-related functional magnetic resonance imaging (fMRI) studies published in the last 15 years, we demonstrated that diagnostic differences in the brain regions implicated in mood and anxiety disorders largely reflected the association with ROI analyses.^9^ By contrast, when only whole-brain analyses were considered, there were large pairwise correlations between the diagnosis-specific profiles (ρ range, 0.79-0.82; all *P* < .001).^9^

Here, we extend this line of research in 2 distinct ways. First, we sought to identify brain regions where aberrant task-related activation was most likely to show transdiagnostic convergence across major depressive disorder, bipolar disorder, and anxiety and posttraumatic stress disorders. To achieve this, we capitalized on activation likelihood estimation (ALE) meta-analytic tools^10,11,12,13^ to synthesize coordinates of case-control differences in what is, to our knowledge, the largest sample of fMRI articles comprising the body of the relevant literature over the last 15 years. Second, we anchored the analysis plan to the Research Domain Criteria (RDoC) framework^14^ proposed by the US National Institute of Mental Health. The RDoC framework is the best approximation to a criterion approach to the classification of the array of activation tasks used in the primary studies and enables a principled interpretation of results in terms of dysfunction in clearly defined cognitive processes. Based on current neurobiological models,^15,16^ we predicted that transdiagnostic clusters of aberrant brain activity would converge in regions within the prefrontal, insular, and anterior cingulate cortex and in subcortical regions (particularly the amygdala/hippocampus and striatum) that support the adaptive regulation of cognition and affect.

## Method

### Literature Search and Article Eligibility

We applied the Preferred Reporting Items for Systematic Reviews and Meta-analyses criteria (http://www.prisma-statement.org/) to identify articles that used whole-brain analyses of task-related fMRI to compare healthy adults with adult patients who received a diagnosis of major depressive disorder, bipolar disorder, generalized anxiety disorder, panic disorder, agoraphobia, specific and social phobias, and posttraumatic stress disorder (details of the search and article eligibility criteria in the eMethods and eFigure 2 in the Supplement). Because we used data from published studies, no institutional review board approval was sought and patient consent was not obtained.

### Database Construction

We use the term *article* to denote the published manuscript and the term *experiment* to denote the coordinates of case-control differences reported in each article. Accordingly, from each article, we extracted coordinates of case-control differences derived from whole-brain analyses only. These were then coded according to the strength of the magnetic field of the scanner, the diagnostic classification system, symptom severity, the direction of change in brain activity in patients compared with healthy individuals (hypoactivation or hyperactivation), and the corresponding RDoC domain and construct. The coding of tasks according to their corresponding RDoC domain and construct is described in the eMethods and shown in eTable 2 in the Supplement. For example, tasks such as the n-back and the Sternberg were assigned to the construct of working memory and the domain of cognitive systems whereas various facial affect processing tasks were assigned to the construct of social communication and the domain of social processes. This allowed us to create 3 groupings of tasks labeled by their type (eg, facial affect processing) and their RDoC construct and domain. For each article, the symptom severity of the clinical sample was coded based on the mean psychopathology rating reported. To accommodate the use of different instruments across studies and clinical populations, symptom levels were labeled as minimal/mild, moderate, or severe (details in eMethods in the Supplement). Furthermore, for each experiment, but separately for patient and control groups, we coded their diagnostic status, sample size, age, and sex (percentage of men). In patients, medication status was coded as the percentage of patients receiving any psychotropic medication in each study sample. Further details of the database construction are provided in the eMethods in the Supplement.

### Activation Likelihood Estimation

We used ALE, implemented in MATLAB (MathWorks), to test whether the whole-brain coordinates of case-control differences across experiments and disorders converged into discrete clusters with a nonrandom spatial distribution.^10,11,12,13^ The fundamental assumption of the ALE is that each voxel has the same a priori chance of differentiating patients from control individuals (null hypothesis). Consequently, ROI analyses were excluded because they violate this assumption and their inclusion would artificially bias results in favor of voxels within these regions. The main outcome of the ALE analysis are the clusters (ie, grouping of brain regions) in which the coordinates of the experiments converge. Per best-practice standards,^10,11,12,13^ statistically significant clusters were identified using a cluster-level familywise error–corrected threshold of *P* less than .05 (cluster-forming threshold at voxel-level *P* < .001). Additionally, for each suprathreshold cluster, we extracted the per voxel probability of functional change from the modeled activation maps. These values represent the probability of identifying a functional change for a mean voxel within the clusters derived from the modeled activation maps. Details of the procedures involved are described in the eMethods in the Supplement.

We analyzed coordinates of hypoactivation or hyperactivation in patients compared with healthy individuals separately to enhance interpretability. First, we identified suprathreshold clusters of hypoactivation and hyperactivation by pooling coordinates from all diagnoses and tasks and then conducted follow-up analyses to identify the effect of moderators. For the follow-up analyses, we extracted per-voxel probabilities of functional change for each cluster and conducted nonparametric Kruskal-Wallis tests and Spearman correlations to calculate the contribution of age, sex, RDoC domain/construct, diagnosis, symptom severity, and medication.

In generating the modeled activation maps, we pooled coordinates across diagnoses for 2 reasons. First, the disorders considered here are highly comorbid and hence the pooled analyses accommodate uncertainty about their symptomatic and syndromal boundaries. Moreover, comorbidity is not always reported in primary studies and therefore it is difficult to estimate its prevalence in the samples examined and its potential contribution to the neuroimaging results. Second, pooling results across diagnoses balances power, specificity, and sensitivity and allows for a data-driven quantification of the diagnosis-specific contribution to each suprathreshold cluster. We conducted supplemental diagnosis-specific analyses, which are presented in the eMethods and eResults in the Supplement.

In generating the modeled activation maps, we pooled coordinates from all the tasks used in the primary experiments based on 2 considerations. First, traditional neuropsychologic formulations tend to consider cognitive tasks as relatively specific to a particular process. Advances in cognitive and affective neuroscience have led to the recognition that the association between brain structure and function is pluripotent (one-to-many) and degenerate (many-to-one).^17,18^ Therefore, any given task engages brain regions outside those predicted by the cognitive mechanisms attributed to that particular task, while a single brain area may be activated by disparate tasks that may not share cognitive components.^17,18^ Our approach accommodates pluripotency and offers a more realistic representation of the relevance of cognitive domains to case-control differences. Following the identification of suprathreshold clusters from the pooled analyses, we estimated the contribution of tasks to each cluster. For these follow-up analyses, tasks were grouped according to their assigned RDoC domain/construct; the use of the RDoC framework provided an organizing principle for the multitude of tasks used in the primary studies. Compared with other classifications that are primarily driven by convention, the RDoC framework has a clearly defined origin and rationale.^14^

Finally, we used an alternate meta-analytic algorithm to confirm the reproducibility of the results of the main analyses and conducted several ancillary meta-analyses focusing on each diagnosis separately and using alternate classification of tasks (described in the eMethods, eResults, and eTable 8 in the Supplement).

## Results

### Samples and Experiments

In total, 226 articles were selected (major depressive disorder, 83; bipolar disorder, 66; posttraumatic stress disorder, 35; generalized anxiety disorder, 6; panic disorder and agoraphobia, 6; specific phobias, 8; and social phobia, 22) comprising observations from 4507 patients and 4755 healthy individuals. Full citations and details of the selected articles are provided in eTables 3 to 5 in the Supplement. Given the small number of studies on generalized anxiety disorder, panic disorder, agoraphobia, and specific and social phobias, we used a single coding of “anxiety disorders” for experiments arising from these patient groups. The selected articles yielded a total of 367 experiments (major depressive disorder, 149; bipolar disorder, 103; posttraumatic stress disorder, 55; and anxiety disorders, 60) (Table). The percentage of patients receiving medication differed by diagnosis (χ^2^_3_ = 77.03; *P* < .001), being higher for bipolar disorder and major depressive disorder followed by posttraumatic stress disorder and anxiety disorders (eTable 6 in the Supplement). There were no statistical differences in the number of experiments per diagnosis (*F*_3_ = 2.54; *P* = .10) or per RDoC domain (*F*_4_ = 0.60; *P* = .66) and no significant case-control differences in age or sex.

**Table.** Experiments and Samples Included in the Database^a^

Diagnosis	Experiments, Total No.	Patients			Healthy Individuals		
		Sample, No.	Age, Mean (SD), y	Men, Mean (SD), %	Sample, No.	Age, Mean (SD), y	Men, Mean (SD), %
MDD	149^b^	1656	36.2 (9.85)	41 (17)	1759	33.7 (9.41)	43 (15)
BD	103^c^	1486	37.9 (10.52)	48 (21)	1642	36.4 (10.26)	47 (19)
PTSD	55^d^	557	35.0 (8.65)	44 (40)	574	34.5 (8.25)	43 (40)
ANX	60^e^	808	29.6 (7.4)	38 (23)	780	28.96 (7.07)	39 (23)

Abbreviations: ANX, anxiety disorders; BD, bipolar disorder; CD, cross domain; CS, cognitive systems; MDD, major depressive disorders; NVS, negative valence systems; PVS, positive valence systems; PTSD, posttraumatic stress disorder; RDoC, research domain criteria; SP, social processes.

^a^ There were no experiments that could be mapped to the domain of arousal. Experiment indicates set of coordinates of case-control differences originating from specific task contrasts; some published articles contributed more than 1 experiment (details in the eMethods and eTables 2-5 in the Supplement).

^b^ Of the 149 MDD experiments, CS was studied in 27; NVS, 41; PVS, 43; SP, 17; and CD, 21.

^c^ Of the 103 BD experiments, CS was studied in 49; NVS, 20; PVS, 11; SP, 7; and CD, 16.

^d^ Of the 55 PTSD experiments, CS was studied in 13; NVS, 12; PVS, 1; SP, 14; and CD, 14.

^e^ Of the 60 ANX experiments, CS was studied in 2; NVS, 20; PVS, 1; SP, 15; and CD, 22.

### Activation Likelihood Estimation

Coordinates of hypoactivation (179 experiments) or hyperactivation (188 experiments) in patients compared with healthy individuals were entered in separate meta-analyses. Each meta-analysis had more than 80% power to detect clusters of brain regions if they showed convergent case-control differences in at least 10 experiments.^10,11,12,13^ Robust estimation of moderator effects was possible for age, sex, strength of the magnetic field of the scanner, symptom severity, and medication status. The peak coordinates of the suprathreshold clusters are presented in Talairach space.

#### Transdiagnostic Clusters of Hypoactivation in Patients

**Figure 1.**
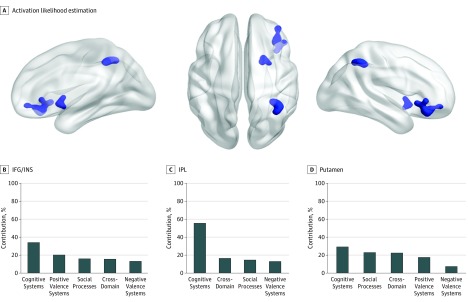
Transdiagnostic Clusters of Hypoactivation in Patients Relative to Healthy Individuals A, Activation likelihood estimation identified 3 transdiagnostic clusters of relative hypoactivation in patients centered on right inferior prefrontal cortex/insula (IFG/INS), the right inferior parietal lobule (IPL), and the right putamen. B, Percentage contribution of each research domain criteria (RDoC) to each cluster. Additional details are in eTable 7 in the Supplement.

We identified 3 reproducible (eFigure 4 in the Supplement) transdiagnostic clusters of hypoactivation in patients compared with healthy individuals centered on the right inferior prefrontal cortex/insula (peak coordinates: x = 40, y = 30, z = −10; volume, 2120 mm^3^), the right inferior parietal lobule (peak coordinates: x = 38, y = −48, z = 46; volume, 1224 mm^3^), and the right putamen (peak coordinates: x = 24, y = 8, z = −6; volume, 888 mm^3^) (Figure 1A; eFigure 3A in the Supplement). The effects of the moderator variables, including medication status and symptom severity, were not significant for any cluster (eResults in the Supplement).

There was no significant association of diagnosis with the prefrontal/insula (χ^2^_3_ = 6.22; *P* = .10) and inferior parietal clusters (χ^2^_3_ = 3.54; *P* = .31); an association of diagnosis was noted for the putamen (χ^2^_3_ = 8.66; *P* = .03), for which the contribution of bipolar disorder (72.17%) was greater than that of major depressive disorder (17.35%; *z*_3_ = 2.28; *P* = .02), posttraumatic stress disorder (4.55%; *z*_3_ = 1.82; *P* = .06) and anxiety disorders (5.93%; *z*_3_ = 2.07; *P* = .03); all other pairwise comparisons were not significant.

Differences in the contribution of RDoC domains/constructs did not reach statistical significance for any cluster, although processes associated with cognitive systems (Figure 1B), and particularly the construct of cognitive control, made the largest contribution to each cluster (eTable 7 in the Supplement). Of note, hypoactivation in patients in the right inferior frontal gyrus/insula was also identified in an ancillary meta-analysis restricted only to tasks that involve affective (ie, acute or potential threat, reward attainment, approach motivation, and frustrative nonreward) and social (ie, social communication and perception of threat) processing (eResults in the Supplement), thus confirming the importance of this cluster across multiple domains of cognition. No additional diagnosis-specific clusters were identified (eResults in the Supplement).

#### Transdiagnostic Clusters of Hyperactivation in Patients

Despite adequate power, there were no statistically significant clusters of hyperactivation in patients compared with healthy individuals at cluster-level familywise error–corrected threshold of *P* less than .05 (cluster-forming threshold at voxel-level *P* < .001). At the same threshold, no suprathreshold clusters were detected when we repeated the analyses including only those experiments involving affective and social processing (eResults in the Supplement). No diagnosis-specific clusters were identified either (eResults in the Supplement). This was unexpected given that current models emphasize hyperactivation, primarily during the processing of emotionally valenced stimuli, in the patient populations considered here.^15,16^

**Figure 2.**
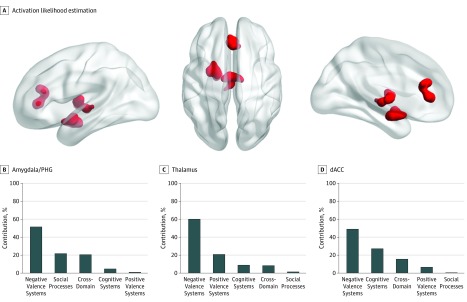
Transdiagnostic Clusters of Hyperactivation in Patients Compared With Healthy Individuals A, Activation likelihood estimation identified 3 transdiagnostic clusters of hyperactivation in the left amygdala/parahippocampal gyrus (PHG), the left thalamus, and the perigenual/dorsal anterior cingulate cortex (dACC). B, Percentage contribution of each research domain criteria (RDoC) to each cluster. Additional details are in eTable 7 in the Supplement.

Clusters of hyperactivation could only be detected using uncorrected voxel-level thresholding (*P* < .01) combined with an extent threshold of greater than 200 mm^3^. This level of statistical inference increases sensitivity at the cost of consistency because it magnifies contributions originating from only a few studies; nevertheless, it can still be considered acceptable if more than 20 experiments are modeled, as is the case here.^10,11,12,13^ The clusters thus identified were centered in the left amygdala/parahippocampal gyrus (peak coordinates: x = −22, y = −2, z = −15; volume, 2208 mm^3^), the left thalamus (peak coordinates: x = −2, y = −12, z = 4; volume, 2008 mm^3^) and the perigenual/dorsal anterior cingulate cortex (peak coordinates: x = 0, y = 34, z = 12; volume, 1904 mm^3^) (Figure 2A; eFigure 3B in the Supplement). For the latter cluster, there was a negative correlation with the percentage of men (ρ = −0.68; *P* = .004), but no other moderator effect (including medication and symptom severity) was significant for this or the other clusters (details in the eResults in the Supplement). There was no significant difference in the degree to which each diagnosis contributed to the left amygdala/parahippocampal gyrus (χ^2^_3_ = 2.13; *P* = .54), the left thalamus (χ^2^_3_ = 1.26; *P* = .73), or the perigenual/dorsal anterior cingulate cortex (χ^2^_3_ = 3.06; *P* = .38). Differences in the contribution of RDoC domains did not reach statistical significance for any cluster, although experiments associated with negative valence systems (Figure 2B), and particularly the construct of acute threat (eTable 7 in the Supplement), made the largest numerical contribution to each of these 3 clusters.

## Discussion

Meta-analyses of 367 task-related fMRI experiments in mood disorders, posttraumatic stress disorder, and anxiety disorders, comprising data from 4507 patients and 4755 control individuals, detected statistically robust transdiagnostic clusters of hypoactivation in the inferior prefrontal cortex/insula, the inferior parietal lobule, and the putamen. These regions are part of a right-dominant brain system that supports contextual shifting and stopping of mental operations and behavioral responses.^19,20,21,22,23,24,25,26^ Specifically, the right inferior prefrontal cortex is critically involved in the inhibition of contextually inappropriate cognitive, affective, and motor responses^19,20,21^; similarly, the putamen, particularly on the right, is essential for terminating contextually inappropriate motor and cognitive processes.^22^ The anterior insula^23,24^ and the right inferior parietal lobule^25,26^ participate in the generation of salience-related signals that either initiate or terminate the engagement of attentional and working memory networks in response to changing demands. The insula, and particularly the anterior portion on the right, is thought to have a major role in integrating interoceptive information with information from other brain regions, thus supporting the formation of the conscious experience of an embodied self.^27,28^ This awareness of negative emotional states may act as a salient trigger for the insula and the adjacent inferior frontal regions to engage mechanisms of cognitive control. Notably, experiments involving domains of nonaffective cognition, affective processing, and social cognition showed a similar range of contributions to these clusters of hypoactivation (respective range: 28%-50% and 29%-56%) (Figure 1B; eTable 7 in the Supplement). We therefore infer that the dominant abnormality in mood disorders, posttraumatic stress disorder, and anxiety disorders involves a diagnosis-general disruption in salience processing (including interoceptive processing) and inhibitory control. These results contradict early hypotheses, which stipulated that affective morbidity results from right-sided fronto-parietal hyperactivity in response to negative/withdrawal stimuli,^29^ but are in line with evidence that emphasizes the role of deficient cognitive control.^30,31,32^ Further support derives from studies showing that deficits in the ability to stop and shift ongoing affective states and thoughts are the most significant predictors of affective symptoms and syndromes.^33,34,35^ Neurocognitive studies in mood and anxiety disorders also indicate a general disruption in cognitive control because they consistently report deficits of large effect size in stopping and shifting responses in a range of tasks.^32,36,37^ Thus, impaired engagement of brain regions that subserve salience processing and inhibitory control present a plausible explanatory mechanism for the affective and nonaffective abnormalities observed in patients. In a separate meta-analysis^38^ of functional neuroimaging studies that was limited only to tasks of cognitive control, hypoactivation in the right inferior prefrontal/insular cortex was also reported as a transdiagnostic feature of schizophrenia, bipolar disorder, major depressive disorder, anxiety disorders, and substance use.^38^ When considered together, these findings point to the possibility that abnormalities in brain regions involved in switching and stopping may underpin the vulnerability to develop any and all forms of psychopathology. Interestingly, similar arguments have been put forward for a single dimension of psychopathology, termed factor *p*, as a main predictor of individuals’ liability for all mental disorders.^39^ The relationship between the *p* factor and disrupted engagement in salience/inhibitory control regions presents an intriguing avenue for future research.

We also identified 3 transdiagnostic clusters of hyperactivation in patients compared with healthy individuals centered in the left amygdala/parahippocampal gyrus, the left thalamus, and the perigenual/dorsal anterior cingulate cortex that were attributable mainly to experiments mapping to RDoC domains relating to affective and social processing (Figure 2B). The clusters identified appear plausible because they comprised regions consistently associated with affective morbidity.^15,16^ The perigenual/dorsal anterior cingulate cortex is known to exert a regulatory influence on emotional experience and appraisal^40^ while the amygdala and parahippocampal gyrus, particularly on the left, are involved in emotional memory formation and retrieval.^41^ The dorsal anterior cingulate cortex is also closely involved in the generation of internal autonomic and their associated expressive emotional responses.^42^ Its relative hyperactivation in patients is consistent with the notion of increased arousal in response to stress that may be a trait feature of mood and anxiety disorders but may also reflect increased stress response to the fMRI tasks.^43^

Notwithstanding, these clusters were only detectable at a liberal statistical threshold, indicating greater inconsistency across primary studies that may indicate that hyperactivation in patients compared with healthy individuals may be more sensitive to variations in fMRI task design (eg, type or duration of stimuli or task instructions) and neuroimaging acquisition and analysis parameters. Detailed investigations that could directly address these issues would require more data than are currently available in the entire literature corpus.

### Limitations

We placed substantial emphasis on the rigor and reproducibility of our methods to address ongoing concerns about the disparity in the number and localization of clusters in previous meta-analyses (eTable 1 in the Supplement). To further enhance reproducibility, we classified experiments based on the RDoC framework, which offers a structured approach to classification for fMRI tasks in future studies. We only included studies in adults, and therefore these findings may not generalize to pediatric or geriatric groups. We did not consider studies that failed to find case-control differences because such practice could only be justified if negative studies were sufficiently powered. We did not find an effect of symptom severity on the transdiagnostic clusters. This observation should be viewed with caution because of the variable instruments used to rate psychopathology and the reliance on group means from each study sample. Medication status did not have a statistically significant moderator association with the results reported. Medication has been shown to have mostly normalizing effects^44^ and may have attenuated case-control differences in the primary studies. We examined disorders with significant symptomatic and syndromal overlap for which we had comparable amount of data across diagnoses. We decided to exclude task-related fMRI articles on schizophrenia because the disproportionately larger number of studies (>250)^9^ would have skewed the results. Given the observed power in this study, the results are statistically robust, but as the literature expands it is possible that additional transdiagnostic or disease specific clusters may emerge.

## Conclusions

This meta-analysis of what is, to our knowledge, the largest data set of fMRI studies currently available identified reduced engagement of brain regions associated with inhibitory control and salience processing as the most consistent neurobiological feature in mood disorders, posttraumatic stress disorder, and anxiety disorders. These shared brain phenotypes have the potential to serve as targets for interventions aiming to improve clinical outcomes and reduce or prevent affective morbidity in the general population. Tracking the trajectory of disruption in these regions across development could provide invaluable information regarding their timing and their association with emerging psychopathology and psychiatric nosology.

## Notes

Supplement. **eTable 1.** Results of the meta-analyses of functional neuroimaging studies in mood, posttraumatic stress, and anxiety disorders **eFigure 1.** Number of clusters of convergent case-control differences identified in meta-analyses of functional neuroimaging studies in mood, anxiety and post-traumatic stress disorders **eMethods.** **eFigure 2.** Preferred Reporting Items for Systematic Reviews and meta-analyses (PRISMA) Flow Diagram **eTable 2.** Classifications of tasks according to the Research Domain Criteria **eResults.** **eFigure 3.** Results of the main analysis **eFigure 4.** Results of the reproducibility analysis **eReferences**
