# Supplementary material for: Shared Neural Phenotypes for Mood and Anxiety Disorders: A Meta-analysis of 226 Task-Related Functional Imaging Studies
Source: JAMA Psychiatry. 2019 Oct 30;77(2):172–9. doi: 10.1001/jamapsychiatry.2019.3351 (PMC6822098; doi:10.1001/jamapsychiatry.2019.3351)
Supplement: Supplement. — eTable 1. Results of the meta-analyses of functional neuroimaging studies in mood, posttraumatic stress, and anxiety disorders eFigure 1. Number of clusters of convergent case-control differences identified in meta-analyses of functional neuroimaging studies in mood, anxiety and post-traumatic stress disorders eMethods. eFigure 2. Preferred Reporting Items for Systematic Reviews and meta-analyses (PRISMA) Flow Diagram eTable 2. Classifications of tasks according to the Research Domain Criteria eResults. eFigure 3. Results of the main analysis eFigure 4. Results of the reproducibility analysis eReferences [file jamapsychiatry-77-172-s001.pdf]

Supplementary Online Content

Janiri D, Moser DA, Doucet GE, et al. Shared neural phenotypes for mood and anxiety disorders: a meta-analysis of 226 task-related functional imaging studies . Published online October 30, 2019. *JAMA Psychiatry*. doi:10.1001/jamapsychiatry.2019.3351

eTable 1. Results of the meta-analyses of functional neuroimaging studies in mood, post-traumatic stress and anxiety disorders

eFigure 1. Number of clusters of convergent case-control differences identified in meta-analyses of functional neuroimaging studies in mood, anxiety and post-traumatic stress disorders

eMethods.

eFigure 2. Preferred Reporting Items for Systematic Reviews and meta-analyses (PRISMA) Flow Diagram

eTable 2. Classifications of tasks according to the Research Domain Criteria

eTable 3. Major depressive disorder: studies included

eTable 4. Bipolar disorder: studies included

eTable 5. Post-traumatic stress disorder and anxiety disorders: studies included

eResults.

eTable 6. Experiments and samples included in the analysis on affective experiments

eTable 7. Percentage contribution of RDoC construct to each transdiagnostic suprathreshold cluster

eTable 8. Percentage contribution of tasks to each transdiagnostic suprathreshold cluster

eFigure 3. Results of the main analysis

eFigure 4. Results of the reproducibility analysis

eReferences

This supplementary material has been provided by the authors to give readers additional information about their work.

© 2019 Janiri D et al. *JAMA Psychiatry.*

© 2019 Janiri D et al. *JAMA Psychiatry.*

eIntroduction

eTable 1. Results of previous meta-analyses of functional neuroimaging studies in mood, post-traumatic stress and anxiety disorders					
**Author, Year [PubMed PMID]**	**Patients/ Controls**	**Studies Included**	**Tasks**	**Regions of Hypoactivation in Patients**	**Regions of Hyperactivation in Patients**
**Major Depressive Disorder**					
Fitzgerald, 2008 [1] [17598168]*	76/76	6	Various tasks involving processing of affective stimuli	R/L Inferior Frontal Gyrus, R/L Anterior Cingulate, R Inferior Temporal Gyrus, R Fusiform Gyrus, L Posterior Cingulate, R/L Cerebellum, R Putamen, R Superior Frontal Gyrus, L Middle Frontal Gyrus, R Middle Temporal Gyrus, L Superior Temporal Gyrus, L Insula	R Superior Frontal Gyrus, R Medial Frontal Gyrus, R/L Inferior Frontal Gyrus, R/L Anterior Cingulate, R Frontal Lobe, L Precentral Gyrus, R/L Cingulate Gyrus, R Lingual Gyrus, R Middle Frontal Gyrus, R Insula, R/L Fusiform Gyrus, L Precuneus, R/L Inferior Parietal Lobule, L Middle Temporal Gyrus, L putamen, L Amygdala/Uncus, R Cerebellum
Hamilton, 2012 [2] [22535198]*	351/354	24	Various tasks involving processing of affective stimuli	R/L Middle Frontal Gyrus, R Caudate, L Cerebellum	L/R Thalamus, R Amygdala, L Anterior Cingulate, L Temporal Lobe, L Precentral Gyrus, L Middle Temporal Gyrus
Del Vecchio, 2012 [3] [21820878]	225/220	11	Facial affect processing tasks	R Putamen, L Caudate	R Parahippocampal gyrus/Amygdala
Diener, 2012 [4] [22521254]	558/569	40	Affective tasks: Various tasks involving processing of affective stimuli and emotion regulation and reward processing tasks<br>Cognitive tasks: executive function, verbal fluency, working memory, memory, cognitive control, oddball.	L Medial Frontal Gyrus/R Paracentral lobule, L Anterior Cingulate, R Insula, L Postcentral Gyrus, L Posterior Lobe, L Middle occipital Gyrus/ L Precuneus, R Caudate	R Inferior Frontal Gyrus, R Superior Frontal Gyrus, R Superior Temporal Gyrus, L Medial Globus Pallidus, L Thalamus

© 2019 Janiri D et al. *JAMA Psychiatry.*

**eTable 1. Results of previous meta-analyses of functional neuroimaging studies in mood, post-traumatic stress and anxiety disorders**					
**Author, Year [PubMed PMID]**	**Patients/ Controls**	**Studies Included**	**Tasks**	**Regions of Hypoactivation in Patients**	**Regions of Hyperactivation in Patients**
Graham, 2013 [5] [23890584]	566/580	34	Various affective and non-affective cognitive tasks	R Middle Frontal Gyrus, R Parahippocampal Gyrus, L Inferior Frontal Gyrus, L Caudate, R Middle Temporal Gyrus, R Medial Frontal Gyrus, R Insula, L Superior Temporal Gyrus, R Parahippocampal Gyrus/Amygdala, L Cuneus	L Middle Temporal Gyrus, L Middle Frontal Gyrus, L Anterior Cingulate, L Precentral Gyrus, L Thalamus, L Cingulate Gyrus, R Superior Temporal Gyrus
Zhang, 2013 [6] [23856280]	341/367	22	Tasks involving processing of positive affective stimuli and monetary reward	L/R Caudate, L Thalamus, R/L Cerebellum, R Putamen, L Aterior Cingulate, L insula	R/L Cuneus, R Frontal Lobe, R/L Middle Frontal Gyrus, L Superior Frontal Gyrus, L Fusiform Gyrus, R Lingual Gyrus
Lai, 2014 [7] [24275771]	175/154	8	Facial affect processing tasks	L Cingulate Gyrus, R Thalamus, L Anterior Cingulate, R Superior Frontal Gyrus	L Putamen, L Parahippocampal Gyrus
Palmer, 2015 [8] [25642179]*	643/653	44	Various tasks involving processing of affective stimuli	R Anterior Cingulate/Putamen, R Parahippocampal Gyrus/Amygdala, R Inferior Frontal Gyrus, L Thalamus	L Thalamus/ Parahippocampal Gyrus, L amygdala, L Globus Pallidus, L Medial Frontal Gyrus, L Putamen, R Middle Frontal Gyrus, R Thalamus
Wang, 2015 [9] [25174680]	160/203	11	Working memory tasks	R Insula, R Precentral gyrus, R Precuneus	L Inferior frontal gyrus, L Middle frontal gyrus, L Precentral gyrus, L Insula, R Supramarginal gyrus, R Superior temporal gyrus
Müller, 2017 [10] [27829086]	1083/1155	59	Various affective and non-affective cognitive tasks	-	-
Keren, 2018 [11] [29921146] [‡]	580 (patients and controls)	26	Reward tasks	L Caudate	
**Bipolar Disorder**					

© 2019 Janiri D et al. *JAMA Psychiatry.*

eTable 1. Results of previous meta-analyses of functional neuroimaging studies in mood, post-traumatic stress and anxiety disorders					
**Author, Year [PubMed PMID]**	**Patients/ Controls**	**Studies Included**	**Tasks**	**Regions of Hypoactivation in Patients**	**Regions of Hyperactivation in Patients**
Chen, 2011 [12] [21320248]	794/823	51	Affective tasks: facial affect task, processing prosody, affect induction and affective interference tasks Cognitive tasks: motor response, attention, working memory, and language	R Lingual Gyrus, R/L Inferior Frontal Gyrus, R/L Putamen, Cingulate Gyrus, L Anterior Cingulate, R Cerebellum, R Parahippocampal Gyrus/Amygdala	R/L Parahippocampal Gyrus/ Amygdala, R/L Middle Frontal Gyrus, L Caudate, R Precentral Gyrus, R Cerebellum, L Superior Frontal Gyrus, L Superior Temporal Gyrus, L Thalamus, L Medial Frontal Gyrus
Houenou, 2011 [13] [21470688]	139/142	13	Various tasks involving processing of affective stimuli	R Superior Frontal Gyrus, R Inferior Frontal Gyrus, R Precuneus, R Thalamus, R Cerebellum	R Middle Frontal Gyrus, L Caudate, L Thalamus, L Parahippocampal gyrus/Amygdala
Del Vecchio, 2012 [3] [21820878]	168/148	11	Facial affect processing tasks	R/L Inferior Frontal Gyrus	R/L Parahippocampal gyrus/Amygdala, L Putamen, L Thalamus
Del Vecchio, 2013 [14]	267/204	13	Facial affect processing tasks	R/L Inferior Frontal Gyrus, L Anterior Cingulate, R Insula	R/L Parahippocampal Gyrus/amygdala, L Parahippocampal Gyrus, L Thalamus
Hajek, 2013 [15] [24070910]	635/667	30	Response inhibition tasks	R Inferior Frontal Gyrus, L Putamen, L Precuneus, L Postcentral Gyrus	-
Alústiza, 2017[16] [28169089]	387/385	12	Various cognitive tasks	R Inferior Frontal Gyrus, R Caudate, R Inferior Parietal Lobule, L Parahippocampal Gyrus, L Inferior Frontal Gyrus, L Posterior Cingulate	L Middle Frontal Gyrus, L Anterior Cingulate, R Superior Temporal Gyrus
**Anxiety Disorders and Post-Traumatic Stress Disorder (PTSD)**					
Etkin, 2007 [17] [17898336]*	357/324	30	Tasks involving processing of negative emotional states and fear-conditioning tasks	**PTSD:** L Fusiform Gyrus, R Medial Frontal Gyrus, L Parahippocampal Gyrus, L Middle Temporal Gyrus, L Lentiform Nucleus, L Thalamus,	**PTSD:** R/L Amygdala, R Insula, R Inferior Parietal Lobule, L Cingulate Gyrus

© 2019 Janiri D et al. *JAMA Psychiatry.*

**eTable 1. Results of previous meta-analyses of functional neuroimaging studies in mood, post-traumatic stress and anxiety disorders**					
**Author, Year [PubMed PMID]**	**Patients/ Controls**	**Studies Included**	**Tasks**	**Regions of Hypoactivation in Patients**	**Regions of Hyperactivation in Patients**
				R Superior Frontal Gyrus, R Putamen, L Middle Occipital Gyrus, L /R Medial Frontal Gyrus, L Superior Frontal Gyrus, R Cingulate Gyrus,	**Social Anxiety:** L Parahippocampal Gyrus/ Amygdala, R Parahippocampal Gyrus, L Thalamus, R/L Insula, R Inferior Frontal Gyrus, L Middle Temporal Gyrus, **Specific Phobia:** R Parahippocampal Gyrus/Amygdala, L Lentiform Nucleus, R Lingual Gyrus, L Claustrum, R Insula, L Cingulate Gyrus
Hayes, 2012 [18] [22738125]*	246/258	21	Symptom provocation tasks, negative cognitive-emotional tasks	**PTSD:** R Anterior Cingulate, R/L Thalamus, R Inferior Frontal Gyrus, L Middle Occipital Gyrus, R Medial Frontal Gyrus, R Middle Temporal Gyrus, R Superior Parietal Lobule, R Cerebellum, L Fusiform Gyrus, R Cuneus, L Superior Temporal Gyrus	**PTSD**: R Anterior Cingulate, R Cingulate Gyrus, L Superior Temporal Gyrus, L Frontal Lobe
Hattingh, 2013 [19] [23335892]	91/93	7	Processing affective social stimuli	-	**Social Anxiety:** R Parahippocampal Gyrus/Amygdala/Globus Pallidus, L Parahippocampal gyrus/Amygdala, L Parahippocampal gyrus R Cingulate Gyrus, R Postcentral Gyrus
Ramage, 2013 [20] [22936519]* ‡	170/104	8	Traumatic imagery tasks	**PTSD**: R Cingulate Gyrus, L Posterior Cingulate, L Precuneus	
Sartory, 2013 [21] [23536785]*	274/145	19	Symptom provocation tasks	**PTSD:** R Superior/Middle/Inferior Temporal Gyrus, L Middle Occipital Gyrus, R/L Postcentral Gyrus L Middle Temporal Gyrus	**PTSD:** R/L Posterior Cingulate, L Precuneus, L Caudate, R Medial Frontal Gyrus, R Superior Parietal Lobule, L Precentral Gyrus
Ipser, 2013 [22] [23711114]*	202/127	13	Symptom provocation tasks	**-**	**Specific phobia:** L Globus Pallidus, R Thalamus, L Insula, R Cerebellum
Stark, 2015 [23] [26192104] ‡	418/426	25	Various tasks processing trauma and	**PTSD**: L Globus Pallidus, L/R Putamen, R insula, R Postcentral Gyrus, R Caudate, R Inferior Temporal Gyrus, L Parahippocampal Gyrus, L Fusiform	

© 2019 Janiri D et al. *JAMA Psychiatry.*

eTable 1. Results of previous meta-analyses of functional neuroimaging studies in mood, post-traumatic stress and anxiety disorders					
**Author, Year [PubMed PMID]**	**Patients/ Controls**	**Studies Included**	**Tasks**	**Regions of Hypoactivation in Patients**	**Regions of Hyperactivation in Patients**
			non-trauma related affective stimuli	Gyrus; these regions were differentially engaged in patients in a number of contrasts based on exposure status of controls	
Boccia, 2016 [24] [25913645]* ‡	815 (patients and controls)	16	Cognitive tasks: working memory, declarative episodic memory, executive function Affective: processing of affective stimuli including pain and mental imagery of trauma-related images	**PTSD:** R Anterior Cingulate/R Cingulate Gyrus, R Posterior Cingulate Gyrus/ Cingulate Gyrus/Precuneus, L Claustrum, R/L Insula, R/L Thalamus, R , Inferior Frontal Gyrus, R/L Medial Frontal Gyrus, R Inferior Parietal Lobule, R Hippocampus, R/L Anterior Cingulate, R/L Superior Temporal Gyrus, R Parahippocampal Gyrus, L Middle Frontal Gyrus; these regions were differentially engaged in patients in a number of contrasts based on trauma type	
Gentili, 2016 [25] [26341469]	449/424	23	Face processing tasks	**Social Anxiety:** L Posterior Cingulate, R Lingual Gyrus	**Social Anxiety:** R Inferior Frontal Gyrus, R/L Medial Frontal Gyrus, L Superior Frontal Gyrus, L Subcallosal Gyrus, R Globus Pallidus, L Amygdala, L Middle Temporal Gyrus, R Middle Occipital Gyrus, R/L Superior Temporal Gyrus, R Inferior occipital Gyrus
Wang, 2018 [26] [29785110]	219/227	8	Emotion regulation tasks	**Anxiety Disorder:** L Medial Frontal Gyrus, L Postcentral Gyrus, L Lentiform Nucleus, L Lingual Gyrus, R Precuneus, L Middle Frontal Gyrus	-
Regions were identified with Talairach Daemon based on the coordinates provided in each meta-analysis; Sample size and results reported here are refer only to whole-brain analyses based on activation likelihood estimation; L=Left R=Right; * included studies that used Positron Emission Tomography and/or Single-Photon Emission Computed Tomography in addition to functional magnetic resonance imaging studies; ‡ direction of case-control differences not clearly defined					

© 2019 Janiri D et al. *JAMA Psychiatry.*

eFigure 1. Number of clusters of convergent case-control differences identified in meta-analyses of functional neuroimaging studies in mood, anxiety and post-traumatic stress disorders

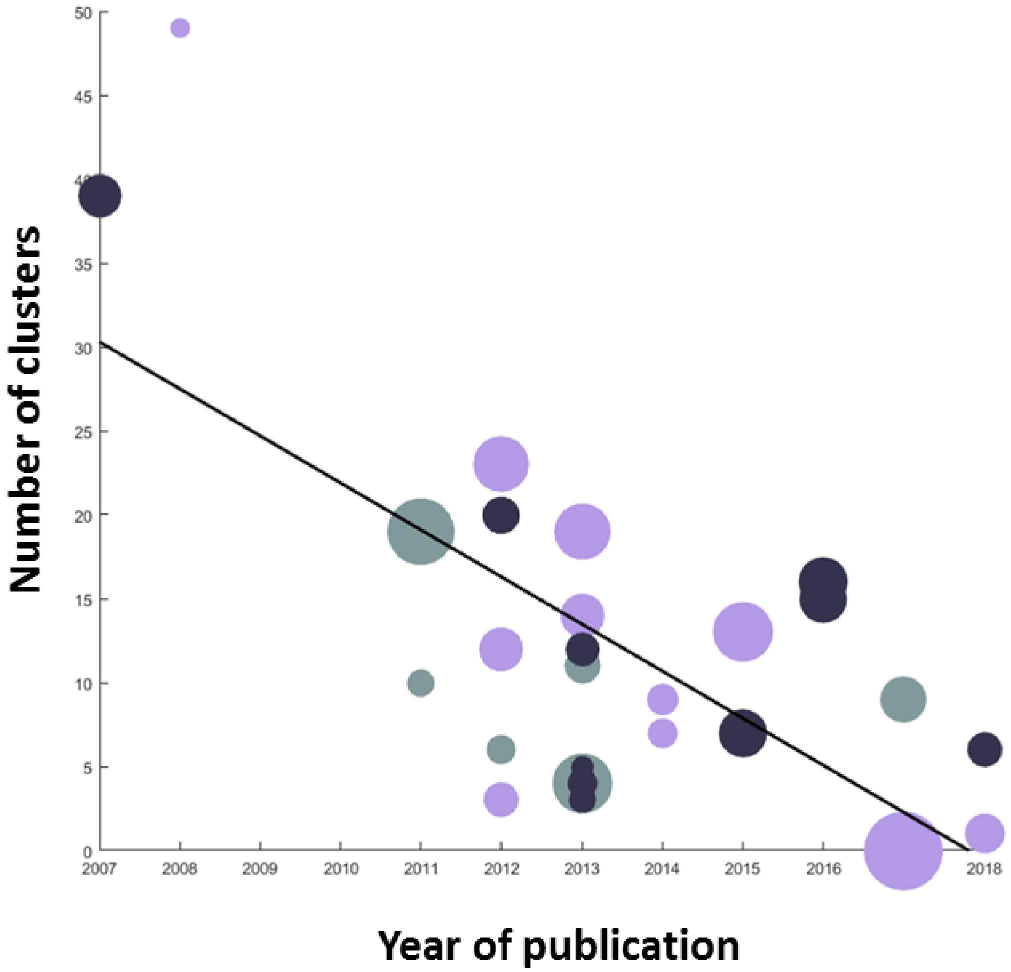

The meta-analyses presented in this graph are detailed in eTable 1. The x-axis depicts the year of publication of each meta-analysis. The y-axis indicates the number of clusters reported in each meta-analysis. The size of the circles indicates the number of participants included in the meta-analysis. Violet= meta-analyses on major depressive disorder; Green= meta-analyses on bipolar disorder; Blue= meta-analyses focusing on anxiety / post-traumatic stress disorders.

© 2019 Janiri D et al. *JAMA Psychiatry.*

eMethods

1. Literature search

We conducted a systematic literature search in accordance with the Preferred Reporting Items for Systematic Reviews and meta-analyses criteria (http://www.prisma-statement.org/) (eFigure 2) to identify functional magnetic resonance imaging (fMRI) articles published between January 1, 2005 and December 31, 2016 in PubMed (http://www.pubmed.org), Web of Science (https://apps.webofknowledge.com) and Google Scholar (https://scholar.google.de). The search keywords were "Major Depressive Disorder", "Unipolar Depression", "Bipolar Disorder", "Mania", "Anxiety", "Generalized Anxiety Disorder", "Social Anxiety Disorder", "Post Traumatic Stress Disorder", "Panic Disorder", "Specific Phobia", "Agoraphobia", "Functional", "Task", "MRI", "Emotion", "Emotional", "Affective", "Cognitive", "Executive", "Control," "Inhibition", and "Memory", and their various combinations and permutations. Further articles were identified by reference tracing of retrieved papers and previous meta-analyses of functional neuroimaging studies of major depressive disorder, bipolar disorder, post-traumatic stress disorder and anxiety disorders.

2. Eligibility Criteria for Article Selection

We included articles that (a) examined adults aged 18-65 years; (b) used the diagnostic criteria of the Diagnostic and Statistical Manual of Mental Disorders (DSM) or the International Statistical Classification of Diseases and Related Health Problems (16); (c) studied healthy individuals and patients with major depressive disorder, bipolar disorder, generalized anxiety disorder, panic disorder, agoraphobia, specific and social phobias and post-traumatic stress disorder as separate groups; (d) investigated case-control differences originating from tasks comparing an active to a control condition, rather than to rest; (e) reported case-control differences arising from whole-brain analyses as coordinates in Talairach or Montreal Neurological Institute standard reference space. When the same sample was studied longitudinally (either in observational or interventional designs) using the same experiments we only included the article that reported the baseline findings. When articles reported results from overlapping samples, we included the article with the largest sample size.

Three authors (DJ, DAM, SF) independently reviewed all the articles to determine eligibility (pairwise interclass correlations >0.91) and reach consensus about inclusion in cases of divergence in opinion.

3. Results of Literature Search

The 226 articles identified through the literature search comprised 83 articles on major depressive disorder, 66 on bipolar disorder, 35 on post-traumatic stress disorder, 6 on generalized anxiety disorder, 6 on panic disorder and agoraphobia, 8 on specific phobia and 22 on social phobia. Of the 66 articles on bipolar disorder, 47 included patients with bipolar disorder type I, 5 included patients with bipolar disorder type II, and 14 did not provide information about type. Of the 66 articles on bipolar disorder, 45 included patients that were described as not psychotic at the time of scanning, 2 included patients with psychotic features and 19 did not provide information regarding psychotic features in their sample.

© 2019 Janiri D et al. *JAMA Psychiatry.*

4. eFigure 2. Preferred Reporting Items for Systematic Reviews and meta-analyses (PRISMA) Flow Diagram

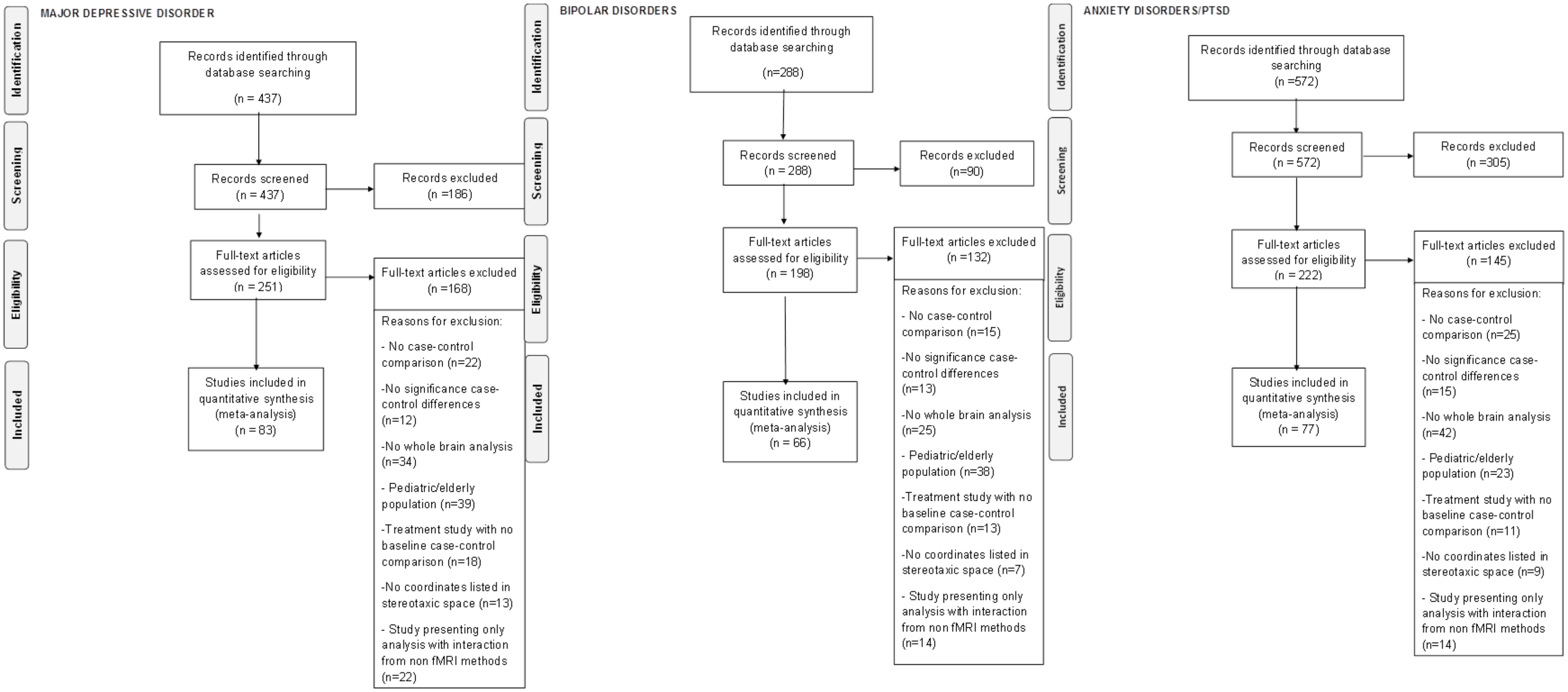

© 2019 Janiri D et al. *JAMA Psychiatry.*

5. Classifications of tasks according to the Research Domain Criteria

Three coauthors (DJ, DAM, SF) independently reviewed all the articles and classified the tasks and contrasts according to how RDoC defined domains and constructs (https://www.nimh.nih.gov/research-priorities/rdoc/constructs/rdoc-matrix.shtml) including outputs of the RDoC working groups. The pairwise interclass correlations were high (>0.88). Differences or uncertainty in classification of a task were resolved following review of other imaging and non-imaging studies that had employed such tasks in order to assess the general consensus within the wider research community about the mechanisms involved, and hence the most appropriate corresponding RDoC domains/constructs.

eTable 2. Classifications of tasks according to the Research Domain Criteria (RDoC)

RDoC Domain	RDoC Construct	Tasks
**Cognitive Systems**	**Attention**	Visual/Auditory oddball task, Attention shifting tasks, Continuous performance task
	**Memory**	Episodic memory encoding/retrieval tasks, Semantic list learning tasks, Paired associates learning tasks
	**Language**	Verbal fluency tasks, Semantic judgment tasks, Sentence Completion tasks
	**Cognitive Control**	Stroop task, Go/no-go task/ Stop task, Tower of London, Switching tasks
	**Working Memory**	N-back tasks, Sternberg task, Delayed-(non)-match-to-sample task
	**Cognitive Unclassified Tasks**	Implicit Learning tasks, Mental Arithmetic tasks
**Positive Valence Systems**	**Approach/Motivation**	Various tasks involving passive viewing of positively valenced scenes, words and facial expressions, Monetary incentive delay task (Reward expectation), Probabilistic reward task/Card-guessing task (Reward expectation)
	**Reward Attainment**	Monetary incentive delay task (Reward outcome); Reversal learning task (Reward outcome)
**Negative Valence Systems**	**Acute Treat**	Fear conditioning, Symptom provocation
	**Potential Threat**	Various tasks involving passive viewing of negatively valenced scene, words and facial expressions, Monetary incentive delay task (Loss expectation)
	**Frustrative Non-reward**	Monetary incentive delay task (Punishment), Reversal learning task (Punishment), Probabilistic reward task (Punishment)
**Social Processes**	**Social Communication**	Various facial affect processing tasks
	**Perception and Understanding of Self**	Various tasks involving processing of self-referential information
	**Perception and Understanding of Others**	Theory of Mind tasks
	**Affiliation and Attachment**	Cyberball task
**Cross Domain tasks**	**Cross Domain**	Affective Go/NoGo, Emotional Stroop, Emotional identity task, Emotional Working memory task, Emotional Verbal Memory task, Emotional oddball task, Emotion attention shifting task, Emotion regulation task

6. Full Citation and details of Selected Articles

© 2019 Janiri D et al. *JAMA Psychiatry.*

eTable 3: Major Depressive Disorder: Studies included									
**First Author**	**Year**	**Title**	**Publication**	**PMID**	**Patients' Symptom Severity**	**Task**	**RDoC Domain**	**RDoC Construct**	**Contrasts Used**
**Studies using experiments mapping the RDoC domains of negative valence systems, positive valence systems and social processes**									
Abler	2007	Anticipation of aversive stimuli activates extended amygdala in unipolar depression.	J Psychiatr Res. 2007 Sep;41(6):511-22. DOI: 10.1016/j.jpsychires.2006.07.020	17010993	HAMD: 18.5(NA)	Negative valence during passive scene viewing	Negative Valence	Potential Threat ("Anxiety")	Presentation negative vs neutral stimuli: Depressive Patients > Healthy controls
						Positive valence during passive scene viewing	Positive Valence	Approach Motivation	Presentation positive vs neutral stimuli: Depressive Patients > Healthy controls
Admon	2015	Striatal Hypersensitivity During Stress in Remitted Individuals with Recurrent Depression.	Biol Psychiatry. 2015 Jul 1;78(1):67-76. doi: 10.1016/j.biopsych.2014.09.019.	25483401	HAMD: 4.2(4.2)	Negative valence during passive scene viewing	Negative Valence	Potential Threat ("Anxiety")	Negative stimuli vs baseline: Recurrent MDD Patients > Healthy controls
Arnone	2012	Increased amygdala responses to sad but not fearful faces in major depression: relation to	Am J Psychiatry. 2012 Aug;169(8):841-50. doi: 10.1176/appi.ajp.2012.11121774.	22854930	MADRS: 27(4.2)	Negative valence during facial affect processing	Negative Valence	Potential Threat ("Anxiety")	Fear vs neutral, sad vs neutral: Currently depressed patients > Healthy controls; Patients < Healthy controls

© 2019 Janiri D et al. *JAMA Psychiatry.*

		mood state and pharmacological treatment.				Positive valence during facial affect processing	Positive Valence	Approach Motivation	Happy vs neutral: Currently depressed Patients > Healthy controls
Arrondo	2015	Reduction in ventral striatal activity when anticipating a reward in depression and schizophrenia: a replicated cross-diagnostic finding.	Front Psychol. 2015 Aug 26;6:1280. doi: 10.3389/fpsyg.2015.01280.	26379600	BDI: 32(NA)	Monetary incentive delay task	Positive Valence	Approach Motivation	Reward anticipation vs anticipation of a neutral outcome: D patients < Healthy controls
Aust	2013	Music in depression: Neural correlates of emotional experience in remitted depression	World J Psychiatr. 2013 June 22; 3(2):8 -17. doi: 10.5498/wjp.v3.i2.	24175181	HAMD: 3.8(1.5)	Positive valence during passive scene viewing and auditory stimuli	Positive Valence	Approach Motivation	Pleasant stimuli vs neutral: Remitted depressed patients > Healthy controls; Remitted depressed patients < Healthy controls
						Negative valence during passive scene viewing and auditory stimuli	Negative Valence	Potential Threat ("Anxiety")	Unpleasant stimuli: Remitted depressed patientss > Healthy controls; Remitted depressed patients < Healthy controls
Bermpohl	2009	Attentional modulation of emotional stimulus processing in patients with major depression—Alterations in	Neurosci Lett. 2009 Oct 2;463(2):108-13. doi: 10.1016/j.neulet.2009.07.061.	19632301	HAMD: 24.7(3.6)	Unspecified valence during passive scene viewing	Cross-domain affective	Cross-domain affective	Emotional vs neutral expectancy: Depressed patients > Healthy controls; Depressed patients < Healthy controls

© 2019 Janiri D et al. *JAMA Psychiatry.*

		prefrontal cortical regions							
Briceno	2013	Shifted Inferior Frontal Laterality in Women with Major Depressive Disorder is Related to Emotion Processing Deficits	Psychol Med. 2013 July ; 43(7): 1433–1445. doi: 10.1017/S0033291712002176.	23298715	HAMD: 15.8(7.2)	Unspecified valence during facial affect processing	Social Process	Social Communication	Emotion discrimination vs animal discrimination: MDD patients > Healthy controls; MDD patients < Healthy controls
Chandrasekhar Pammi	2015	Neural loss aversion differences between depression patients and healthy individuals: A functional MRI investigation.	Neuroradiol J. 2015 Apr;28(2):97-105. doi: 10.1177/1971400915576670	25923684	N.A.	Probabilistic reward task	Positive Valence	Initial Responsiveness to Reward Attainment	Gain vs loss: MDD patients > Healthy controls; MDD patients < Healthy controls
Chase	2013	Dissociable patterns of abnormal frontal cortical activation during anticipation of an uncertainreward or loss in bipolar versus major depression.	Bipolar Disord. 2013 Dec;15(8):839-854. doi: 10.1111/bdi.12132.	24148027	HAMD: 26.63(5.7)	Card-guessing task	Positive Valence	Approach Motivation	Reward expectancy vs baseline: MDD patients < Healthy controls
Demenescu	2011	Neural correlates of perception of emotional facial expressions in out-patients with mild-to-moderate depression and anxiety. A	Psychol Med. 2011 Nov;41(11):2253-64. doi: 10.1017/S0033291711000596.	21557888	MADRS: 11.16(8.66)	Positive valence during facial affect processing	Positive Valence	Approach Motivation	Happy vs scrambled: MDD patients > Healthy controls
						Unspecified valence	Social Process	Social Communication	Neutral vs scrambled: MDD patients > Healthy controls

© 2019 Janiri D et al. *JAMA Psychiatry.*

		multicenter fMRI study.				during facial affect processing			
Derntl	2011	Neural correlates of social approach and withdrawal in patients with major depression	Soc Neurosci. 2011;6(5-6):482-501. doi: 10.1080/17470919.2011.579800.	21777105	HAMD: 19.9(7.3)	Positive valence during facial affect processing	Positive Valence	Approach Motivation	Happy vs neutral: Patients > Healthy controls; Patients < Healthy controls
						Negative valence during facial affect processing	Negative Valence	Potential Threat ("Anxiety")	Anger vs neutral: Patients < Healthy controls
Dichter	2012	Remitted major depression is characterized by reward network hyperactivation during reward anticipation and hypoactivation during reward outcomes	J Affect Disord. 2012 February ; 136(3): 1126–1134. doi: 10.1016/j.jad.2011.09.048.	22036801	BDI: 2.63(4.91)	Monetary incentive delay task	Positive Valence	Approach Motivation	Reward anticipation (potential win vs non-potential win): Remitted MDD Patients > Healthy controls;
							Positive Valence	Initial Responsiveness to Reward Attainment	Reward outcome (wins vs non-wins): Remitted MDD Patients > Healthy controls; Remitted MDD Patients < Healthy controls
Epstein	2006	Lack of ventral striatal response to positive stimuli in depressed versus normal subjects.	Am J Psychiatry. 2006 Oct;163(10):1784-90. DOI: 10.1176/ajp.2006.163.10.1784	17012690	NA	Positive valence during emotion word processing	Positive Valence	Approach Motivation	Positive vs Neutral: Depressed patients < Healthy controls
						Negative valence during emotion	Negative Valence	Potential Threat	Negative vs Neutral: Depressed patients > Healthy controls;

© 2019 Janiri D et al. *JAMA Psychiatry.*

						word processing		("Anxiety")	Depressed patients < Healthy controls
Fournier	2013	Heterogeneity of amygdala response in major depressive disorder: the impact of lifetime subthreshold mania.	Psychol Med. 2013 Feb;43(2):293-302. doi: 10.1017/S0033291712000918.	22571805	HAMD: 21.4(3.9)	Unspecified valence during facial affect processing	Social Process	Social Communication	All four emotion vs shapes: MDD patients > Healthy controls
Frodl	2009	Neuronal correlates of emotional processing in patients with major depression	World J Biol Psychiatry. 2009;10(3):202-8. doi: 10.1080/15622970701624603	17965984	HAMD: 17.5(4.4)	Negative valence during facial affect processing	Negative Valence	Potential Threat ("Anxiety")	Emotional processing vs match forms: Patients > Healthy controls
Fu	2007	Neural responses to happy facial expressions in major depression following antidepressant treatment.	Am J Psychiatry. 2007 Apr;164(4):599-607. DOI: 10.1176/ajp.2007.164.4.599	17403973	HAMD: 21.1(2.3)	Positive valence during facial affect processing	Positive Valence	Approach Motivation	Happy Faces vs baseline: Depressed patients < Healthy controls
Fu	2008	Neural Responses to Sad Facial Expressions in Major Depression Following Cognitive Behavioral Therapy	Biol Psychiatry. 2008 Sep 15;64(6):505-12. doi: 10.1016/j.biopsych.2008.04.033. Epub 2008 Jun 12.	18550030	HAMD: 20.9(1.9)	Negative valence during facial affect processing	Negative Valence	Potential Threat ("Anxiety")	Pre-treatment, Sad facial affect vs baseline : Patients < Healthy controls
Gotlib	2005	Subgenual anterior cingulate activation to	Neuroreport. 2005 Nov 7;16(16):1731-4.	16237317	BDI-II: 24.6(8.3)	Positive valence during facial	Positive Valence	Approach Motivation	Happy vs neutral faces: MDD patients > Healthy controls; MDD patients < Healthy controls

© 2019 Janiri D et al. *JAMA Psychiatry.*

		valenced emotional stimuli in major depression				affect processing			
						Negative valence during facial affect processing	Negative Valence	Potential Threat ("Anxiety")	Sad vs neutral faces: MDD patients > Healthy controls; MDD patients < Healthy controls
Greening	2013_b	Emotion-related brain activity to conflicting socio-emotionalcues in unmedicated depression	J Affect Disord. 2013 Sep 25;150(3):1136-41. doi: 10.1016/j.jad.2013.05.053.	23769293	BDI: 25.53(10.4)	Negative valence during facial affect processing	Negative Valence	Potential Threat ("Anxiety")	Response to fearful task-irrelevant distracters vs baseline: Patients < Healthy controls
Grimm	2008	Imbalance between Left and Right Dorsolateral Prefrontal Cortex in Major Depression Is Linked to Negative Emotional Judgment: An fMRI Study in Severe Major Depressive Disorder	Biol Psychiatry. 2008 Feb 15;63(4):369-76. doi: 10.1016/j.biopsych.2007.05.033	17888408	HAMD: 33.12(7.13)	Unspecified valence during passive scene viewing	Cross-domain affective	Cross-domain affective	Emotional judgment vs passive viewing: Patients > Healthy controls; Patients < Healthy controls
Grimm	2009	Altered negative BOLD responses in the default-mode network during emotion processing in depressed subjects.	Neuropsychopharmacology. 2009 Mar;34(4):932-43. doi: 10.1038/npp.2008.81.	18536699	HAMD: 33.1(7.1)	Unspecified valence during passive scene viewing	Cross-domain affective	Cross-domain affective	Emotion perception vs baseline: MDD Patients > Healthy controls; MDD Patients < Healthy controls

© 2019 Janiri D et al. *JAMA Psychiatry.*

Hao	2015	Neural correlates of causal attribution in negative events of depressed patients: Evidence from an fMRI study.	Clin Neurophysiol. 2015 Jul;126(7):1331-7. doi: 10.1016/j.clinph.2014.10.146.	25487911	HAMD: 20.3(7.8)	Self-referential processing task	Social Process	Perception and Understanding of Self (Self)	Non self-serving vs self-serving: Depressed patients > Healthy controls
Johnston	2015	Failure of hippocampal deactivation during loss events in treatment-resistant depression.	Brain. 2015 Sep;138(Pt 9):2766-76. doi: 10.1093/brain/awv177.	26133661	HAMD: 21(2.5)	Probabilistic reward task	Positive Valence	Initial Responsiveness to Reward Attainment	win vs control: MDD Patients > Healthy controls; MDD Patients < Healthy controls
							Negative Valence	Frustrative Nonreward	loss vs control: MDD Patients > Healthy controls; MDD Patients < Healthy controls
Keedwell	2005	A double dissociation of ventromedial prefrontal cortical responses to sad and happy stimuli in depressed and healthy individuals.	Biol Psychiatry. 2005 Sep 15;58(6):495-503. DOI: 10.1016/j.biopsych.2005.04.035	15993859	BDI: 33.5(11.2)	Positive valence during facial affect processing	Positive Valence	Approach Motivation	Happy Mood vs neutral conditions: Depressed patients > Healthy controls; Depressed patients < Healthy controls
						Negative valence during facial affect processing	Negative Valence	Potential Threat ("Anxiety")	Sad Mood vs neutral condition: Depressed patients > Healthy controls; Depressed patients < Healthy controls
Mingtian	2012	Elevated amygdala activity to negative faces in young adults with early onset major depressivedisorder.	Psychiatry Res. 2012 Feb 28;201(2):107-12. doi: 10.1016/j.pscychresns.2011.06.003.	22398297	CES-D: 25.11(5.42)	Negative valence during facial affect processing	Negative Valence	Potential Threat ("Anxiety")	Matching fearful and angry faces vs matching forms: MDD patients > Healthy controls; MDD patients < Healthy controls

© 2019 Janiri D et al. *JAMA Psychiatry.*

Murrough	2015	Regulation of neural responses to emotion perception by ketamine in individuals with treatment-resistant major depressive disorder.	Transl Psychiatry. 2015 Feb 17;5:e509. doi: 10.1038/tp.2015.10.	25689570	MADRS: 29.9(6.8)	Positive valence during facial affect processing	Positive Valence	Approach Motivation	Baseline, Happy 100% vs neutral: Treatment resistant depressed patients < Healthy controls
Norbury	2010	Increased neural response to fear in patients recovered from depression: a 3T functional magnetic resonance imaging study.	Psychol Med. 2010 Mar;40(3):425-32. doi: 10.1017/S0033291709990596.	19627640	BDI: 3.5(3.7)	Unspecified valence during facial affect processing	Social Process	Social Communication	Fearful vs happy facial expressions: Recovered depressed patients > Healthy controls
Pizzagalli	2009	Reduced caudate and nucleus accumbens response to rewards in unmedicated individuals with major depressive disorder.	Am J Psychiatry. 2009 Jun;166(6):702-10. doi: 10.1176/appi.ajp.2008.08081201.	19411368	HAMD: 17.97(4.9)	Monetary incentive delay task	Positive Valence	Approach Motivation	Reward cue vs No incentive cue: Patients > Healthy controls; Patients < Healthy controls
							Positive Valence	Initial Responsiveness to Reward Attainment	Gain vs No-change feedback: Patients > Healthy controls; Patients < Healthy controls
							Negative valenced	Potential Threat ("Anxiety")	Loss cue vs No incentive cue: Patients > Healthy controls; Patients < Healthy controls
							Negative valenced	Frustrative Nonreward	Penalty vs No-change feedback: Patients > Healthy controls; Patients < Healthy controls
Regenbogen	2015	Neural responses to dynamic multimodal stimuli and pathol	Br J Psychiatry. 2015 Mar;206(3):198-205. doi:	25573396	HAMD: 14.45(6.67)	Unspecified valence during	Social Process	Social Communication	Trimodal emotional vs trimodal neutral: Depressed patients > Healthy controls;

© 2019 Janiri D et al. *JAMA Psychiatry.*

		ogy-specific impairments of social cognition in schizophrenia and depression.	10.1192/bjp.bp.113.143040.			facial affect processing			Depressed patients < Healthy controls
Remijnse	2009	Differential frontal-striatal and paralimbic activity during reversal learning in major depressive disorder and obsessive-compulsive disorder.	Psychol Med. 2009 Sep;39(9):1503-18. doi: 10.1017/S0033291708005072.	19171077	HAMD: 19.1(4.1)	Reversal learning task	Positive Valence	Initial Responsiveness to Reward Attainment	Reward (correct responses vs baseline): MDD patients > Healthy controls
							Negative valenced	Frustrative Nonreward	Punishment (probabilistic errors no shift+final reversal errors+preceding reversal errors vs baseline): MDD patients > Healthy controls; Patients < Healthy controls
Ritchey	2011	Neural correlates of emotional processing in depression: changes with cognitive behavioraltherapy and predictors of treatment response.	J Psychiatr Res. 2011 May;45(5):577-87. doi: 10.1016/j.jpsychires.2010.09.007.	20934190	HAMD: 26.7(6.7)	Unspecified valence during passive scene viewing	Cross-domain affective	Cross-domain affective	Pre-Treatment, Negative & Positive vs Neutral: Patients < Healthy controls
Rizvi	2013	Neural response to emotional stimuli associated with successful antidepressant treatment and behavioral activation.	J Affect Disord. 2013 Nov;151(2):573-81. doi: 10.1016/j.jad.2013.06.050.	23948629	HAMD: 21.8(1.5)	Positive valence during passive scene viewing	Positive Valence	Approach Motivation	Pre-treatment, positive images vs neutral: MDD patients > Healthy controls
						Negative valence during passive scene viewing	Negative Valence	Potential Threat ("Anxiety")	Pre-treatment, negative images vs neutral: MDD Patients > Healthy controls

© 2019 Janiri D et al. *JAMA Psychiatry.*

Robinson	2012	Ventral striatum response during reward and punishment reversal learning in unmedicated majordepressive disorder.	Am J Psychiatry. 2012 Feb;169(2):152-9. DOI: 10.1176/appi.ajp.2011.11010137	22420038	HAMD: 20(7)	Reversal learning task	Positive Valence	Initial Responsiveness to Reward Attainment	Unexpected reward vs baseline: Depressed patients < Healthy controls
Sarsam	2013	The Queen and I: neural correlates of altered self-related cognitions in major depressive episode.	PLoS One. 2013 Oct 30;8(10):e78844. doi: 10.1371/journal.pone.0078844.	24205330	BDI-II: 29.1(12.9)	Self-referential processing task	Social Process	Perception and Understanding of Self (Self)	Self vs queen: Depressed patients > Healthy controls
Scheuerecker	2010	Orbitofrontal volume reductions during emotion recognition in patients with major depression.	J Psychiatry Neurosci. 2010 Sep;35(5):311-20. doi: 10.1503/jpn.090076.	20569645	HAMD: 20.5(4.7)	Negative valence during facial affect processing	Negative Valence	Potential Threat ("Anxiety")	Sad or angry faces vs shapes: Patients > Healthy controls
Segarra	2016	Abnormal Frontostriatal Activity During Unexpected Reward Receipt in Depression and Schizophrenia: Relationship to Anhedonia.	Neuropsychopharmacology. 2016 Jul;41(8):2001-10. doi: 10.1038/npp.2015.370.	26708106	HAMD: 3.64(2.63)	Probabilistic reward task	Positive Valence	Initial Responsiveness to Reward Attainment	Win outcomes vs full miss outcomes: Depressed patients < Healthy controls
Shi	2015	Default mode network alterations during implicit emotional faces processing in first-episode, treatment-naive major depression patients.	Front Psychol. 2015 Aug 12;6:1198. doi: 10.3389/fpsyg.2015.01198.	26322003	CES-D: 58.1(5.73)	Unspecified valence during facial affect processing	Social Process	Social Communication	Emotional faces vs baseline: MDD patients > Healthy controls

© 2019 Janiri D et al. *JAMA Psychiatry.*

Smoski	2009	fMRI of alterations in reward selection, anticipation, and feedback in major depressive disorder.	J Affect Disord. 2009 Nov;118(1-3):69-78. doi: 10.1016/j.jad.2009.01.034.	19261334	HAMD: 23.5 (n.a.)	Probabilistic reward task	Positive Valence	Approach Motivation	Anticipation phase (Money vs control trials): MDD patients > Healthy controls; MDD patients < Healthy controls
							Positive Valence	Initial Responsiveness to Reward Attainment	Feedback phase (Winning vs Control trials): MDD patients > Healthy controls; MDD patients < Healthy controls
							Negative valenced	Frustrative Nonreward	Feedback phase (Non-win vs Control trials): MDD patients > Healthy controls; MDD patients < Healthy controls
Smoski	2011	Major depressive disorder is characterized by greater reward network activation to monetary than pleasant image rewards.	Psychiatry Res. 2011 Dec 30;194(3):263-270. doi: 10.1016/j.pscychresns.2011.06.012.	22079658	BDI: 16.7(4.9)	Monetary incentive delay task	Positive Valence	Approach Motivation	Anticipation phase, Money and Images (potential win vs non potential win): MDD patients < Healthy controls
							Positive Valence	Initial Responsiveness to Reward Attainment	Reward outcome, Money and Images (win vs non-win):MDD patients < Healthy controls
Surguladze	2005	A differential pattern of neural response toward sad versus happy facial expressions in major depressive disorder.	Biol Psychiatry. 2005 Feb 1;57(3):201-9. DOI: 10.1016/j.biopsych.2004.10.028	15691520	BDI: 31.1(10.8)	Positive valence during facial affect processing	Positive Valence	Approach Motivation	Response to Increasing Intensities to Happy Facial Expressions vs baseline: Patients < Healthy controls
						Negative valence during facial affect	Negative Valence	Potential Threat ("Anxiety")	Response to Increasing Intensities to Sad Facial expressions vs baseline: Patients > Healthy controls

© 2019 Janiri D et al. *JAMA Psychiatry.*

						processing			
Surguladze	2010	Depression is associated with increased sensitivity to signals of disgust: a functional magnetic resonance imaging study.	J Psychiatr Res. 2010 Oct;44(14):894-902. doi: 10.1016/j.jpsychires.2010.02.010.	20307892	HAMD: 17.7(5.5)	Negative valence during facial affect processing	Negative Valence	Potential Threat ("Anxiety")	Disgust and fear vs neutral faces: Patients > Healthy controls; Patients < Healthy controls
Townsend	2010	fMRI activation in the amygdala and the orbitofrontal cortex in unmedicated subjects with major depressive disorder	Psychiatry Res. 2010 Sep 30;183(3):209-17. doi: 10.1016/j.pscychresns.2010.06.001.	20708906	HAMD: 20.1(4.9)	Negative valence during facial affect processing	Negative Valence	Potential Threat ("Anxiety")	Match fearful or sad faces vs match forms: Depressed patients < Healthy controls
Tozzi	2016	Single-Nucleotide Polymorphism of the FKBP5 Gene and Childhood Maltreatment as Predictor sof Structural Changes in Brain Areas Involved in Emotional Processing in Depression.	Neuropsychopharmacology. 2016 Jan;41(2):487-97. doi: 10.1038/npp.2015.170.	26076833	HAMD: 28.4(7)	Unspecified valence during passive scene viewing	Cross-domain affective	Cross-domain affective	Emotional vs geometrical: Patients < Healthy controls
Tremblay	2005	Functional neuroanatomical substrates of altered reward processing in major depressivedisorder revealed by	Arch Gen Psychiatry. 2005 Nov;62(11):1228-36. DOI: 10.1001/archpsyc.62.11.1228	16275810	HAMD: 27.75(3.05)	Unspecified valence during passive scene viewing	Cross-domain affective	Cross-domain affective	All pictures vs fixation crosses: MDD patients > Healthy controls; MDD patients < Healthy controls

© 2019 Janiri D et al. *JAMA Psychiatry.*

		a dopaminergic probe.							
van Wingen	2011	Neural basis of emotion recognition deficits in first-episode major depression.	Psychol Med. 2011 Jul;41(7):1397-405. doi: 10.1017/S0033291710002084.	21054920	HAMD: 21.8(4.2)	Negative valence during facial affect processing	Negative Valence	Potential Threat ("Anxiety")	Emotion labelling and matching (angry or fearful faces) vs control condition: Depressed patients > Healthy controls
Victor	2010	Relationship between amygdala responses to masked faces and mood state and treatment in major depressive disorder.	Arch Gen Psychiatry. 2010 Nov;67(11):1128-38. doi: 10.1001/archgenpsychiatry.2010.144.	21041614	HAMD: 24(6.3)	Unspecified valence during facial affect processing	Social Process	Social Communication	Unmasked-sad vs unmasked-happy faces: Patients > Healthy controls; Patients < Healthy controls
Victor	2012	The extended functional neuroanatomy of emotional processing biases for masked faces in major depressive disorder.	PLoS One. 2012;7(10):e46439. doi: 10.1371/journal.pone.0046439.	23056309	HAMD: 24(6.3)	Positive valence during facial affect processing	Positive Valence	Approach Motivation	Masked-happy faces vs masked-neutral faces: Patients > Healthy controls; Patients < Healthy controls
						Negative valence during facial affect processing	Negative Valence	Potential Threat ("Anxiety")	Masked-sad vs masked-neutral: Patients > Healthy controls; Patients < Healthy controls
Wagner	2015	The neural basis of the abnormal self-referential processing and its impact on cognitive control in depressed patients.	Hum Brain Mapp. 2015 Jul;36(7):2781-94. doi: 10.1002/hbm.22807.	25872899	HAMD: 23.1(4.6)	Self-referential processing task	Social Process	Perception and Understanding of Self (Self)	Negative self-referential processing vs baseline: Patients < Healthy controls

© 2019 Janiri D et al. *JAMA Psychiatry.*

Wang	2012	Effects of an antidepressant on neural correlates of emotional processing in patients with major depression.	Neurosci Lett. 2012 Oct 3;527(1):55-9. doi: 10.1016/j.neulet.2012.08.034.	22954751	n.a.	Positive valence during passive scene viewing	Positive Valence	Approach Motivation	Pre-treatment, Positive stimuli vs baseline: Patients < Healthy controls
						Negative valence during passive scene viewing	Negative Valence	Potential Threat ("Anxiety")	Pre-treatment,Negative stimuli vs baseline: Patients > Healthy controls
Whalley	2012	Autobiographical memory in depression: an fMRI study.	Psychiatry Res. 2012 Feb 28;201(2):98-106. doi: 10.1016/j.pscychresns.2011.08.008.	22386970	BDI - II: 29.53(7.87)	Self-referential memory paradigm	Social Process	Perception and Understanding of Self (Self)	OwnHit vs elseHit (combination of words and sentences tasks): Patients < Healthy controls
Yang	2016	Diminished caudate and superior temporal gyrus responses to effort-based decision making in patients with first-episode major depressive disorder.	Prog Neuropsychopharmacol Biol Psychiatry. 2016 Jan 4;64:52-9. doi: 10.1016/j.pnpbp.2015.07.006.	26192817	HAMD: 27.58(4.62)	Probabilistic reward task	Positive Valence	Approach Motivation	Prob8 vs rest: Depressed patients < Healthy controls
							Positive Valence	Initial Responsiveness to Reward Attainment	High reward vs rest: Depressed patients < Healthy controls
Young	2012	Functional anatomy of autobiographical memory recall deficits in depression.	Psychol Med. 2012 Feb;42(2):345-57. doi: 10.1017/S0033291711001371.	21798113	HAMD: 21(8.3)	Self-referential memory paradigm	Social Process	Perception and Understanding of Self (Self)	Any memory vs substraction: Patients < Healthy controls
Zhong	2011	Amygdala hyperactivation and prefrontal hypoactivation in subjects with cognitive vulner	Biol Psychol. 2011 Dec;88(2-3):233-42. doi: 10.1016/j.biopsycho.2011.08.007.	21878364	CES-D: 34.86(5.41)	Negative valence during facial affect processing	Negative Valence	Potential Threat ("Anxiety")	Matching fearful or angry faces vs matching forms: Patients > Healthy controls; Patients < Healthy controls

© 2019 Janiri D et al. *JAMA Psychiatry.*

		abilityto depression.							
**Studies using experiments mapping the RDoC cognitive systems domain**									
Backes	2014	Increased neural activity during overt and continuous semantic verbal fluency in major depression: mainly a failure to deactivate.	Eur Arch Psychiatry Clin Neurosci. 2014 Oct;264(7):631-45. doi: 10.1007/s00406-014-0491-y.	24557502	BDI: 27.27(8.27)	Verbal fluency task	Cognitive System	Language	Word generation vs baseline: MDD Patients > Healthy controls
Dietsche	2014	Altered neural function during episodic memory encoding and retrieval in major depression.	Hum Brain Mapp. 2014 Sep;35(9):4293-302. doi: 10.1002/hbm.22475.	24639328	HAMD: 17.75(3.64)	Episodic memory encoding /retrieval task	Cognitive System	Declarative Memory	Encoding and recognition vs baseline: Patients > Healthy controls; Patients < Healthy controls
Fitzgerald	2008	An fMRI study of prefrontal brain activation during multiple tasks in patients with major depressive disorder.	Hum Brain Mapp. 2008 Apr;29(4):490-501. DOI: 10.1002/hbm.20414	17525987	MADRS: 32.7(11.9)	Tower of London	Cognitive System	Cognitive control	Planning condition vs rest: Patients > Healthy controls
						N-back task	Cognitive System	Working memory	2-back vs 0-back: Patients > Healthy controls
Garrett	2011	Aberrant brain activation during a working memory task in psychotic major depression.	Am J Psychiatry. 2011 Feb;168(2):173-82. doi: 10.1176/appi.ajp.2010.09121718.	21078708	HAMD: 24.2(3.3)	N-back task	Cognitive System	Working memory	2-back task vs press for Z: NPMD patients> Healthy controls; NPMD patients < Healthy controls

© 2019 Janiri D et al. *JAMA Psychiatry*.

Harvey	2005	Cognitive control and brain resources in major depressi on: an fMRI study using the n-back task.	Neuroimage. 2005 Jul 1;26(3):860-9. doi: 10.1016/j.neuroima ge.2005.02.048	15955496	MADRS: 26.7(4.6)	N-back task	Cogn itive Syste m	Working memory	Nback (1-2-3-back) vs 0-back: Depressed patients > Healthy controls
Hugdahl	2007	Increased Parietal and Frontal Activation after Remission from Recurrent Major Depression: A Repeated fMRI Study	Cogn Ther Res. 2007 Mar; 31:147–160 DOI 10.1007/s10608-006-9116-8		MADRS: 26.7(4.6)	Mental Arithmeti c Task	Cogn itive Syste m	Unclassified	Scan I, Mental arithmetic task vs vigilance task: Patients < Healthy controls
Kassel	2016	Decreased Fronto-Limbic Activation and Disrupted Semantic-Cued List Learning in Major Depressi ve Disorder.	J Int Neuropsychol Soc. 2016 Apr;22(4):412-25. doi: 10.1017/S1355617 716000023.	26831638	HAMD: 15.1(6.4)	Semanti c list learning task	Cogn itive Syste m	Declarative Memory	Encoding vs silent rehearsal and words recalled vs baseline: MDD patients < Healthy controls
Langenecke r	2007	Frontal and limbic activation during inhibitory control predicts treatment response in major depressi ve disorder.	Biol Psychiatry. 2007 Dec 1;62(11):1272-80. doi: 10.1016/j.neuroima ge.2005.02.048	17585888	HAMD: 20.4(7.6)	Go/no-go task	Cogn itive Syste m	Cognitive control	Activation for Rejections vs baseline: MDD patients > Healthy controls; MDD patients < Healthy controls
Matsuo	2007	Prefrontal hyperactivation during working memory task in untreated individuals with	Mol Psychiatry. 2007 Feb;12(2):158-66. doi: 10.1038/sj.mp.400 1894	16983390	HAMD: 20.3(5.3)	N-back task	Cogn itive Syste m	Working memory	2-back vs 1-back: MDD patients > Healthy controls

© 2019 Janiri D et al. *JAMA Psychiatry.*

		major depressive disorder.							
Matthews	2009	Inhibition-related activity in subgenual cingulate is associated with symptom severity in majordepression.	Psychiatry Res. 2009 Apr 30;172(1):1-6. doi: 10.1016/j.pscychresns.2008.08.006.	19239982	BDI-II range: 15-43	Stop task (Go/no-go task)	Cognitive System	Cognitive control	Inhibitory processing (hard vs easy trials): MDD patients > Healthy controls
Naismith	2010	Fronto-striatal correlates of impaired implicit sequence learning in major depression: an fMRIstudy.	J Affect Disord. 2010 Sep;125(1-3):256-61. doi: 10.1016/j.jad.2010.02.114.	20219248	HAMD: 21.6(4.2)	Implicit learning task	Cognitive System	Unclassified	Implicit learning vs baseline condition: MDD patients > Healthy controls; MDD patients < Healthy controls
Remijnse	2013	Cognitive inflexibility in obsessive-compulsive disorder and major depression is associated with distinct neural correlates.	PLoS One. 2013 Apr 24;8(4):e59600. doi: 10.1371/journal.pone.0059600.	23637737	HAMD: 20.1(4.4)	Task switching paradigm	Cognitive System	Cognitive control	Switch vs repeat trials: Patients < Healthy controls
Schöning	2009	Working-memory fMRI reveals cingulate hyperactivation in euthymic major depression.	Hum Brain Mapp. 2009 Sep;30(9):2746-56. doi: 10.1002/hbm.20702.	19086021	HAMD: 3.64(2.63)	N-back task	Cognitive System	Working memory	2-back vs 0-back: Patients > Healthy controls
Walsh	2007	A longitudinal functional magnetic resonance imaging study of verbal working memory	Biol Psychiatry. 2007 Dec 1;62(11):1236-43. doi: 10.1016/j.biopsych.2006.12.022	17601497	HAMD: 21.2(2.4)	N-back task	Cognitive System	Working memory	1-2-3-back vs 0-back: Depressed patients < Healthy controls

© 2019 Janiri D et al. *JAMA Psychiatry.*

		in depression after antidepressant therapy.							
Walter	2007	Increased left prefrontal activation in patients with unipolar depression: an event-related, parametric, performance-controlled fMRI study.	J Affect Disord. 2007 Aug;101(1-3):175-85. doi: 10.1016/j.jad.2006.11.017	17197035	HAMD: 18.2(3.7)	Modified sternberg task	Cognitive System	Working memory	All loads vs control: Patients > Healthy controls; Patients < Healthy controls
Werner	2009	Functional MRI study of memory-related brain regions in patients with depressive disorder.	J Affect Disord. 2009 Dec;119(1-3):124-31. doi: 10.1016/j.jad.2009.03.003.	19346000	BDI: 20.27(8.74)	Paired associates learning task	Cognitive System	Declarative Memory	Encoding and retrieval vs head templates: Patients > Healthy controls; Patients < Healthy controls
Rao	2015	The double burden of age and major depressive disorder on the cognitive control network.	Psychol Aging. 2015 Jun;30(2):475-85. doi: 10.1037/pag0000027.	26030776	HAMD: 15(NA)	Go/no-go task	Cognitive System	Cognitive control	Correct hits vs baseline: Younger MDD patients >Younger healthy controls
Rodriguez-Cano	2014	Evidence for structural and functional abnormality in the subgenual anterior cingulate cortex in major depressive disorder.	Psychol Med. 2014 Nov;44(15):3263-73. doi: 10.1017/S0033291714000841.	25066663	HAMD: 26.84(4.48)	N-back task	Cognitive System	Working memory	2-back versus baseline: depressed patients < Healthy controls
Rose	2006	Limbic over-activity in depression during preserved performance on	Neuroimage. 2006 Jan 1;29(1):203-15.DOI:	16157491	HAMD: 20.56(5.59)	N-back task	Cognitive System	Working memory	Linear increase in the level of difficulty (3-2-1-back vs 0-back): Patients > Healthy controls

© 2019 Janiri D et al. *JAMA Psychiatry.*

		the n-back task.	10.1016/j.neuroimage.2005.07.002						
Takamura	2016	Disrupted Brain Activation and Deactivation Pattern during Semantic Verbal Fluency Task in Patients with Major Depression.	Neuropsychobiology. 2016;74(2):69-77. doi: 10.1159/000453399	28052303	HAMD: 20.7(5.5)	Verbal fluency task	Cognitive System	Language	T1, Semantic verbal fluency task vs baseline: Patients > Healthy controls; Patients < Healthy controls
**Studies including cross-domain experiments**									
Cerullo	2014	Bipolar I disorder and major depressive disorder show similar brain activation during depression.	Bipolar Disord. 2014 Nov;16(7):703-12. doi: 10.1111/bdi.12225.	24990479	HAMD: 32(7)	Emotional visual oddball task	Cognitive-affective interaction	Cognitive-affective interaction	Emotional images, circles vs square trials: MDD patients > Healthy controls; MDD patients < Healthy controls
Chechko	2013	Brain circuitries involved in emotional interference task in major depression disorder.	J Affect Disord. 2013 Jul;149(1-3):136-45. doi: 10.1016/j.jad.2013.01.013.	23394712	HAMD: 22.7(5)	Emotional Stroop task	Cognitive-affective interaction	Cognitive-affective interaction	Incongruent vs congruent in emotional interference: Patients < Healthy controls
Dichter	2009	Affective context interferes with cognitive control in unipolar depression: an fMRI investigation.	J Affect Disord. 2009 Apr;114(1-3):131-42. doi: 10.1016/j.jad.2008.06.027.	18706701	BDI: 26.9(4.9)	Emotional visual oddball task	Cognitive-affective interaction	Cognitive-affective interaction	Targets within sad blocks vs baseline: MDD patients < Healthy controls
Greening	2013_a	The neural correlates of regulating positive and negative	Soc Cogn Affect Neurosci. 2014 May;9(5):628-37. doi:	23482626	BDI: 24.56(9.8)	Emotion regulation task	Cognitive-affective	Cognitive-affective interaction	Reduce vs attend: MDD Patients > Healthy controls

© 2019 Janiri D et al. *JAMA Psychiatry.*

		emotions in medication-free major depression	10.1093/scan/nst027.				interaction		
Heller	2009	Reduced capacity to sustain positive emotion in major depression reflects diminished maintenance of fronto-striatal brain activation.	Proc Natl Acad Sci U S A. 2009 Dec 29;106(52):22445-50. doi: 10.1073/pnas.0910651106.	20080793	HAMD: 20.6(2.39)	Emotion regulation task	Cognitive-affective interaction	Cognitive-affective interaction	Enhance vs suppress condition: Depressed patients < Healthy controls
Johnstone	2007	Failure to regulate: counterproductive recruitment of top-down prefrontal-subcortical circuitry in major depression.	J Neurosci. 2007 Aug 15;27(33):8877-84. doi:10.1523/JNEUROSCI.2063-07.2007	17699669	HAMD: 21(2.5)	Emotion regulation task	Cognitive-affective interaction	Cognitive-affective interaction	Decrease condition vs attend condition: Patients > Healthy controls
Lisiecka	2013	Recruitment of the left hemispheric emotional attention neural network in risk for and protectionfrom depression.	J Psychiatry Neurosci. 2013 Mar;38(2):117-28. doi: 10.1503/jpn.110188.	23010257	HAMD: 28.1(6.6)	Emotional attention shifiting task	Cognitive-affective interaction	Cognitive-affective interaction	Emotion processing, attention shifting vs baseline: MDD-FHN>HC-FHN; MDD-FHN>HC-FHN
Mitterschiffthaler	2008	Neural basis of the emotional Stroop interference effect in major depression.	Psychol Med. 2008 Feb;38(2):247-56. DOI: 10.1017/S0033291707001523	17825123	HAMD: 20.88(1.83)	Emotional Stroop task	Cognitive-affective interaction	Cognitive-affective interaction	Negative words vs. neutral words: MDD patients > Healthy controls

© 2019 Janiri D et al. *JAMA Psychiatry.*

Smoski	2015	Neural indicators of emotion regulation via acceptance vs reappraisal in remitted major depressive disorder.	Soc Cogn Affect Neurosci. 2015 Sep;10(9):1187-94. doi: 10.1093/scan/nsv003.	25617820	BDI: 2.9(5)	Emotion regulation task	Cognitive-affective interaction	Cognitive-affective interaction	Acceptance vs reappraising: rMDD patients < Healthy controls
Wang	2008	Prefrontal mechanisms for executive control over emotional distraction are altered in majordepression.	Psychiatry Res. 2008 Jul 15;163(2):143-55. doi: 10.1016/j.pscychresns.2007.10.004.	18455373	HAMD: 19.9(5.3)	Emotional visual oddball task	Cognitive-affective interaction	Cognitive-affective interaction	Sad vs Neutral distractors, Targets vs scrambled pictures: MDD patients > Healthy controls

Patients' Symptom severity is provided as mean (standard deviation) when available.

Abbreviations: BDI: Beck Depression Inventory; CES-D: Center for Epidemiologic Studies Depression Scale; HAMD: Hamilton Depression Rating Sale; MADRS: Montgomery-Åsberg Depression Rating Scale.

© 2019 Janiri D et al. *JAMA Psychiatry.*

eTable 4: Bipolar Disorder: Studies included									
**First Author**	**Year**	**Title**	**Publication**	**Patients' Symptom Severity**	**PM ID**	**Task**	**RDoC Domain**	**RDoC Construct**	**Contrasts Used**
**Studies using experiments mapping the RDoC domains of negative valence systems, positive valence systems and social processes**									
Altshuler	2008	Regional brain changes in bipolar I depression: a functional magnetic resonance imaging study.	Bipolar Disord. 2008 Sep;10(6):708-17. doi: 10.1111/j.1399-5618.2008.00617.x.	YMRS: 2.9(1.9); HAMD: 20.8(3.3)	18837865	Negative valence during facial affect processing	Negative Valence	Potential Threat ("Anxiety")	Matches angry or fearful of ferfaces vs shapes: Bipolar depressed patients > Healthy controls; Bipolar depressed patients < Healthy controls
Bermpohl	2010	Altered representation of expected value in the orbitofrontal cortex in mania.	Hum Brain Mapp. 2010 Jul;31(7):958-69. doi: 10.1002/hbm.20909.	YMRS: 18.9(6.2)	19950195	Monetary incentive delay task	Positive Valence	Approach Motivation	Magnitude by valence interaction: Manic patients > Healthy controls
Caseras	2013	Neural mechanisms of cognitive reappraisal of negative self-beliefs in social anxiety disorder.	Biol Psychiatry. 2009 Dec 15;66(12):1091-9. doi: 10.1016/j.biopsych.2009.07.014	YMRS: 1.8(2.8); HAMD: 2.26(2.94)	19717138	Card-guessing task	Positive Valence	Approach Motivation	Reward anticipation vs baseline: Bipolar II patients > Healthy controls
Chase	2013	Dissociable patterns of abnormal frontal cortical activation during anticipation of an uncertainreward or loss in bipolar versus major depression.	Bipolar Disord. 2013 Dec;15(8):839-854. doi: 10.1111/bdi.12132.	YMRS: 4(2.54); HAMD: 24.7(8.02)	24148027	Card-guessing task	Positive Valence	Approach Motivation	Reward expectancy vs baseline: BD patients > Healthy controls

© 2019 Janiri D et al. *JAMA Psychiatry.*

eTable 4: Bipolar Disorder: Studies included									
**First Author**	**Year**	**Title**	**Publication**	**Patients' Symptom Severity**	**PMID**	**Task**	**RDoC Domain**	**RDoC Construct**	**Contrasts Used**
Chen	2006	Explicit and implicit facial affect recognition in manic and depressed States of bipolar disorder: a functional magnetic resonance imaging study.	Biol Psychiatry. 2006 Jan 1;59(1):31-9. doi: 10.1016/j.biopsych.2005.06.008	Manic Patients - YMRS: 24.13(8.27); HAMD: 2(2.98) Depressed Patients - YMRS: 0.43(0.53); HAMD: 18.38(6.44)	16112653	Positive valence during facial affect processing	Positive Valence	Approach Motivation	Response to all intensities of happiness vs neutral faces: Depressed patients > Healthy controls
						Negative valence during facial affect processing	Negative Valence	Potential Threat ("Anxiety")	Response to all intensities of fear vs neutral faces: Manic and depressed patients > Healthy controls;
						Negative valence during facial affect processing	Negative Valence	Potential Threat ("Anxiety")	Response to all intensities of sadness vs neutral faces: Manic patients> Healthy controls
Dima	2016	The polygenic risk for bipolar disorder influences brain regional function relating to visual and default state processing of emotional information.	Neuroimage Clin. 2016 Nov 1;12:838-844. doi: 10.1016/j.nicl.2016.10.022	YMRS: 1.4(3); HAMD-17: 4.8(5.3)	27857885	Negative valence during facial affect processing	Negative Valence	Potential Threat ("Anxiety")	Affect vs neutral faces: Patients > Healthy controls; Patients < Healthy controls

© 2019 Janiri D et al. *JAMA Psychiatry.*

eTable 4: Bipolar Disorder: Studies included									
**First Author**	**Year**	**Title**	**Publication**	**Patients' Symptom Severity**	**PM ID**	**Task**	**RDoC Domain**	**RDoC Construct**	**Contrasts Used**
Dutra	2015	Elevated striatal reactivity across monetary and social rewards in bipolar I disorder.	J Abnorm Psychol. 2015 Nov;124(4):890-904. doi: 10.1037/abn0000092.	YMRS: 1.5(1.72); IDS-C: 3.58(2.08)	26390194	Monetary and social incentive delay task	Positive Valence	Approach Motivation	Reward anticipation (Reward vs Neutral Cue): BD patients < Healthy controls
							Positive Valence	Initial Responsiveness to Reward Attainment	Reward Receipt (Win vsNo Win Outcome): BD patients > Healthy controls
Foland	2008	Evidence for deficient modulation of amygdala response by prefrontal cortex in bipolar mania.	Psychiatry Res. 2008 Jan 15;162(1):27-37. doi: 10.1016/j.pscychresns.2007.04.007	YMRS: 15.1(3.7); HAMD: 9.1(5.3)	18063349	Negative valence during facial affect processing	Negative Valence	Potential Threat ("Anxiety")	Perceive emotion vs control and label emotion vs control: Patients > Healthy controls; Patients < Healthy controls
Foland-Ross	2012	Normal amygdala activation but deficient ventrolateral prefrontal activation in adults with bipolardisorder during euthymia.	Neuroimage. 2012 Jan 2;59(1):738-44. doi: 10.1016/j.neuroimage.2011.07.054.	YMRS: 4.6(2.1); HAMD: 4.6(2.1)	21854858	Unspecified valence during facial affect processing	Social Process	Social Communication	Label emotions vs match forms: Euthymic patients < Healthy controls

© 2019 Janiri D et al. *JAMA Psychiatry.*

eTable 4: Bipolar Disorder: Studies included

First Author	Year	Title	Publication	Patients' Symptom Severity	PMID	Task	RDoC Domain	RDoC Construct	Contrasts Used
Hassel	2008	Elevated striatal and decreased dorsolateral prefrontal cortical activity in response to emotional stimuli in euthymic bipolar disorder: no associations with psychotropic medication load.	Bipolar Disord. 2008 Dec;10(8):916-27. doi: 10.1111/j.1399-5618.2008.00641.x.	YMRS: 1.37(2.67); HAMD-25: 1.94(2.59)	19594507	Positive valence during facial affect processing	Positive Valence	Approach Motivation	Happy condition vs baseline (main effect of the group): Patients > Healthy controls
						Negative valence during facial affect processing	Negative Valence	Potential Threat ("Anxiety")	Fearful condition vs baseline (main effect of the group): Patients < Healthy controls
Hulvershorn	2012	Neural activation during facial emotion processing in unmedicated bipolar depression, euthymia, and mania.	Biol Psychiatry. 2012 Apr 1;71(7):603-10. doi: 10.1016/j.biopsych.2011.10.038.	YMRS: 1.37(2.67); HAMD: 1.94(2.59)	22206876	Negative valence during facial affect processing	Negative Valence	Potential Threat ("Anxiety")	Faces vs shapes: Manic BD patients > Healthy controls
Jogia	2008	Pilot investigation of the changes in cortical activation during facial affect recognition with lamotrigine monotherapy in bipolar disorder.	Br J Psychiatry. 2008 Mar;192(3):197-201. doi: 10.1192/bjp.bp.107.037960.	YMRS: 1(1.3); HAMD: 13.75(2.43)	18310580	Negative valence during facial affect processing	Negative Valence	Potential Threat ("Anxiety")	Pre-treatment, sad vs neutral facial affect: Patients > Healthy controls; Patients < Healthy controls
Keener	2012	Dissociable patterns of medial prefrontal and amygdala activity to face identity versus emotion in bipolar disorder.	Psychol Med. 2012 Sep;42(9):1913-24. doi: 10.1017/S0033291711002935	YMRS: 2.15(2.41); HAMD: 6.04(4.5)	22273442	Negative valence during facial affect processing	Negative Valence	Potential Threat ("Anxiety")	Fear faces vs shapes: BD patients >

© 2019 Janiri D et al. *JAMA Psychiatry.*

eTable 4: Bipolar Disorder: Studies included									
**First Author**	**Year**	**Title**	**Publication**	**Patients' Symptom Severity**	**PMID**	**Task**	**RDoC Domain**	**RDoC Construct**	**Contrasts Used**
									Healthy controls
						Positive valence during facial affect processing	Positive Valence	Approach Motivation	Happy faces vs shapes: BDpatients > Healthy controls
Killgore	2008	Abnormal corticostriatal activity during fear perception in bipolar disorder.	Neuroreport. 2008 Oct 8;19(15):1523-7. doi: 10.1097/WNR.0b013e328310af58	YMRS: 14.3(8.9); HAMD: 15.6(9.9)	18797310	Negative valence during facial affect processing	Negative Valence	Potential Threat ("Anxiety")	Fear faces vs fixation conditions, Block A and Block B: Patients > Healthy controls; Patients < Healthy controls
Kim	2009	Reduced activation in the mirror neuron system during a virtual social cognition task in euthymic bipolar disorder.	Prog Neuropsychopharmacol Biol Psychiatry. 2009 Nov 13;33(8):1409-16. doi: 10.1016/j.pnpbp.2009.07.019.	YMRS: 1.5(1.5); HAMD: 2.4(1.7)	19632283	Unspecified valence during facial affect processing	Social Process	Social Communication	Neutral vs control block: BD patients < Healthy controls
						Positive valence during facial affect processing	Positive Valence	Approach Motivation	Happy vs control block: BD patients > Healthy controls; BD patients < Healthy controls
						Negative valence during facial affect processing	Negative Valence	Potential Threat	Angry vs control block: BD

© 2019 Janiri D et al. *JAMA Psychiatry.*

eTable 4: Bipolar Disorder: Studies included

First Author	Year	Title	Publication	Patients' Symptom Severity	PMID	Task	RDoC Domain	RDoC Construct	Contrasts Used
								("Anxiety")	patients < Healthy controls
Mahli	2007_b	Is a lack of disgust something to fear? A functional magnetic resonance imaging facial emotionrecognition study in euthymic bipolar disorder patients.	Bipolar Disord. 2007 Jun;9(4):345-57. doi: 10.1111/j.1399-5618.2007.00485.x	YMRS: 0.9(0.5); HAMD: 4.4(1.1)	17547581	Negative valence during facial affect processing	Negative Valence	Potential Threat ("Anxiety")	Fear and disgust faces vs neutral faces:Patients > Healthy controls; Patients < Healthy controls
Mahli	2008	A functional MRI study of Theory of Mind in euthymic bipolar disorder patients.	Bipolar Disord. 2008 Dec;10(8):943-56. doi: 10.1111/j.1399-5618.2008.00643.x.	YMRS: 1.6(0.7); HAMD: 4.2(1.5)	19594509	Theory of Mind	Social Process	Perception and Understanding of Others (Interpersonal)	ToM animated sequences vs random-motion animated sequences: Euthymic bipolar patients < Healthy controls
Marchand	2011	Aberrant emotional processing in posterior cortical midline structures in bipolar II depression.	Prog Neuropsychopharmacol Biol Psychiatry. 2011 Aug 15;35(7):1729-37. doi: 10.1016/j.pnpbp.2011.05.017.	YMRS: 2.8(1.6); MADRS: 27.5(7.3)	21664220	Positive valence during facial affect processing	Positive Valence	Approach Motivation	Happy vs neutral: Patients < Healthy controls
Perlman	2012	Amygdala activity and prefrontal cortex-amygdala effective connectivity to emerging emotional faces distinguish remitted and depressed mood states in bipolar disorder.	Bipolar Disord. 2012 Mar;14(2):162-74. doi: 10.1111/j.1399-5618.2012.00999.x.	YMRS: 2.39(2.5); HAMD: 7.29(5.53)	22420592	Unspecified valence during facial affect processing	Social Process	Social Communication	Response to all faces vs shapes: Remitted patients > Healthy controls
Sepede	2015	Neural correlates of negative emotion processing in bipolar disorder.	Prog Neuropsychopharmacol Biol Psychiatry. 2015 Jul 3;60:1-10. doi: 10.1016/j.pnpbp.2015.01.016.	YMRS: 0.6(0.8); HAMD: 2.4(2.6)	2566	Negative valence during passive scene viewing	Negative Valence	Potential Threat	Negative vs neutral:

© 2019 Janiri D et al. *JAMA Psychiatry.*

eTable 4: Bipolar Disorder: Studies included									
**First Author**	**Year**	**Title**	**Publication**	**Patients' Symptom Severity**	**PMID**	**Task**	**RDoC Domain**	**RDoC Construct**	**Contrasts Used**
					1850			("Anxiety")	Patients > Healthy controls; Patients < Healthy controls
Surguladze	2010	Exaggerated neural response to emotional faces in patients with bipolar disorder and their first-degree relatives	Neuroimage. 2010 Oct 15;53(1):58-64. doi: 10.1016/j.neuroimage.2010.05.069.	Altman Self rating scale mania: 3.39(2.45); BDI: 7.26(6.08)	20595014	Unspecified valence during facial affect processing	Social Process	Social Communication	Group×emotion×intensity interaction: Patients > Healthy control
Tseng	2016	Functional connectivity during masked and unmasked face emotion processing in bipolardisorder.	Psychiatry Res Neuroimaging. 2016 Dec 30;258:1-9. doi: 10.1016/j.pscychresns.2016.10.006.	YMRS: 4(3.19); HAMD: 16.08(9.37)	27814457	Unspecified valence during facial affect processing	Social Process	Social Communication	Emotions vs no face: BD patients > Healthy control
Whalley	2009	Functional imaging of emotional memory in bipolar disorder and schizophrenia.	Bipolar Disord. 2009 Dec;11(8):840-56. doi: 10.1111/j.1399-5618.2009.00768.x.	YMRS: 3.64(4.27); HAMD: 6.57(7.85)	19922553	Unspecified valence during passive scene viewing	Unclassified	Unclassified	Emotional scenes vs neutral scenes: Patients > Healthy controls
Vizueta	2012	Regional fMRI hypoactivation and altered functional connectivity during emotion processing in nonmedicated depressed patients with bipolar II disorder.	Am J Psychiatry. 2012 Aug;169(8):831-40. doi: 10.1176/appi.ajp.2012.11030349.	YMRS: 2.9(2.2); HAMD: 19.9(3.8)	22773540	Unspecified valence during facial affect processing	Social Process	Social Communication	Match emotions vs match forms: Depressed bipolar II patients < Healthy controls
**Studies using experiments mapping the RDoC cognitive systems domain**									

© 2019 Janiri D et al. *JAMA Psychiatry.*

eTable 4: Bipolar Disorder: Studies included									
**First Author**	**Year**	**Title**	**Publication**	**Patients' Symptom Severity**	**PMID**	**Task**	**RDoC Domain**	**RDoC Construct**	**Contrasts Used**
Allin	2010	A functional MRI study of verbal fluency in adults with bipolar disorder and their unaffected relatives.	Psychol Med. 2010 Dec;40(12):2025-35. doi: 10.1017/S0033291710000127.	ASRM: 3.5(2.4); BDI: 7.2(5.9)	20146832	Verbal fluency task	Cognitive System	Language	Hard condition vs control condition: Patients > Healthy controls
Alonso-Lana	2016_a	Brain functional changes in first-degree relatives of patients with bipolar disorder: evidence for default mode network dysfunction.	Psychol Med. 2016 Sep;46(12):2513-21. doi: 10.1017/S0033291716001148.	YMRS: 0.79(1.32); HAMD: 2.53(2.32)	27334766	N-back task	Cognitive System	Working memory	2-back vs baseline: Patients > Healthy controls
Alonso-Lana	2016_b	Structural and Functional Brain Correlates of Cognitive Impairment in Euthymic Patients with Bipolar Disorder.	PLoS One. 2016 Jul 22;11(7):e0158867. doi: 10.1371/journal.pone.0158867.	YMRS: 1.18(1.81); HAMD: 2.55(2.02)	27448153	N-back task	Cognitive System	Working memory	2-back vs 1-back: Cognitively preserved patients > Healthy controls
Altshuler	2005	Blunted activation in orbitofrontal cortex during mania: a functional magnetic resonance imagingstudy.	Biol Psychiatry. 2005 Nov 15;58(10):763-9. DOI: 10.1016/j.biopsych.2005.09.012	YMRS: 16.9(3.9); HAMD: 5.36(4.41)	16310510	Go/no-go task	Cognitive System	Cognitive control	NoGo vs Go Tasks: Manic patients < Healthy controls
Brooks	2015	Prefrontal hypoactivation during working memory in bipolar II depression.	Psychol Med. 2015 Jun;45(8):1731-40. doi: 10.1017/S0033291714002852.	YMRS: 2.7(1.9); HAMD: 18.6(3.3)	25752642	N-back task	Cognitive System	Working memory	1-2-back vs 0-back: Bipolar II depressed patients < Healthy controls
Costafreda	2011	Pattern of neural responses to verbal fluency shows diagnostic specificity for schizophrenia and bipolar disorder.	BMC Psychiatry. 2011 Jan 28;11:18. doi: 10.1186/1471-244X-11-18.	YMRS: 2(3.71); HAMD: 5.44(8.61)	21276242	Verbal fluency task	Cognitive System	Language	Word production vs baseline: Bipolar patients > Healthy controls
Curtis	2007	The nature of abnormal language processing in euthymic bipolar I disorder: evidence for a relationship between task demand and prefrontal function.	Bipolar Disord. 2007 Jun;9(4):358-69. doi: 10.1111/j.1399-5618.2007.00422.x	YMRS: 1.1(1.2); HAMD: 2.3(2.9)	1754758	Semantic judgment task	Cognitive System	Language	Overall task (phonetic and semantic) vs combined baseline conditions (word repetition and word form analysis): Patients > Healthy controls; Patients <

© 2019 Janiri D et al. *JAMA Psychiatry.*

eTable 4: Bipolar Disorder: Studies included

First Author	Year	Title	Publication	Patients' Symptom Severity	PMID	Task	RDoC Domain	RDoC Construct	Contrasts Used
									Healthy controls
Fernandez-Corcuera	2013	Bipolar depressed patients show both failure to activate and failure to de-activate during performance of a working memory task.	J Affect Disord. 2013 Jun;148(2-3):170-8. doi: 10.1016/j.jad.2012.04.009.	HAMD: 22.55(4.4)	22854099	N-back task	Cognitive System	Working memory	2-back vs baseline: Patients > Healthy controls; Patients < Healthy controls
Fleck	2011	Preliminary evidence for increased frontosubcortical activation on a motor impulsivity task in mixed episode bipolar disorder.	J Affect Disord. 2011 Sep;133(1-2):333-9. doi: 10.1016/j.jad.2011.03.053.	YMRS: 24(2); MADRS: 22(5)	21546091	Go/no-go task	Cognitive System	Cognitive control	Correct rejection, omission and commission error trials vs go: Bipolar patients in Mixed episode> Healthy controls
Frangou	2012	Brain structural and functional correlates of resilience to Bipolar Disorder.	Front Hum Neurosci. 2012 Jan 27;5:184. doi: 10.3389/fnhum.2011.00184.	YMRS: 1.3(2.9); HAMD: 3(3.9)	22363273	Stroop task	Cognitive System	Cognitive control	Incongruent vs neutral condition: Bipolar Patients < Healthy Controls
Glahn	2010	Fronto-temporal dysregulation in asymptomatic bipolar I patients: a paired associate functionalMRI study.	Hum Brain Mapp. 2010 Jul;31(7):1041-51. doi: 10.1002/hbm.20918.	YMRS: 1(0.85); HAMD: 2.67(1.67)	20063304	Paired associates learning task	Cognitive System	Declarative Memory	Encoding and Recognition vs distractor task: Patients > Healthy controls; Patients < Healthy controls
Gruber	2010	Pathological amygdala activation during working memory performance: Evidence for a pathophysiological trait marker in bipolar affective disorder.	Hum Brain Mapp. 2010 Jan;31(1):115-25. doi: 10.1002/hbm.20849.	YMRS: 2.3(0.9); HAMD: 0.3(0.2)	19603410	Delayed match-to-sample task	Cognitive System	Working memory	Articulatory rehearsal vs control condition: Bipolar patients > healthy controls
Hamilton	2009	Alterations in functional activation in euthymic bipolar disorder and schizophrenia during a working memory task.	Hum Brain Mapp. 2009 Dec;30(12):3958-69. doi: 10.1002/hbm.20820.	YMRS: 2(2.4); HAMD: 4.6(2.3)	19449330	WM Task	Cognitive System	Working memory	WM vs rest: BD Patients < Healthy controls

© 2019 Janiri D et al. *JAMA Psychiatry.*

eTable 4: Bipolar Disorder: Studies included									
**First Author**	**Year**	**Title**	**Publication**	**Patients' Symptom Severity**	**PMID**	**Task**	**RDoC Domain**	**RDoC Construct**	**Contrasts Used**
Jogia	2012	Frontopolar cortical inefficiency may underpin reward and working memory dysfunction in bipolar disorder.	World J Biol Psychiatry. 2012 Dec;13(8):605-15. doi: 10.3109/15622975.2011.585662.	YMRS: 1.7(2.7); HAMD: 1.8(5.1)	21812622	N-back task	Cognitive System	Working memory	3-Back vs 0-back: BD patients > Healthy controls; BD patients < Healthy controls
Joshi	2016	elationships Between Altered Functional Magnetic Resonance Imaging Activation and Cortical Thickness in Patients With Euthymic Bipolar I Disorder.	Biol Psychiatry Cogn Neurosci Neuroimaging. 2016 Nov;1(6):507-517. doi: 10.1016/j.bpsc.2016.06.006	YMRS: 1.7(2); HAMD: 1.7(2)	27990494	Go/no-go task	Cognitive System	Cognitive control	NoGo vs Go: Patients < Healthy controls
Kaladjian	2009_a	Reduced brain activation in euthymic bipolar patients during response inhibition: an event-related fMRI study.	Psychiatry Res. 2009 Jul 15;173(1):45-51. doi: 10.1016/j.pscychresns.2008.08.003.	YMRS: 2.2(2); HAMD: 2.2(1.6)	19442494	Go/no-go task	Cognitive System	Cognitive control	Correct NoGo vs correct Go (main effect of the group): Euthymic bipolar patients < Healthy controls
Kaladjian	2009_b	Remission from mania is associated with a decrease in amygdala activation during motor response inhibition.	Bipolar Disord. 2009 Aug;11(5):530-8. doi: 10.1111/j.1399-5618.2009.00722.x.	YMRS: 24.6(4.3); HAMD: 4.3(2.5)	19624392	Go/no-go task	Cognitive System	Cognitive control	Correct NoGo vs correct Go:T1,Patients < Healthy controls
Kronhaus	2006	Stroop performance in bipolar disorder: further evidence for abnormalities in the ventral prefrontal cortex.	Bipolar Disord. 2006 Feb;8(1):28-39. doi: 10.1111/j.1399-5618.2006.00282.x	YMRS range: [2-7]; BDI: 14(5.9)	16411978	Stroop task	Cognitive System	Cognitive control	Stroop vs control condition: Patients < Healthy controls
Lagopoulos	2007	An event-related functional MRI study of working memory in euthymic bipolar disorder.	J Psychiatry Neurosci. 2007 May;32(3):174-84.	YMRS: 0.9(0.8); HAMD: 4.2(1)	17476364	Sternberg task	Cognitive System	Working memory	Encode, delay and response conditions vs baseline: BD patients > Healthy controls; BD patients < Healthy controls
Mazzola-Pomietto	2009	Bilateral decrease in ventrolateral prefrontal cortex activation during motor response inhibition in mania.	J Psychiatr Res. 2009 Jan;43(4):432-41. doi: 10.1016/j.jpsychires.2008.05.004.	YMRS: 22.4(5.4); HAMD: 4.5(2.6)	18586275	Go/no-go task	Cognitive System	Cognitive control	Correct NoGo trials vs correct Go trials: Patients < Healthy controls

© 2019 Janiri D et al. *JAMA Psychiatry.*

eTable 4: Bipolar Disorder: Studies included

First Author	Year	Title	Publication	Patients' Symptom Severity	PMID	Task	RDoC Domain	RDoC Construct	Contrasts Used
McIntosh	2008	Prefrontal function and activation in bipolar disorder and schizophrenia.	Am J Psychiatry. 2008 Mar;165(3):378-84. doi: 10.1176/appi.ajp.2007.07020365.	YMRS: 0.6(1.2); HAMD: 2.3(5.9)	18198268	Sentence Completion Task	Cognitive System	Language	Sentence completion vs rest condition: Patients < Healthy controls
Mckenna	2014	Abnormalities of brain response during encoding into verbal working memory among euthymic patients with bipolar disorder.	Bipolar Disord. 2014 May;16(3):289-99. doi: 10.1111/bdi.12126.	YMRS: 1.22(1.4); HAMD: 3.22(2.28)	24119150	Delayed match-to-sample task	Cognitive System	Working memory	Encode and maintenance intervals vs baseline: Bipolar patients > Healthy controls; Bipolar Patients < Healthy controls
Oertel-Knochel	2013	Verbal episodic memory deficits in remitted bipolar patients: a combined behavioural and fMRIstudy.	J Affect Disord. 2013 Sep 5;150(2):430-40. doi: 10.1016/j.jad.2013.04.036.	Bech–Rafaelsen Mania Scale: 4.46(4.16); BDI II: 13.42(10.9)	23764381	Episodic memory encoding/retrieval task	Cognitive System	Declarative Memory	Encoding and retrieval vs baseline: Patients < Healthy controls
Oertel-Knochel	2014	Episodic memory impairments in bipolar dis order are associated with functional and str ucturalbrain changes.	Bipolar Disord. 2014 Dec;16(8):830-45. doi: 10.1111/bdi.12241.	BRMAS : 0.38(0.59); BDI II: 9.85(8.97)	25164120	Episodic memory encoding/retrieval task	Cognitive System	Declarative Memory	Non verbal encoding and Non-verbal retrieval vs baseline: Patients > Healthy controls; Patients < Healthy controls
Pomarol-Clotet	2012	Failure of de-activation in the medial frontal cortex in mania: evidence for default mode networkdysfunction in the disorder.	World J Biol Psychiatry. 2012 Dec;13(8):616-26. doi: 10.3109/15622975.2011.573808	YMRS: 21.76(3.29)	21604958	N-back task	Cognitive System	Working memory	2-back vs baseline: Patients > Healthy controls; Patients < Healthy controls
Pomarol-Clotet	2015	Brain functional changes across the differen t phases of bipolar disorder.	Br J Psychiatry. 2015 Feb;206(2):136-44. doi: 10.1192/bjp.bp.114.152033.	Manic Patients - YMRS: 21.84(3.67); HAMD: 4.14(3.65) Depressed Patients - YMRS: 1.29(2.24); HAMD: 22.13(4.03)	25497296	N-back task	Cognitive System	Working memory	2-back vs baseline: Manic patients > Healthy controls; Manic patients <

© 2019 Janiri D et al. *JAMA Psychiatry.*

eTable 4: Bipolar Disorder: Studies included

First Author	Year	Title	Publication	Patients' Symptom Severity	PMID	Task	RDoC Domain	RDoC Construct	Contrasts Used
				Euthymic Patients - YMRS: 1.41(1.88); HAMD: 2.73(2.18)					Healthy controls
									2-back vs baseline: Depressed patients > Healthy controls; Depressed patients < Healthy controls
									2-back vs baseline: Euthymic patients > Healthy controls
Penfold	2015	Frontal lobe hypoactivation in medication-free adults with bipolar II depression during response inhibition.	Psychiatry Res. 2015 Mar 30;231(3):202-9. doi: 10.1016/j.pscychresns.2014.11.005	YMRS: 2.6(1.9); HAMD: 18.5(3.4)	25555505	Go/no-go task	Cognitive System	Cognitive control	NoGo vs Go: BD depressed patients < Healthy controls
Pompei	2011	Familial and disease specific abnormalities in the neural correlates of the Stroop Task in Bipolar Disorder.	Neuroimage. 2011 Jun 1;56(3):1677-84. doi: 10.1016/j.neuroimage.2011.02.052.	YMRS: 1.46(3.02); HAMD: 4.92(5.4)	21352930	Stroop task	Cognitive System	Cognitive control	Incongruent vs congruent condition: Patients < Healthy controls
Robinson	2009	Fronto-temporal dysregulation in remitted bipolar patients: an fMRI delayed-non-match-to-sample (DNMS) study.	Bipolar Disord. 2009 Jun;11(4):351-60. doi: 10.1111/j.1399-5618.2009.00703.x.	YMRS: 1.6(1.99); HAMD: 3.93(3.04)	19500088	Delayed-non-match-to-sample task	Cognitive System	Working memory	Response to familiarity and novelty conditions vs baseline: Patients > Healthy controls; Patients < Healthy controls
Roth	2006	Functional magnetic resonance imaging of executive control in bipolar disorder.	Neuroreport. 2006 Jul 31;17(11):1085-9. doi: 10.1097/01.wnr.0000227979.06013.57	YMRS: 8.6(9.1); HAMD: 4.6(6)	16837832	Stroop task	Cognitive System	Cognitive control	Incongruent vs congruent condition: Patients < Healthy controls
Sapede	2012	Impaired sustained attention in euthymic bipolar disorder patients and non-affected relatives: an fMRI study.	Bipolar Disord. 2012 Nov;14(7):764-79. doi: 10.1111/bdi.12007.	YMRS: 0.6(0.8); HAMD: 2.5(2.6)	23036083	Continuos performance task	Cognitive System	Attention	Correct target condition and incorrect target condition vs

© 2019 Janiri D et al. *JAMA Psychiatry.*

eTable 4: Bipolar Disorder: Studies included									
**First Author**	**Year**	**Title**	**Publication**	**Patients' Symptom Severity**	**PMID**	**Task**	**RDoC Domain**	**RDoC Construct**	**Contrasts Used**
									baseline: BD patients > Healthy controls; BD patients < Healthy controls
Strakowski	2005	Abnormal FMRI brain activation in euthymic bipolar disorder patients during a counting Stroop interference task.	Am J Psychiatry. 2005 Sep;162(9):1697-705. doi: 10.1176/appi.ajp.162.9.1697	YMRS: 1.6(1.8); HAMD: 3(2.3)	16135630	Stroop task	Cognitive System	Cognitive control	Incongruent vs congruent condition: Bipolar patients > Healthy controls; Bipolar patients < Healthy controls
Townsend	2012	Deficits in inferior frontal cortex activation in euthymic bipolar disorder patients during a response inhibition task.	Bipolar Disord. 2012 Jun;14(4):442-50. doi: 10.1111/j.1399-5618.2012.01020.x.	YMRS: 1.4(2); HAMD: 3.8(2)	22631623	Go/no-go task	Cognitive System	Cognitive control	NoGo vs Go: Euthymic bipolar Patients < Healthy controls
Weathers	2013	A developmental study on the neural circuitry mediating response flexibility in bipolar disorder.	Psychiatry Res. 2013 Oct 30;214(1):56-65. doi: 10.1016/j.pscychresns.2013.05.002	YMRS: 3.9(4.9); SIGH-SAD : 16.62(11.4)	23958598	Stop task	Cognitive System	Cognitive control	Successful Change vs Go trials: Adult BD < Adult healthy Subject
**Studies including cross-domain experiments**									
Cerullo	2014	Bipolar I disorder and major depressive disorder show similar brain activation during depression.	Bipolar Disord. 2014 Nov;16(7):703-12. doi: 10.1111/bdi.12225.	YMRS: 8(6); HAMD: 32(7)	24990479	Emotional visual oddball task	Cognitive-affective interaction	Cognitive-affective interaction	Emotional images vs neutral; circles vs square trials: Patients > Healthy controls; Patients < Healthy controls
Favre	2013	Modulation of fronto-limbic activity by the psychoeducation in euthymic bipolar patients. A functional MRI study.	Psychiatry Res. 2013 Dec 30;214(3):285-95. doi: 10.1016/j.pscychresns.2013.07.007.	YMRS: 2.23(2.24); MADRS: 10.31(9.05)	24156926	Emotional Stroop task	Cognitive-affective interaction	Cognitive-affective interaction	Incongruent vs congruent: Patients < Healthy controls

© 2019 Janiri D et al. *JAMA Psychiatry.*

eTable 4: Bipolar Disorder: Studies included

First Author	Year	Title	Publication	Patients' Symptom Severity	PMID	Task	RDoC Domain	RDoC Construct	Contrasts Used
Favre	2015	Cerebral Correlates of Abnormal Emotion Conflict Processing in Euthymic Bipolar Patients: A Functional MRI Study.	PLoS One. 2015 Aug 5;10(8):e0134961. doi: 10.1371/journal.pone.0134961.	YMRS: 2.92(3.17); MADRS: 7.61(5.01)	26244883	Emotional Stroop task	Cognitive-affective interaction	Cognitive-affective interaction	Low conflict vs high conflict: Patients < Healthy controls
Malhi	2005	An emotional Stroop functional MRI study of euthymic bipolar disorder.	Bipolar Disord. 2005;7 Suppl 5:58-69. doi: 10.1111/j.1399-5618.2005.00255.x	YMRS: 0.9(0.5); HAMD: 4.3(1.1)	16225562	Emotional Stroop task	Cognitive-affective interaction	Cognitive-affective interaction	Affective words vs neutral words:Patients < Healthy controls
Mahli	2007_a	Reduced activation to implicit affect induction in euthymic bipolar patients: an fMRI study.	J Affect Disord. 2007 Jan;97(1-3):109-22 doi: 10.1016/j.jad.2006.06.005	YMRS: 0.9(0.8); HAMD: 3.1(1)	16837058	Emotional Sternberg	Cognitive-affective interaction	Cognitive-affective interaction	Negative and positive affect vs neutral: Patients < Healthy controls
Townsend	2013	Frontal-amygdala connectivity alterations during emotion downregulation in bipolar I disorder.	Biol Psychiatry. 2013 Jan 15;73(2):127-35. doi: 10.1016/j.biopsych.2012.06.030.	YMRS: 1.7(2.2); HAMD: 3.8(1.9)	22858151	Emotion regulation task	Cognitive-affective interaction	Cognitive-affective interaction	Decrease negative vs observe negative: Bipolar atients < Healthy controls
Wessa	2007	Fronto-striatal overactivation in euthymic bipolar patients during an emotional go/nogo task.	Am J Psychiatry. 2007 Apr;164(4):638-46. DOI: 10.1176/ajp.2007.164.4.638	YMRS: 0.65(1.97); HAMD: 1.35(1.41)	17403978	Emotional Go-No	Cognitive-affective interaction	Cognitive-affective interaction	Emotional distractors vs neutral distractors (nogo): Bipolar patients > Healthy controls
Rey	2014	Modulation of brain response to emotional conflict as a function of current mood in bipolar disorder: preliminary findings from a follow-up state-based fMRI study.	Psychiatry Res. 2014 Aug 30;223(2):84-93. doi: 10.1016/j.pscychresns.2014.04.016.	YMRS: 0.5(0.6); MADRS: 15.3(3.4)	24862389	Emotional Stroop task	Cognitive-affective interaction	Cognitive-affective interaction	Incongruent vs congruent: Depressed BD Patients < Healthy controls

Patients' Symptom severity is provided as mean (standard deviation) when available.
Abbreviations: ASRM: Altman Self rating scale mania; YMRS: Young- Mania Rating Scale, HAMD: Hamilton Depression Rating Sale; MADRS: Montgomery-Åsberg Depression Rating Scale; BDI: Beck Depression Inventory.

© 2019 Janiri D et al. *JAMA Psychiatry.*

eTable 5: Post-Traumatic Stress Disorder and Anxiety Disorders: Studies Included

First Author	Year	Title	Publication	Patients' Symptom Severity	PMID	Task	RDoC Domain	RDoC Construct	Contrasts Used
**Studies using experiments mapping the RDoC domains of negative valence systems, positive valence systems and social processes**									
Amir	2005	Increased activation of the anterior cingulate cortex during processing of disgust faces in individuals with social phobia.	Biol Psychiatry. 2005 May 1;57(9):975-81. doi: 10.1016/j.biopsych.2005.01.044	N.A.	15860337	Negative valence during facial affect processing	Negative Valence	Potential Threat ("Anxiety")	Disgust vs neutral Faces: Patients with Social Phobia > Healthy controls
Aue	2015	Brain systems underlying encounter expectancy bias in spider phobia.	Cogn Affect Behav Neurosci. 2015 Jun;15(2):335-48. doi: 10.3758/s13415-015-0339-6	N.A.	25694215	Symptom Provocation Paradigm	Negative Valence	Acute Threat ("Fear")	Expectancy spider vs expectancy snake: Patients with specific phobia > Healthy controls
Aupperle	2012	Dorsolateral prefrontal cortex activation during emotional anticipation and neuropsychological performance in posttraumatic stress disorder.	Arch Gen Psychiatry. 2012 Apr;69(4):360-71. doi: 10.1001/archgenpsychiatry.2011.1539	CAPS: 70.57(17.58)	22474105	Unspecified valence during passive scene viewing	Unclassified	Unclussified	Anticipation of negative images vs anticipation of positive images: Patients with PTSD > Healthy controls; Patients < Healthy controls
Blair	2008	Response to emotional expressions in generalized social phobia and generalized anxietydisorder: evidence for separate disorders.	Am J Psychiatry. 2008 Sep;165(9):1193-202. doi: 10.1176/appi.ajp.2008.07071060	BAI: 9.4(7.1)	18483136	Negative valence during facial affect processing	Negative Valence	Potential Threat ("Anxiety")	Fearful vs neutral expressions: Patients with generalized social phobia without GAD > Healthy controls
Bruhl	2011	Neural correlates of altered general emotion processing in social anxiety disorder.	Brain Res. 2011 Mar 10;1378:72-83. doi: 10.1016/j.brainres.2010.12.084.	Social Phobia Scale: 28(12.02)	21215728	Negative valence during passive scene viewing	Negative Valence	Potential Threat ("Anxiety")	Expectation negative vs expectation neutral: Patients with social anxiety disorder > Healthy controls; Patients < Healthy controls
Brunetti	2010	Elevated response of human amygdala to neutral stimuli in mild post traumatic stress disorder: neural correlates of generalized emotional response.	Neuroscience. 2010 Jul 14;168(3):670-9. doi: 10.1016/j.neuroscience.2010.04.024	CAPS: 30.8(NA)	20416363	Unspecified valence during passive scene viewing	Unclassified	Unclassified	Group and valence interaction: PTSD Patients > Healthy controls

© 2019 Janiri D et al. *JAMA Psychiatry.*

eTable 5: Post-Traumatic Stress Disorder and Anxiety Disorders: Studies Included

First Author	Year	Title	Publication	Patients' Symptom Severity	PMID	Task	RDoC Domain	RDoC Construct	Contrasts Used
Demenescu	2011	Neural correlates of perception of emotional facial expressions in out-patients with mild-to-moderate depression and anxiety. A multicenter fMRI study.	Psychol Med. 2011 Nov;41(11):2253-64. doi: 10.1017/S0033291711000596	BAI: 13.86(9.89)	21557888	Positive valence during facial affect processing	Positive Valence	Approach Motivation	Happy vs scrambled: Patients with anxiety disorders < Healthy controls
Evans	2008	A functional MRI study of amygdala responses to angry schematic faces in social anxietydisorder.	Depress Anxiety. 2008;25(6): 496-505.DOI: 10.1002/da.20347	BAI: 11.1(8.5)	17595018	Negative valence during facial affect processing	Negative Valence	Potential Threat ("Anxiety")	Anger vs neutral: SAD patients > Healthy controls; SAD patients < Healthy controls
Fonzo	2010	Exaggerated and disconnected insular-amygdalar blood oxygenation level-dependent responseto threat-related emotional faces in women with intimate-partner violence posttraumatic stress disorder.	Biol Psychiatry. 2010 Sep 1;68(5):433-41. doi: 10.1016/j.biopsych.2010.04.028	Overall Anxiety Severity and Impairment Scale: 10.38(3.68)	20573339	Unspecified valence during facial affect processing	Social Process	Social Communication (Mixed)	Matching to a Fearful vs Happy Target Face and Angry vs Happy Target Face: PTSD Patients > Healthy controls; PTSD Patients < Healthy controls
Fonzo	2014	Cognitive-behavioral therapy for generalized anxiety disorder is associated with attenuation of limbic activation to threat-related facial emotions.	J Affect Disord. 2014 Dec;169:76-85. doi: 10.1016/j.jad.2014.07.031	N.A.	25171782	Unspecified valence during facial affect processing	Social Process	Social Communication (Mixed)	Targeted processing of fear and angry vs happy: GAD Patients > Healthy controls
Fonzo	2015	Common and disorder-specific neural responses to emotional faces in generalised anxiety, social anxiety and panic disorders.	Br J Psychiatry. 2015 Mar;206(3):206-15. doi: 10.1192/bjp.bp.114.149880	GAD Group: STAI-State: 53.2(8.47)	25573399	Unspecified valence during facial affect processing	Social Process	Social Communication	Processing all faces vs shapes: GAD Patients> Healthy controls; Patients with panic disorder < Healthy controls; Patients with panic disorder > controls
Frick	2013	Altered fusiform connectivity during processing of fearful faces in social anxiety disorder.	Transl Psychiatry. 2013 Oct 8;3:e312. doi: 10.1038/tp.2013.85	LSAS-SR: 72.1(25.7)	25573399	Negative valence during facial affect processing	Negative Valence	Potential Threat ("Anxiety")	Fearful vs neutral faces: SAD Patients > Healthy controls
Gentili	2008	Differential modulation of neural activity throughout the distributed neural system for faceperception in patients with Social Phobia and healthy subjects.	Brain Res Bull. 2008 Nov 25;77(5):286-92. doi: 10.1016/j.brainresbull.2008.08.003	LSSP: 69.6(1.01)	18771714	Unspecified valence during facial affect processing	Social Process	Social Communication (Mixed)	Response to faces vs scrambled pictures: Patients with social phobia > Healthy controls; Patients with social phobia < Healthy controls
Goossens	2007	Visual presentation of phobic stimuli: amygdala activation via an extrageniculostriate pathway?	Psychiatry Res. 2007 Jul 15;155(2):113-20. DOI: 10.1016/j.pscychresns.2006.12.005	SPQ: 21(2)	17499485	Symptom Provocation Paradigm	Negative Valence	Acute Threat ("Fear")	Spider vs neutral pictures: Phobic Patients > Healthy controls
Hou	2007	Brain responses to symptom provocation and trauma-related short-term memory recall in coalmining accident survivors with acute severe PTSD.	Brain Res. 2007 May 4;1144:165-74. doi: 10.1016/j.brainres.2007.01.089	CAPS: 83.6(8.28)	17331476	Symptom Provocation Paradigm	Negative Valence	Acute Threat ("Fear")	Mining accident related pictures vs versus neutral pictures: PTSD Patients > Healthy controls; PTSD Patients < Healthy controls
Kim	2008	Diminished rostral anterior cingulate activity in response to threat-related events in posttraumatic stress disorder	J Psychiatr Res. 2008 Mar;42(4):268-77. doi: 10.1016/j.jpsychires.2007.02.003	HAMA: 7.5(3.4)	17400251	Negative valence during facial affect processing	Negative Valence	Potential Threat ("Anxiety")	Fearful vs neutral faces: PTSD Patients > Healthy controls; PTSD Patients < Healthy controls

© 2019 Janiri D et al. *JAMA Psychiatry.*

eTable 5: Post-Traumatic Stress Disorder and Anxiety Disorders: Studies Included									
**First Author**	**Year**	**Title**	**Publication**	**Patients' Symptom Severity**	**PMID**	**Task**	**RDoC Domain**	**RDoC Construct**	**Contrasts Used**
Kim	2016	Neural basis of distorted self-face recognition in social anxiety disorder.	Neuroimage Clin. 2016 Apr 28;12:956-964. DOI: 10.1016/j.nicl.2016.04.010	LSAS-SR: 84.8(17.1)	27995061	Self-referential processing task	Social Process	Perception and Understanding of Self (Self)	90% SELF condition vs emo: SAD Patients < Healthy controls
Klump	2010	Amygdala reactivity to faces at varying intensities of threat in generalized social phobia: an event-related functional MRI study.	Psychiatry Res. 2010 Aug 30;183(2):167-9. doi: 10.1016/j.pscychresns.2010.05.001	NA	20609570	Negative valence during facial affect processing	Negative Valence	Potential Threat ("Anxiety")	High threat intensity vs neutral: Patients with social phobia > Healthy controls
Klump	2012	Insula reactivity and connectivity to anterior cingulate cortex when processing threat in generalized social anxiety disorder.	Biol Psychol. 2012 Jan;89(1):273-6. doi: 10.1016/j.biopsycho.2011.10.010	State Anxiety Level, Spielberger State-Trait Inventory: 39.6(8.8)	22027088	Unspecified valence during facial affect processing	Social Process	Social Communication	Fearful vs happy faces: SAD Patients > Healthy controls
Labuschagne	2012	Medial frontal hyperactivity to sad faces in generalized social anxiety disorder and modulation by oxytocin.	Int J Neuropsychopharmacol. 2012 Aug 1;15(7):883-896. doi: 10.1017/S1461145711001489	BAI: 16.9(8.2)	21996304	Negative valence during facial affect processing	Negative Valence	Potential Threat ("Anxiety")	Baseline, sad vs neutral faces: GSAD Patients > Healthy controls
Lanius	2005	Functional connectivity of dissociative responses in posttraumatic stress disorder: a functionalmagnetic resonance imaging investigation.	Biol Psychiatry. 2005 Apr 15;57(8):873-84. DOI: 10.1016/j.biopsych.2005.01.011	CAPS: 90(7)	15820708	Self-referential memory paradigm	Social Process	Perception and Understanding of Self (Self)	Neutral memory Recall vs baseline: Dissociative PTSD > Healthy controls; Dissociative PTSD < Healthy controls
Linnman	2011	An fMRI study of unconditioned responses in post-traumatic stress disorder.	Biol Mood Anxiety Disord. 2011 Nov 1;1(1):8. doi: 10.1186/2045-5380-1-8	BAI: 19(13)	22738227	Fear conditioning	Negative Valence	Acute Threat ("Fear")	Unconditioned stimuli vs CS-: PTSD Patients > Healthy controls
Lueken	2011	How specific is specific phobia? Different neural response patterns in two subtypes of specificphobia.	Neuroimage. 2011 May 1;56(1):363-72. doi: 10.1016/j.neuroimage.2011.02.015	ASI total: 22(7.36)	21316468	Symptom Provocation Paradigm	Negative Valence	Acute Threat ("Fear")	Snake anxiety vs snake neutral: Snake phobics vs > Healthy controls
Lueken	2014	Altered top-down and bottom-up processing of fear conditioning in panic disorder with agoraphobia.	Psychol Med. 2014 Jan;44(2):381-94. doi: 10.1017/S0033291713000792	ASI total: 31.41(9.88)	23611156	Fear conditioning	Negative Valence	Acute Threat ("Fear")	Differential acquisition phase (CS+ unpaired vs CS-): Patients with agoraphobia > Healthy controls
Lueken	201	Neural substrates of defensive reactivity in two subtypes of specific phobia.	Soc Cogn Affect Neurosci. 2014 Nov;9(11):1668-75. doi: 10.1093/scan/nst159	ASI total: 20.62(9.71)	24174207	Symptom Provocation Paradigm	Negative Valence	Acute Threat ("Fear")	Snake anxiety vs snake neutral: Snake phobics vs > Healthy controls

© 2019 Janiri D et al. *JAMA Psychiatry.*

eTable 5: Post-Traumatic Stress Disorder and Anxiety Disorders: Studies Included

First Author	Year	Title	Publication	Patients' Symptom Severity	PMID	Task	RDoC Domain	RDoC Construct	Contrasts Used
	4b								
Mazza	2012	Neural correlates of automatic perceptual sensitivity to facial affect in posttraumatic stressdisorder subjects who survived L'Aquila eartquake of April 6, 2009.	Brain Imaging Behav. 2012 Sep;6(3):374-86. doi: 10.1007/s11682-012-9151-x	CAPS Total: 87.9(17.45)	22367971	Unspecified valence during facial affect processing	Social Process	Social Communication (Mixed)	Imagine block vs baseline: PTSD Patients > Healthy controls; PTSD Patients < Healthy controls
Mazza	2013	Regional cerebral changes and functional connectivity during the observation of negativeemotional stimuli in subjects with post-traumatic stress disorder.	Eur Arch Psychiatry Clin Neurosci. 2013 Oct;263(7):575-83. doi: 10.1007/s00406-013-0394-3	CAPS Total: 78.2(21.18)	23385487	Negative valence during passive scene viewing	Negative Valence	Potential Threat ("Anxiety")	Emotional Pictures vs neutral stimuli: PTSD Patients > Healthy controls; PTSD Patients < Healthy controls
Morey	2015	Fear learning circuitry is biased toward generalization of fear associations in posttraumatic stress disorder.	Transl Psychiatry. 2015 Dec 15;5:e700. doi: 10.1038/tp.2015.196	CAPS: 86.25(18.7)	26670285	Fear conditioning	Negative Valence	Acute Threat ("Fear")	Cs+ vs CS-: PTSD Patients > Healthy controls
Moser	2014	Violence-related PTSD and neural activation when seeing emotionally charged male-femaleinteractions.	Soc Cogn Affect Neurosci. 2015 May;10(5):645-53. doi: 10.1093/scan/nsu099	CAPS: >55	25062841	Unspecified valence during facial affect processing	Social Process	Social Communication	Emotional vs neutral: IPV-PTSD Patients > Healthy controls; IPV-PTSD Patients < Healthy controls
Mueller-Pfeiffer	2013	Atypical visual processing in posttraumatic stress disorder.	Neuroimage Clin. 2013 Aug 29;3:531-8. doi: 10.1016/j.nicl.2013.08.009	CAPS Total: 72.8(12.9)	24371791	Unspecified valence during passive scene viewing	Unclassified	Unclassified	Pictures vs baseline: PTSD Patients < Healthy controls
Nakao	2011	fMRI of patients with social anxiety disorder during a social situation task.	Neurosci Res. 2011 Jan;69(1):67-72. doi: 10.1016/j.neures.2010.09.008	STAI State: 53.2(13.3)	20888872	Self-referential processing task	Social Process	Perception and Understanding of Self (Self)	Task trials vs control trials: SAD Patients < Healthy controls
Patel	2016	Preferential recruitment of the basolateral amygdala during memory encoding of negative scenesin posttraumatic stress disorder.	Neurobiol Learn Mem. 2016 Apr;130:170-6. doi: 10.1016/j.nlm.2016.02.003	CAPS: 70.09(14.24)	26876926	Unspecified valence during passive scene viewing	Unclassified	Unclassified	Positive vs negative: PTSD Patients > Healthy controls; PTSD Patients < Healthy controls
Phan	2006	Association between amygdala hyperactivity to harsh faces and severity of social anxiety in generalized social phobia.	Biol Psychiatry. 2006 Mar 1;59(5):424-9.DOI: 10.1016/j.biopsych.2005.08.012	STAI State: 40.2(10.4)	16256956	Unspecified valence during facial affect processing	Social Process	Social Communication (Mixed)	Harsh vs happy faces: GSP Patients > Healthy controls
Prater	2013	Aberrant amygdala-frontal cortex connectivity during perception of fearful faces and at rest in generalized social anxiety disorder.	Depress Anxiety. 2013 Mar;30(3):234-41. doi: 10.1002/da.22014	LSAS: 79.35(15.41)	23184639	Unspecified valence during facial affect processing	Social Process	Social Communication	Fearful vs happy faces: SAD Patients > Healthy controls

© 2019 Janiri D et al. *JAMA Psychiatry.*

eTable 5: Post-Traumatic Stress Disorder and Anxiety Disorders: Studies Included									
**First Author**	**Year**	**Title**	**Publication**	**Patients' Symptom Severity**	**PMID**	**Task**	**RDoC Domain**	**RDoC Construct**	**Contrasts Used**
								(Mixed)	
Pujol	2013	Neural response to the observable self in social anxiety disorder.	Psychol Med. 2013 Apr;43(4):721-31. doi: 10.1017/S0033291712001857	STAI State: 30.8(8.7)	22895096	Self-referential processing task	Social Process	Perception and Understanding of Self (Self)	Self vs other condition: SAD Patients > Healthy controls; SAD Patients < Healthy controls
Sailer	2008	Altered reward processing in the nucleus accumbens and mesial prefrontal cortex of patientswith posttraumatic stress disorder.	Neuropsychologia. 2008 Sep;46(11):2836-44. doi: 10.1016/j.neuropsychologia.2008.05.022	Mean Global Severity Index: 65.5(13.9)	18597797	Reversal learning task	Positive Valence	Initial Responsiveness to Reward Attainment	Response to gain in early and late phase, gains vs losses: PTSD Patients > Healthy controls; PTSD Patients < Healthy controls
Sakamoto	2005	Parahippocampal activation evoked by masked traumatic images in posttraumatic stressdisorder: a functional MRI study.	Neuroimage. 2005 Jul 1;26(3):813-21. DOI: 10.1016/j.neuroimage.2005.02.032	CAPS: 73.8(19.2)	15955491	Negative valence during passive scene viewing	Negative Valence	Potential Threat ("Anxiety")	Masked traumatic stimulation vs control stimulation: PTSD Patients > Healthy controls; PTSD Patients < Healthy controls
Schechter	2012	An fMRI study of the brain responses of traumatized mothers to viewing their toddlers during separation and play.	Soc Cogn Affect Neurosci. 2012 Nov;7(8):969-79. doi: 10.1093/scan/nsr069	CAPS: 88.5(15.6)	22021653	Unspecified valence during facial affect processing	Social Process	Social Communication (Mixed)	Interaction of familiarity by condition: PTSD Patients > Healthy controls
Schienle	2005	Brain activation of spider phobics towards disorder-relevant, generally disgust and fear inducing pictures.	Neurosci Lett. 2005 Nov 4;388(1):1-6. DOI: 10.1016/j.neulet.2005.06.025	SPQ: 20(2.8)	16046064	Symptom Provocation Paradigm	Negative Valence	Acute Threat ("Fear")	Phobia vs neutral: Phobic Patients > Healthy controls; Phobic Patients < Healthy controls
Schienle	2007	Symptom provocation and reduction in patients suffering from spider phobia: an fMRI study on exposure therapy.	Eur Arch Psychiatry Clin Neurosci. 2007 Dec;257(8):486-93.DOI: 10.1007/s00406-007-0754-y	LSAS: 81.4(15.6)	17902000	Symptom Provocation Paradigm	Negative Valence	Acute Threat ("Fear")	First session, Phobia vs neutral: Phobic Patients < Healthy controls
Schneier	2011	Neural response to eye contact and paroxetine treatment in generalized social anxiety disorder.	Psychiatry Res. 2011 Dec 30;194(3):271-278. doi: 10.1016/j.pscychresns.2011.08.006	CAPS: 62(25.2)	22047726	Unspecified valence during facial affect processing	Social Process	Social Communication (Mixed)	Pre-treatment, Direct vs averted gaze: GSAD Patients > Healthy controls
Shin	2005	A functional magnetic resonance imaging study of amygdala and medial prefrontal cortexresponses to overtly presented fearful faces in posttraumatic stress disorder.	Arch Gen Psychiatry. 2005 Mar;62(3):273-81.	CAPS: 68.5(26.3)	15753240	Unspecified valence during facial affect processing	Social Process	Social Communication (Mixed)	Fearful vs happy: PTSD Patients > Healthy controls; PTSD Patients < Healthy controls

© 2019 Janiri D et al. *JAMA Psychiatry.*

eTable 5: Post-Traumatic Stress Disorder and Anxiety Disorders: Studies Included									
**First Author**	**Year**	**Title**	**Publication**	**Patients' Symptom Severity**	**PMID**	**Task**	**RDoC Domain**	**RDoC Construct**	**Contrasts Used**
Simmons	2008	Functional activation and neural networks in women with posttraumatic stress disorder related to intimate partner violence.	Biol Psychiatry. 2008 Oct 15;64(8):681-90. doi: 10.1016/j.biopsych.2008.05.027	SPQ: 20(2.8)	18597797	Unspecified valence during passive scene viewing	Unclassified	Unclassified	Anticipation of negative images vs anticipation of positive images: IPV-PTSD Patients > Healthy controls
Simmons	2009	Initial evidence of a failure to activate right anterior insula during affective set shifting in posttraumatic stress disorder.	Psychosom Med. 2009 May;71(4):373-7. doi: 10.1097/PSY.0b013e3181a56ed8	N.A.	19398499	Unspecified valence during passive scene viewing	Unclassified	Unclassified	Interoceptive Set Shifting: PTSD Patients < Healthy controls
Simmons	2011	Altered amygdala activation during face processing in Iraqi and Afghanistani war veterans.	Biol Mood Anxiety Disord. 2011 Oct 12;1(1):6. doi: 10.1186/2045-5380-1-6	CAPS: 69.8(13.2)	22738183	Unspecified valence during facial affect processing	Social Process	Social Communication (Mixed)	Faces vs shapes: PTSD Patients > Healthy controlss; PTSD Patients < Healthy controls
Simmons	2012	The effects of temporal unpredictability in anticipation of negative events in combat veterans with PTSD.	J Affect Disord. 2013 Apr 25;146(3):426-32. doi: 10.1016/j.jad.2012.08.006	N.A.	22910447	Unspecified valence during passive scene viewing	Unclassified	Unclassified	Predictability and valence interaction: PTSD Patients > Healthy controls; PTSD Patients < Healthy controls
Steuwe	2014	Effect of direct eye contact in PTSD related to interpersonal trauma: an fMRI study of activation of an innate alarm system.	Soc Cogn Affect Neurosci. 2014 Jan;9(1):88-97. doi: 10.1093/scan/nss105	CAPS: 71.5(15.63)	22977200	Unspecified valence during facial affect processing	Social Process	Social Communication (Mixed)	Main effect of direct gaze congruent and incongruent with body posture: PTSD Patients > Healthy controls; PTSD Patients < Healthy controls
Straube	2006a	Effects of cognitive-behavioral therapy on brain activation in specific phobia.	Neuroimage. 2006 Jan 1;29(1):125-35. DOI: 10.1016/j.neuroimage.2005.07.007	SPQ: 22.33(2.84)	16087353	Symptom Provocation Paradigm	Negative Valence	Acute Threat ("Fear")	First scanning session, spider vs mushroom: Phobic Patients > Healthy controls; Phobic Patients < Healthy controls
Straube	2006b	Neural mechanisms of automatic and direct processing of phobogenic stimuli in specific phobia.	Biol Psychiatry. 2006 Jan 15;59(2):162-70. DOI: 10.1016/j.biopsych.2005.06.013	SPQ: 22.2(2.7)	16139812	Symptom Provocation Paradigm	Negative Valence	Acute Threat ("Fear")	Identification task, spider vs mushroom: Phobic Patients > Healthy controls
van Rooij	2014	Neural correlates of trauma-unrelated emotional processing in war veterans with PTSD.	Psychol Med. 2015 Feb;45(3):575-87. doi: 10.1017/S0033291714001706	CAPS Total: 67.21(11.46)	25036523	Negative valence during passive scene viewing	Negative Valence	Potential Threat ("Anxiety")	Negative vs neutral: PTSD patients > Healthy controls
Williams	2006	Trauma modulates amygdala and medial prefrontal responses to consciously attended fear.	Neuroimage. 2006 Jan 15;29(2):347-57. DOI: 10.1016/j.neuroimage.2005.03.047	CAPS Total: 82.5(21.4)	16216534	Negative valence during facial affect processing	Negative Valence	Potential Threat ("Anxiety")	Fear vs Neutral: PTSD Patients > Healthy controls; PTSD Patients < Healthy controls
Yoon	2007	Amygdala reactivity to emotional faces at high and low intensity in generalized social phobia: a 4-Tesla functional MRI study.	Psychiatry Res. 2007 Jan 15;154(1):93-8. doi: 10.1016/j.pscychresns.2006.05.004	STAI State: 40.27(10.36)	17097275	Unspecified valence during facial affect processing	Social Process	Social Communication	Emotional faces vs baseline: GSP Patients > Healthy controls

© 2019 Janiri D et al. *JAMA Psychiatry.*

First Author	Year	Title	Publication	Patients' Symptom Severity	PMID	Task	RDoC Domain	RDoC Construct	Contrasts Used
eTable 5: Post-Traumatic Stress Disorder and Anxiety Disorders: Studies Included									
								(Mixed)	
**Studies using experiments mapping the RDoC cognitive systems domain**									
Auepperle	2016	Intimate Partner Violence PTSD and Neural Correlates of Inhibition.	J Trauma Stress. 2016 Feb;29(1):33-40. doi: 10.1002/jts.22068	N.A.	26748991	Stop task	Cognitive System	Cognitive control	Stop vs No stop: PTSD Patients > Healthy controls; PTSD Patients < Healthy controls
Bryant	2005	Neural networks of information processing in posttraumatic stress disorder: a functional magnetic resonance imaging study.	Biol Psychiatry. 2005 Jul 15;58(2):111-8. doi: 10.1016/j.biopsych.2005.03.021	N.A.	16038681	Auditory oddball	Cognitive System	Attention	Targets vs standard tones: PTSD Patients > Healthy controls; PTSD Patients < Healthy controls
Chen	2009	Insular cortex involvement in declarative memory deficits in patients with post-traumatic stressdisorder.	BMC Psychiatry. 2009 Jun 18;9:39. doi: 10.1186/1471-244X-9-39.	Distress Event Questionnaire: 43.12(5.61)	19538748	Declarative memory encoding/retrieval task	Cognitive System	Declarative Memory	Encoding and retrieval: PTSD Patients < Healthy controls
Falconer	2008	The neural networks of inhibitory control in posttraumatic stress disorder.	J Psychiatry Neurosci. 2008 Sep;33(5):413-22.	CAPS: 76.1(17.4)	18787658	Go/no-go task	Cognitive System	Cognitive control	NoGo vs Go Tasks: PTSD Patients > Healthy controls; PTSD Patients < Healthy controls
Felmingham	2009	Anterior cingulate activity to salient stimuli is modulated by autonomic arousal in posttraumatic stress disorder.	Psychiatry Res. 2009 Jul 15;173(1):59-62. doi: 10.1016/j.pscychresns.2008.12.005.	DASS: 11.5(5.4)	19446442	Auditory oddball	Cognitive System	Attention	Targets vs standard tones: PTSD Patients > Healthy controls
Henning-Fast	2009	After facing traumatic stress: Brain activation, cognition and stress coping in policemen	Journal of Psychiatric Research 43 (2009) 1146–1155. doi: 10.1016/j.jpsychires.2009.03.001	BAI: 2.8(2.74)	19358996	Paired associates learning task	Cognitive System	Declarative Memory	Encoding and retrieval: PTSD Patients > Healthy controls; PTSD Patients < Healthy controls
Jovanovic	2013	Reduced neural activation during an inhibition task is associated with impaired fear inhibition in a traumatized civilian sample.	Cortex. 2013 Jul-Aug;49(7):1884-91. doi: 10.1016/j.cortex.2012.08.011.	STAI State: 39.9(2)	23020899	Go/no-go task	Cognitive System	Cognitive control	NoGo vs Go: PTSD Patients < Healthy controls
Sareen	2007	Striatal function in generalized social phobia: a functional magnetic resonance imaging study.	Biol Psychiatry. 2007 Feb 1;61(3):396-404. DOI: 10.1016/j.biopsych.2006.05.043	STAI State: 44.2(11.9)	17097072	Implicit learning task	Cognitive System	Unclassified	Implicit learning vs random: GSP Patients < Healthy controls
van den He	2011	Common limbic and frontal-striatal disturbances in patients with obsessive compulsive disorder, panic disorder and hypochondriasis.	Psychol Med. 2011 Nov;41(11):2399-410. doi:	N.A.	21557892	Tower of London	Cognitive	Cognitive control	Planning vs baseline: Patients with panic disorder < Healthy controls

© 2019 Janiri D et al. *JAMA Psychiatry.*

eTable 5: Post-Traumatic Stress Disorder and Anxiety Disorders: Studies Included									
**First Author**	**Year**	**Title**	**Publication**	**Patients' Symptom Severity**	**PMID**	**Task**	**RDoC Domain**	**RDoC Construct**	**Contrasts Used**
uvel			10.1017/S0033291711000535.				System		
Werner	2009	Hippocampal function during associative learning in patients with posttraumatic stress disorder.	J Psychiatr Res. 2009 Jan;43(3):309-18. doi: 10.1016/j.jpsychires.2008.03.011.	CAPS: 57.75(22.06)	18490028	Paired associates learning task	Cognitive System	Declarative Memory	Encoding and retrieval: PTSD Patients > Healthy controls; PTSD Patients < Healthy controls
**Studies including cross-domain experiments**									
Ball	2013	Prefrontal dysfunction during emotion regulation in generalized anxiety and panic disorders.	Psychol Med. 2013 Jul;43(7):1475-86. doi: 10.1017/S0033291712002383. DOI: 10.1017/S0033291712002383	ASI total: 28.1(11.3)	23111120	Emotion regulation task	Cognitive-affective interaction	Cognitive-affective interaction	Maintain vs Baseline, Reappraise vs Baseline: PD and GAD Patients < Healthy controls
Chechko	2009	Unstable prefrontal response to emotional conflict and activation of lower limbic structures and brainstem in remitted panic disorder.	PLoS One. 2009 May 20;4(5):e5537. doi: 10.1371/journal.pone.0005537.	STAI, state anxiety subscale: 37.2(6.3)	19462002	Emotional stroop task	Cognitive-affective interaction	Cognitive-affective interaction	Incongruent vs congruent conditions: Patients > Healthy controls; Patients < Healthy controls
Dresler	2012	Neural correlates of the emotional Stroop task in panic disorder patients: an event-related fMRI study.	J Psychiatr Res. 2012 Dec;46(12):1627-34. doi: 10.1016/j.jpsychires.2012.09.004.	STAI, state anxiety subscale: 46.45(9.97)	23058446	Emotional stroop task	Cognitive-affective interaction	Cognitive-affective interaction	Panic-related words vs neutral words:Patients > Healthy controls
Goldin (dic)	2009a	Neural mechanisms of cognitive reappraisal of negative self-beliefs in social anxiety disorder.	Biol Psychiatry. 2009 Dec 15;66(12):1091-9. doi: 10.1016/j.biopsych.2009.07.014.	LSAS-SR: 80.1(16.8)	19717138	Emotion regulation task	Cognitive-affective interaction	Cognitive-affective interaction	Reappraise to negative self-beliefs early vs late blood oxygen level dependent responses: SAD Patients < Healthy controls
Goldin (feb)	200	Neural bases of social anxiety disorder: emotional reactivity and cognitive regulation during social and physical threat.	Arch Gen Psychiatry. 2009 Feb;66(2):170-80. doi: 10.1001/archgenpsychiatry.2008.525.	STAI, state anxiety subscale: 38.7(11.3)	19188539	Emotion regulation task	Cognitive-affe	Cognitive-affective interaction	Regulate vs look harsh faces and regulate vs look violent scenes: SAD Patients > Healthy controls; SAD Patients < Healthy controls

© 2019 Janiri D et al. *JAMA Psychiatry.*

eTable 5: Post-Traumatic Stress Disorder and Anxiety Disorders: Studies Included									
**First Author**	**Year**	**Title**	**Publication**	**Patients' Symptom Severity**	**PMID**	**Task**	**RDoC Domain**	**RDoC Construct**	**Contrasts Used**
	9b						ctive interaction		
Landré	2012	Working memory processing of traumatic material in women with posttraumatic stress disorder.	J Psychiatry Neurosci. 2012 Feb;37(2):87-94. doi: 10.1503/jpn.100167.	CAPS: 73.4(20.4)	21971161	Emotional identity task	Cognitive-affective interaction	Cognitive-affective interaction	Trauma-related vs neutral words:PTSD Patients < Healthy controls
Moon	2015a	Functional neuroanatomy on the working memory under emotional distraction in patients with generalized anxiety disorder.	Psychiatry Clin Neurosci. 2015 Oct;69(10): 609-19. doi: 10.1111/pcn.12295	STAI, state anxiety subscale: 56(12.4)	25781332	Emotional working memory task	Cognitive-affective interaction	Cognitive-affective interaction	Anxiety-inducing distractors vs rest : GAD Patients > Healthy controls; GAD Patients < Healthy controls
Moon	2015b	Explicit verbal memory impairments associated with brain functional deficits and morphological alterations in patients with generalized anxiety disorder.	J Affect Disord. 2015 Nov 1;186:328-36. doi: 10.1016/j.jad.2015.07.038.	STAI, state anxiety subscale: 57.9(14)	26277269	Emotional verbal memory task	Cognitive-affective interaction	Cognitive-affective interaction	Anxiety-inducing words vs rest: GAD Patients > Healthy controls; GAD Patients < Healthy controls
Moon	2016	Working memory dysfunction associated with brain functional deficits and cellular metabolic changes in patients with generalized anxiety disorder.	Psychiatry Res Neuroimaging. 2016 Aug 30;254:137-44. doi: 10.1016/j.pscychresns.2016.06.013.	STAI, state anxiety subscale: 58.2(11.8)	27442922	Emotional working memory task	Cognitive-affective interaction	Cognitive-affective interaction	Emotion inducing distractors vs rest : GAD Patients > Healthy controls; GAD Patients < Healthy controls
New	2009	A functional magnetic resonance imaging study of deliberate emotion regulation in resilience and posttraumatic stress disorder.	Biol Psychiatry. 2009 Oct 1;66(7):656-64. doi: 10.1016/j.biopsych.2009.05.020.	CAPS Total: 69.1(17.6)	19589502	Emotion regulation task	Cognitive-affective interaction	Cognitive-affective interaction	Diminish vs maintain and enhance vs maintain: PTSD Patients > Healthy controls; PTSD Patients < Healthy controls

© 2019 Janiri D et al. *JAMA Psychiatry.*

eTable 5: Post-Traumatic Stress Disorder and Anxiety Disorders: Studies Included									
**First Author**	**Year**	**Title**	**Publication**	**Patients' Symptom Severity**	**PMID**	**Task**	**RDoC Domain**	**RDoC Construct**	**Contrasts Used**
Reinecke	2015	Effective emotion regulation strategies improve fMRI and ECG markers of psychopathology in panic disorder: implications for psychological treatment action.	Transl Psychiatry. 2015 Nov 3;5:e673. doi: 10.1038/tp.2015.160.	HAMA: 14.6(4.1)	26529426	Emotion regulation task	Cognitive-affective interaction	Cognitive-affective interaction	Maintain vs reappraise: Patients > Healthy controls
Thomaes	2012	Treatment effects on insular and anterior cingulate cortex activation during classic and emotionalStroop interference in child abuse-related complex post-traumatic stress disorder.	Psychol Med. 2012 Nov;42(11):2337-49. doi: 10.1017/S0033291712000499.	CAPS: 86.9(14.6)	22436595	Emotional stroop task	Cognitive-affective interaction	Cognitive-affective interaction	Pre-treatment, Incongruent vs congruent: Patients > Healthy controls
Yoon	2016	Neural activity during self-referential working memory and the underlying role of the amygdala in social anxiety disorder.	Neurosci Lett. 2016 Aug 3;627:139-47. doi: 10.1016/j.neulet.2016.05.068.	LSAS-SR: 84.8(17.1)	27260987	Emotional working memory task	Cognitive-affective interaction	Cognitive-affective interaction	Encoding and Retrieval Phase vs baseline: SAD Patients > Healthy controls; SADPatients < Healthy controls
Zhang	2013	Negative emotional distraction on neural circuits for working memory in patients with posttraumatic stress disorder.	Brain Res. 2013 Sep 19;1531:94-101. doi: 10.1016/j.brainres.2013.07.042.	CAPS: 52.33(9.44)	23911835	Emotional working memory task	Cognitive-affective interaction	Cognitive-affective interaction	Negative vs neutral distractors: PTSD Patients > Healthy controls; PTSD Patients < Healthy controls
Ziv	2013	Is there less to social anxiety than meets the eye? Behavioral and neural responses to threesocio-emotional tasks.	Biol Mood Anxiety Disord. 2013 Mar 1;3(1):5. doi: 10.1186/2045-5380-3-5.	LSAS-SR: 84.1(17.5)	23448192	Emotion regulation task	Cognitive-affective interaction	Cognitive-affective interaction	React vs Asterisks: SAD Patients > Healthy controls; SAD Patients < Healthy controls
Abbreviations: ASI: Anxiety Sensitivity Index, CAPS: Clinician-Administered PTSD Scale; DASS: Depression, Anxiety and Stress Scale; BAI: Beck Anxiety Inventory; LSSP: Liebowitz Scale for Social Phobia, LSAS-SR: Liebowitz Social Anxiety Scale–Self-Report ; HAMA: Hamilton Anxiety Rating Scale, STAI: State-Trait Anxiety Inventory; SPQ: Spider Phobia Questionnaire.									

© 2019 Janiri D et al. *JAMA Psychiatry.*

7. Database Construction

7a. Samples Considered

When articles divided their sample into subgroups (e.g., patients subdivided by trauma type exposure), we included results originating from the entire sample and not the subgroups. When a single article examined the same sample longitudinally using the same fMRI tasks we only included the results at baseline. Conversely, when the same group of participants performed different tasks (e.g., using a facial affect task and a working memory task) we included results from each task. The following were recorded from each article separately for patient and control groups: number of participants, age, sex (% male), diagnostic classification system (e.g., DSM-IV, ICD-10). The medication status of the patient groups (% receiving any psychotropic medication) which 35% for major depressive disorder, 78% for bipolar disorder, 14% for post-traumatic stress disorder and 14% for anxiety disorders.

7b. Experiments Considered

Experiments (i.e., set of coordinates) were only included if derived from whole-brain analyses. When needed, coordinates were transformed from Montreal Neurology Institute space to Talairach space using the icbm_other2tal transformation[27]. For each task, we included the coordinates from the contrast between the active vs control condition; coordinates from resting-state or association analyses (e.g., correlation with clinical features) were not included. We included coordinates from multiple contrasts originating from the same task if they corresponded to different RDoC domains/constructs. For example, the monetary incentive delay task yields coordinates for contrasts between a control condition and reward anticipation or reward receipt; the coordinates of both such contrasts would be included as they respectively map to the constructs of approach/motivation and reward attainment. Based on this criterion, 26 studies contributed more than one experiment to the dataset. For tasks with multiple levels of difficulty, we included only the coordinates of the contrast corresponding to the most difficult condition. Coordinates from contrasts between different stimuli of the same active condition were not included if a control condition was available (e.g., in facial affect processing tasks contrasting emotional and neutral facial expressions, the contrasts between different emotional facial expressions were not included).

7c. Diagnostic coding of experiments

Coordinates from experiments from articles in major depressive disorder, bipolar disorder and post-traumatic stress disorder were coded accordingly. For experiments in patients with generalized anxiety disorder, panic disorder and agoraphobia, specific and social phobias we used the single coding of "anxiety disorders"; this is because the diagnostic boundaries of these disorders are unclear and because the number of experiments for each separate anxiety disorder was below the recommended minimum of 20[28]. Post-traumatic stress disorder was coded separately since it has been placed in the new category of trauma and stressor-related disorders in DSM-5. Each experiment was further coded by the direction of change in brain activity in patients relative to healthy individuals (hypo- or hyper-activation).

7d. RDoC Coding of experiments

We used the RDoC framework to code experiments (eTables 2-5) by domain and construct. The RDoC proposes a modular organization of brain function into positive valence systems, negative valence systems, cognitive systems, social processes and arousal and regulatory systems (https://www.nimh.nih.gov/research-priorities/rdoc/constructs/rdoc-matrix.shtml). Experiments that combined mechanisms attributable to more than one domain (e.g., affective Go/NoGo) were designated cross-domain (See eMethods and eTables 2-5).

7e. Coding Experiments according to symptom severity

Patient's symptom severity wad based on their mean score of the instrument used to rate their symptoms in the primary study. When multiple instruments were used, we extracted only the mean value and standard-deviation (SD) of the instrument that was most commonly employed in all other studies. The name of the instrument and the mean (SD) psychopathology rating per study are shown in eTables 3-5. To accommodate the various instruments their rating was scored as "minimal/mild", "moderate" and "severe". For most scales it was possible to conduct this scoring based on the instrument's manual. For scales scoring guidelines and without standardized value, we use the tertile scores.

7f. Coding experiments by task type

An alternative coding of experiments was based on the type of task involved in the primary studies. The primary studies that employed versions of the following: Go/no-go tasks (n=15), Stroop Color Word Task (n=5), Sternberg tasks (n=3), n-back tasks (n=15), delayed-(non)-match-to-sample tasks (n=3), paired associates learning tasks (n=4), episodic memory encoding/retrieval tasks (n=3), semantic list learning tasks (n=1), verbal fluency tasks (n=4),

© 2019 Janiri D et al. *JAMA Psychiatry.*

visual/auditory oddball tasks (n=2), tasks involving processing of self-referential information (n=9), facial affect identification tasks (n=42), tasks involving viewing of negatively valenced facial expressions (n=35), tasks involving viewing of negatively valenced scenes (n=11), tasks involving viewing of positively valenced facial expressions (n=15), tasks involving viewing of positive valenced scenes (n=5), symptom provocation tasks (n=9), fear conditioning (n=3), monetary incentive delay tasks (n=6), probabilistic reward tasks (n=8), reversal learning tasks (n=3), emotional Stroop tasks (n=9), emotion regulation tasks (n=11), emotional oddball tasks (n=4), emotional working memory tasks (n=4), emotional verbal memory tasks (n=1), emotional go/no-go tasks (n=1) and emotional attention shifting tasks (n=1). N denotes the number of experiments across all the studies included in the dataset

8. Activation Likelihood Estimation (ALE)

The ALE algorithm tests whether the spatial distribution within the brain of the peak coordinates (foci) from the experiments included in a meta-analysis differs from a random distribution[28–30]. Foci of each experiment are modeled as centers of a 3D Gaussian distribution accounting for the uncertainty associated with each focus. The full-width-at-half-maximum of these probability distributions is based on empirical data on the between-subject and between-template variance. The between-subject variance is weighted by the size of the sample that performed the experiment, so that experiments from larger samples have a higher localizing power. The probabilities of all foci associated with each experiment are aggregated using the highest probability of each voxel at any focus reported for that experiment. This approach ensures that foci from a single experiment that are in close vicinity do not exert a cumulative influence on probability values. A modeled voxel-wise activation map is then created for each experiment. The combination of all activation maps yields voxel-wise ALE scores that describe the convergence of results at each location of the brain. To distinguish true spatial convergence across experiments from random overlap, a random effects model is used to compare ALE scores against an analytically derived null-distribution map reflecting a random spatial association between experiments. The p-value of a given voxel-wise ALE score represents the proportion of equal or higher values obtained under the null-distribution. The resulting non-parametric uncorrected voxel-wise p-values are thresholded at the cluster-forming threshold of $P<0.001$. Then the size of the clusters surviving this threshold is compared against a null-distribution of cluster-sizes derived by simulating 5000 datasets of randomly distributed foci but with otherwise identical properties (number of foci, uncertainty) as the original dataset. Family-wise error correction at $P<0.05$ is then applied to this distribution to identify cluster sizes that only exceed in 5 % of all random simulations.

8a. Rationale for excluding results from region-of-interest analyses

While region-of-interest (ROI) analyses are informative in the investigation of the neural correlates of processes that are known or are predicted to involve specific regions, including them in quantitative coordinate-based meta-analyses would almost surely bias results by inflating the contribution of the corresponding regions. This is because the fundamental assumption of the Activation Likelihood Estimation approach[28–30] is that each voxel has the same a priori chance of differentiating cases from controls (null hypothesis).

To use a simple example, if all of included studies examined only the amygdala and consequently some of them reported activation of this structure, it would be almost certain that “amygdala activation” would emerge as a significant finding against a null-hypothesis of random spatial convergence across the entire brain. However, this “finding” would reflect nothing more that the propagation of the bias of looking exclusively in the amygdala. We have demonstrated such biases in our previous work by Sprooten et al. [31] where we have compared ROI and whole-brain volume analyses across diagnoses; we found that ROIs analyses led to significant over-representation of case-control differences in some brain regions (such as the amygdala). At the same time, ROI studies led to significant under-representation of other brain regions (such as the thalamus) which are rarely included in ROIs analyses but often show case- control differences in studies using whole-brain analyses. Thus, ROI analyses were excluded as they would artificially bias results in favor of voxels within these regions by violating the fundamental ALE null hypothesis.

8b. Rationale for pooling results across diagnoses

Our main analyses pooled together results across diagnoses for two reasons. First, mood, post-traumatic stress and anxiety disorders are often comorbid and hence the pooled analyses accommodate uncertainty about the symptomatic and syndromal boundaries between them. Comorbidity is often not assessed in the primary studies and therefore it is difficult to estimate its prevalence in the samples examined and its potential contribution to the neuroimaging results. Second, pooling results across diagnoses balances power, specificity and sensitivity and allows a data-driven identification of the relative significance of each suprathreshold cluster for each diagnosis based on their contribution to the likelihood of hypo or hyperactivation in that particular cluster.

8c. Rationale for pooling results across tasks and cognitive domains/constructs

© 2019 Janiri D et al. *JAMA Psychiatry.*

The Research Domain Criteria (RDoC) framework[32] provides a principled way of classifying neurocognitive tasks according to their presumed association with recognized brain circuits. However, there is no one-to-one correspondence between tasks and brain circuits; the relationship between brain structure and function has been described both as pluripotent (one-to-many) and degenerate (many-to-one)[33,34]. Therefore, any given task engages brain regions outside those predicted by the cognitive mechanisms attributed to that particular task while a single brain area may be activated by disparate tasks that may not share cognitive component[32–34]. In this context, pooling results across tasks and estimating the contribution of each RDoC domain/construct to each suprathreshold cluster accommodates the pluripotency of the tests and offers a more realistic representation of their relevance to case-control differences.

9. Reproducibility and Ancillary Analyses

To test reproducibility of results, we reproduced the meta-analyses using the Seed-Based d Mapping software (SDM, version 5.15, http://www.sdmproject.com, formerly "Signed Differential Mapping")[34,35]. The SDM the software creates maps of the effect size of case-control differences in fMRI studies by converting the t-value of each peak to Hedges' effect size and then applying an anisotropic non-normalized Gaussian kernel so that voxels more correlated with the peak have higher effect sizes. Following this, a mean map is created by performing a voxel-wise calculation of the random effects mean of the study maps, weighted by sample size and variance of each study and between-study heterogeneity. The distribution of the resulting Z-values typically deviates from normality and thus their deviation from a null distribution (i.e., random distribution) is empirically estimated using permutation statistics (i.e., randomizations of effect sizes across voxels). All the analyses conducted here were based on 50 permutations. Statistically significant clusters of convergence are identified using voxel-wise uncorrected $P<0.005$, peak-level threshold of $Z > 1$ and cluster size > 10 voxels.

Ancillary Analyses

Using the Activation Likelihood Estimation methodology described above, we conducted the following ancillary analyses:

(a) Our main analyses did not identify any clusters of hyperactivation; however, current models of affective morbidity emphasize regions of hyperactivation in response of emotionally valenced stimuli. We therefore conducted separate meta-analyses focused only on affective experiments pooled across all diagnoses.
(b) Separate diagnosis-specific meta-analyses each restricted to studies on major depressive disorder, post-traumatic stress disorder, anxiety disorders and bipolar disorder. Details of the study samples and experiments involved as shown in Table 1 and eTable 6.

Additionally, for each suprathreshold cluster derived from the main analyses we extracted the per-voxel probability of functional change from the modelled activation maps in order to estimate

(a) The contribution of symptom severity to each cluster, and
(b) The contribution of each task type to the suprathreshold clusters

© 2019 Janiri D et al. *JAMA Psychiatry.*

eTable 6. Experiments and Samples included in the analysis on affective experiments

Diagnosis	Experiments N	Sample N	Age Mean (SD)	Sex (% Male)	Medication	Sample Size N	Age Mean (SD)	Sex (% Male)
**Patient with MDD**						**Healthy Individuals**		
	101	1090	36.18 (9.53)	42%	30%	1145	33.55 (8.82)	44%
**Patients with BD**						**Healthy Individuals**		
	38	463	36.18 (9.88)	49%	72%	509	34.50 (9.20)	49%
**Patients with PTSD**						**Healthy Individuals**		
	28	549	28.34 (6.79)	33%	8%	527	27.87 (6.70)	36%
**Patients with Anxiety**						**Healthy Individuals**		
	36	365	35.19 (9.01)	46%	10%	367	34.36 (8.54)	43%

ANX=anxiety disorders; BD=Bipolar Disorder; MDD=Major depressive disorders; PTSD=Post-traumatic stress disorder. Experiment= set of coordinates of case-control differences originating from specific task contrasts; some published articles contributed more than one experiment (details in eMethods and eTables 2-5).

© 2019 Janiri D et al. *JAMA Psychiatry.*

eResults

eFigure 3: Transdiagnostic Clusters of hypoactivation (A) and hyperactivation (B) in patients relative to healthy individuals.

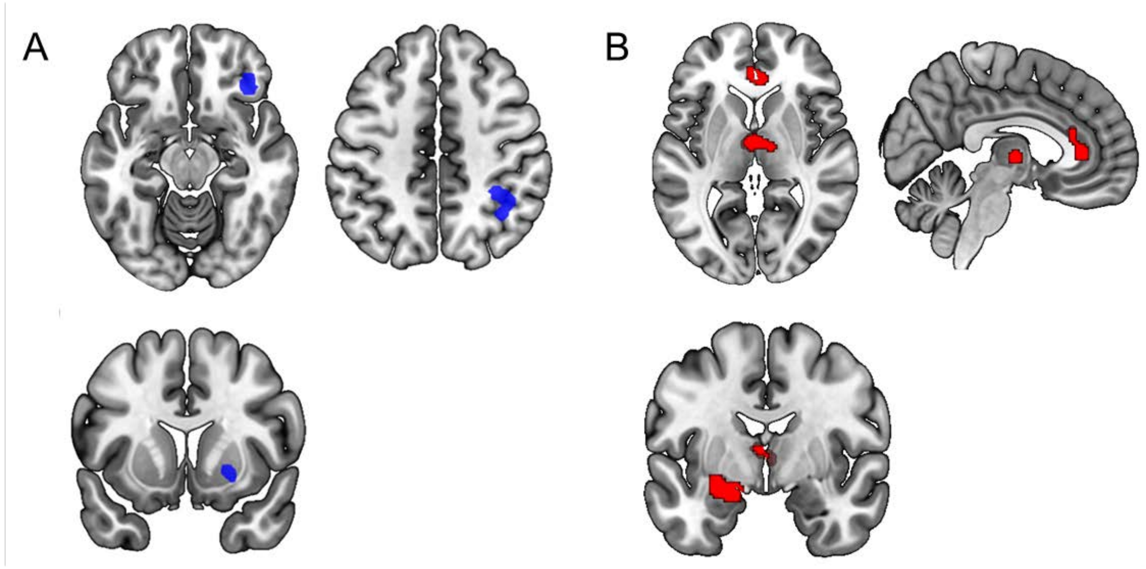

© 2019 Janiri D et al. *JAMA Psychiatry.*

2. Effect of Moderator Variables

2a. Contribution of moderators to the transdiagnostic clusters of hypoactivation

None of the moderator variables survived correction for multiple testing (Bonferroni corrected p-value per cluster was 0.008 (0.05/6). The details per cluster are as follows (uncorrected p-shown):

Right inferior prefrontal cortex/insula: age of patients (ρ=-0.05, *P*=0.78), age of healthy individuals (ρ=-0.02, *P*=0.88), percentage of male patients (ρ=-0.34, *P*=0.07), percentage of healthy male individuals (ρ=-0.27, *P*=0.16), medication status (ρ=0.13, *P*=0.51), magnetic field (χ^2=1.04, *P*=0.59).

Right inferior parietal lobule: age of patients (ρ=0.55, *P*=0.05), age of healthy individuals (ρ=0.63, *P*=0.01), percentage of male patients (ρ=0.29, *P*=0.33), percentage of healthy male individuals (ρ=0.45, *P*=0.12), medication status (ρ=0.45, *P*=0.12), magnetic field (χ^2=0, *P*=1.00).

Right putamen: age of patients (ρ=0.47, *P*=0.05), age of healthy individuals (ρ=0.43, *P*=0.07), percentage of male patients (ρ=0.01, *P*=0.95), percentage of healthy male individuals (ρ=0.04, *P*=0.86), medication status (ρ=0.36, *P*=0.16), magnetic field (χ^2=1.24, *P*=0.26).

1b. Contribution of moderators to the transdiagnostic clusters of hyperactivation

The Bonferroni corrected p-value per cluster was set at 0.008 (0.05/6). The details per cluster are as follows (uncorrected p-values shown):

Left amygdala/parahippocampal gyrus: age of patients (ρ=-0.05, *P*=0.82), age of healthy individuals (ρ=-0.33, *P*=0.14), percentage of male patients (ρ=-0.03, *P*=0.88), percentage of healthy male individuals (ρ=-0.16, p=0.51), medication status (ρ=-0.41, *P*=0.06), magnetic field (χ^2=4.64, *P*=0.09).

Left thalamus: age of patients (ρ=-0.09, *P*=0.70), age of healthy individuals (ρ=-0.10, *P*=0.67), percentage of male patients (ρ=0.40, *P*=0.07), percentage of healthy male individuals (ρ=0.37, *P*=0.10), medication status (ρ=0.06, *P*=0.79), magnetic field (χ^2=0.29, *P*=0.58).

Perigenual/Dorsal anterior cingulate cortex: age of patients (ρ=-0.32, *P*=0.23), age of healthy individuals (ρ=-0.36, *P*=0.17), percentage of male patients (ρ=-0.68, *P*=0.004), percentage of male healthy individuals (ρ=-0.64, *P*=0.01), medication status (ρ=-0.44, *P*=0.11), magnetic field (χ^2=0.58, *P*=0.74). Experiments originating from samples with higher percentage of male patients made a significant contribution to this cluster.

3. Contribution of each Research Domain Criterion construct to each transdiagnostic suprathreshold cluster

The contribution of different constructs showed numerical but not statistical differences within each cluster as detailed in eTable 7.

4. Contribution of symptom severity to each transdiagnostic suprathreshold cluster

The contribution of symptom severity did not reach statistical significance for any transdiagnostic cluster of hyperactivation (amygdala/parahippocampal gyrus *P*=0.25; thalamus *P*=0.49; perigenual/dorsal anterior cingulate *P*=0.23) or hypoactivation (inf. prefrontal cortex/insula *P*=0.06; inf. parietal lobule *P*=0.37; putamen *P*=0.07)

5. Contribution of task type to each transdiagnostic suprathreshold cluster

The contribution of the different tasks to each cluster is shown in eTable 8. We calculated the contribution of type of task in the per-voxel probability of functional change from the modelled activation maps using non-parametric Kruskal–Wallis tests. This effect did not reach statistical significance of any transdiagnostic cluster of hypoactivation (inf. prefrontal cortex/insula χ^2=13.31, *P*=0.42; inf. parietal lobule χ^2=10.14, *P*=0.18; putamen χ^2=13.90, *P*=0.15) or hyperactivation (amygdala/parahippocampal gyrus χ^2=12.78 *P*=0.46; thalamus χ^2=12.57 *P*=0.32; perigenual/dorsal anterior cingulate χ^2=11.50 *P*=0.24)

© 2019 Janiri D et al. *JAMA Psychiatry.*

eTable 7. Percentage Contribution of RDoC Construct to each transdiagnostic suprathreshold cluster						
	Clusters of relative hypoactivation in patients			Clusters of relative hyperactivation in patients		
**RDoC Construct (Percentage Contribution per Cluster)**	**Inferior Prefrontal Cortex/Insula x=40, y=30, z=-10**	**Inferior Parietal Lobule x=38, y=-48, z=46**	**Putamen x=24, y=9, z= -6**	**Amygdala/ Parahippocampal Gyrus x=-22, y=-2, z=-15**	**Thalamus x=-2, y=-12, z=4**	**Perigenual/Dorsal Anterior Cingulate Cortex x=0 , y=34 , z=12**
Cognitive control	24.9	35.17	24.69	4.74	-	-
Working memory	3.91	15.09	-	-	0.84	27.54
Episodic Memory	3.57	5.44	4.73	-	-	-
Language	1.85	-	-	-	0.34	-
Attention	-	-	-	-	7.92	-
Social communication	10.4	14.6	23.17	21.83	1.56	0.25
Perception of Self	5.83	-	-	-	-	-
Potential threat	13.3	12.8	3.93	19.56	31.96	23.19
Acute threat	-	0.35	-	27.13	21.87	26.08
Reward Attainment	12.35	-	5.03	-	14.12	-
Approach Motivation	8.34	-	12.51	1.01	6.6	7.03
Frustrative Non-reward	-	-	3.38	5.07	6.31	-
**Cross-Domain**	15.55	16.55	22.56	20.66	8.48	15.91

RDoC=Research Domain Criteria; peak coordinates in Montreal Neurological Institute space: x, y, z=sagittal, coronal, axial

© 2019 Janiri D et al. *JAMA Psychiatry.*

eTable 8. Percentage Contribution of tasks to each transdiagnostic suprathreshold cluster						
	**Clusters of relative hypoactivation in patients**			**Clusters of relative hyperactivation in patients**		
**Task Type (Percentage Contribution per Cluster)**	**Inf. Prefrontal Cortex/Insula<br><br>x=40, y=30, z=-10**	**Inferior Parietal Lobule<br><br>x=38, y=-48, z=46**	**Putamen<br><br>x=24, y=9, z= -6**	**Amygdala/ Parahippocampal Gyrus<br>x=-22, y=-2, z=-15**	**Thalamus<br><br>x=-2, y=-12, z=4**	**Perigenual/Dorsal Anterior Cingulate Cortex<br>x=0 , y=34 , z=12**
Go/no-go tasks	10.74	2.28	24.69	4.74	-	-
Stroop tasks	14.17	32.89	-	-	-	-
Sternberg tasks	3.91	-	-	-	-	-
N-back tasks	-	-	-	-	0.84	19.02
Delayed-(non)-match-to-sample tasks	-	15.09	-	-	-	8.52
Paired associates learning tasks	3.25	0.05	4.62	-	0.34	-
Episodic memory encoding/retrieval tasks	0.13	5.39	0.11	-	-	-
Semantic list learning tasks	0.19	-	-	-	-	-
Verbal fluency tasks	1.85	-	-	-	-	-
Visual/Auditory oddball tasks	-	-	-	-	7.92	-
Tasks involving processing of self-referential information	5.83	-	-	-	-	-
Facial affect identification tasks	10.4	14.6	23.17	21.83	1.56	0.25
Tasks involving viewing of negatively	13.3	12.8	3.93	19.56	21.62	15.29

© 2019 Janiri D et al. *JAMA Psychiatry.*

eTable 8. Percentage Contribution of tasks to each transdiagnostic suprathreshold cluster						
	**Clusters of relative hypoactivation in patients**			**Clusters of relative hyperactivation in patients**		
**Task Type (Percentage Contribution per Cluster)**	**Inf. Prefrontal Cortex/Insula**<br><br>**x=40, y=30, z=-10**	**Inferior Parietal Lobule**<br><br>**x=38, y=-48, z=46**	**Putamen**<br><br>**x=24, y=9, z= -6**	**Amygdala/ Parahippocampal Gyrus**<br>**x=-22, y=-2, z=-15**	**Thalamus**<br><br>**x=-2, y=-12, z=4**	**Perigenual/Dorsal Anterior Cingulate Cortex**<br>**x=0 , y=34 , z=12**
valenced facial expressions						
Tasks involving viewing of negatively valenced scene	-	-	-	-	10.34	1.02
Tasks involving viewing of positively valenced facial expressions	-	-	12.46	-	6.60	0.03
Tasks involving viewing of positive and negatively valenced scenes	6.03	-	3.13	10.98	-	-
Symptom provocation tasks	-	0.35	-	21.92	9.28	26.08
Fear conditioning	-	-	-	5.21	12.59	-
Monetary incentive delay tasks	20.65	-	0.07	1.01	14.12	13.87
Probabilistic reward tasks	0.04	-	3.89	5.07	6.31	-
Reversal learning tasks	-	-	4.50	-	-	-
Emotional Stroop tasks	2.42	-	-	-	-	-
Emotional regulation tasks	3.80	0.33	0.01	0.73	8.38	-
Emotional oddball tasks	0.03	-	-	-	-	6.61
Emotional working memory tasks	3.26	-	-	-	-	-

© 2019 Janiri D et al. *JAMA Psychiatry.*

eTable 8. Percentage Contribution of tasks to each transdiagnostic suprathreshold cluster

	Clusters of relative hypoactivation in patients			Clusters of relative hyperactivation in patients		
**Task Type (Percentage Contribution per Cluster)**	**Inf. Prefrontal Cortex/Insula x=40, y=30, z=-10**	**Inferior Parietal Lobule x=38, y=-48, z=46**	**Putamen x=24, y=9, z= -6**	**Amygdala/ Parahippocampal Gyrus x=-22, y=-2, z=-15**	**Thalamus x=-2, y=-12, z=4**	**Perigenual/Dorsal Anterior Cingulate Cortex x=0 , y=34 , z=12**
Emotional verbal memory tasks	-	-	0.42	8.95	-	-
Emotional Go/NoGo tasks	-	-	-	-	0.05	0.3
Emotional attention shifting tasks	-	-	-	-	0.05	-
Emotional Stroop tasks	-	16.22	19	-	-	9.01

peak coordinates in Montreal Neurological Institute space: x, y, z=sagittal, coronal, axial

© 2019 Janiri D et al. *JAMA Psychiatry.*

6. Reproducibility

The SDM analyses largely confirmed the results of the ALE analyses. eFigure 3 illustrates this in connection to the transdiagnostic clusters of hypoactivation.

eFigure 4: Results obtained using Activation Likelihood Estimation (ALE) and Seed-Based *d* Mapping (SDM).

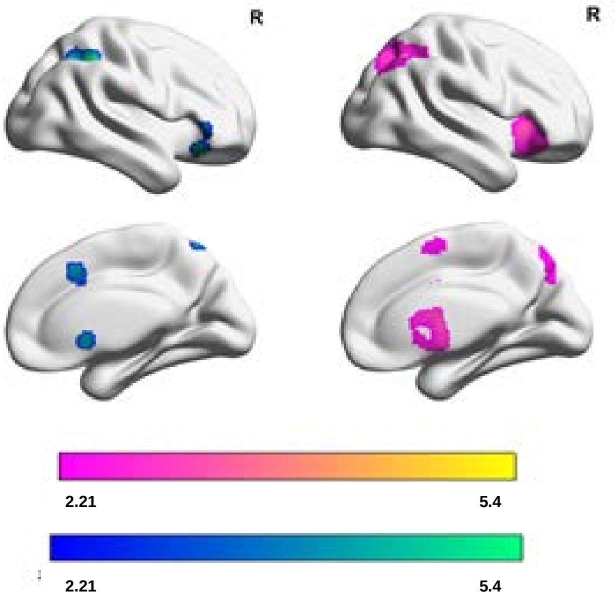

Color bars indicate z-values for the probability of patients having less activation than healthy individuals (ALE: blue-green bar; SDM: pale red-yellow bar); the left panel shows the ALE clusters (in blue-green) and the right panel shows the SDM clusters (in pale red-yellow).

© 2019 Janiri D et al. *JAMA Psychiatry.*

7. Ancillary Analyses

7a. Pooling data across diagnoses for affective experiments

Coordinates of hypo- and hyperactivation in patients relative to healthy individuals arising from all experiments involving affective and social cognition processing (i.e., those experiments listed in eTable 2 under the domains of positive valence, negative valence and social processes) were considered in two separate meta-analyses across all disorders (eTable 6). Statistically significant clusters were identified using a cluster-level familywise-error-corrected threshold of $P<0.05$ (cluster-forming threshold at voxel-level $P<0.001$). No cluster of hyperactivation in patients relative to healthy individual was detected. Conversely, patients across all disorders showed decreased likelihood of activation in a single cluster centered in the right Inferior frontal gyrus (peak MNI coordinates: x=40, y=30, z=-14; 904 mm^3) confirming that this cluster is pertinent to case-control differences during affective processing.

7b. Diagnosis-specific meta-analysis

In all ancillary analyses we used a cluster-level familywise-error-corrected threshold of $P<0.05$ (cluster-forming threshold at voxel-level $P<0.001$). We did not identify any additional disorder-specific clusters of hypo- or hyperactivation in diagnosis-specific meta-analyses of major depressive disorder, post-traumatic stress disorder and anxiety disorders. This remained the case for a sub-analysis restricted only to patients with major depressive disorder that were in a depressive episode at the time of scanning (135 experiments) or were unmedicated at the time of scanning (75 experiments). No additional cluster were identified in a separate sub-analyses restricted only to remitted bipolar patients [number of experiments: 64; sample: 947 patients, 1042 healthy individuals; age: patients 38.13 years (SD: 10.26). healthy individuals 36.70 years (SD: 10.22); sex: 44% of male patients, 45% of male healthy individuals; medication: 82% of patients were medicated]. We did not conduct sub-analysis for patients in manic/hypomanic or depressive states as the experiments available were below the recommended minimum of 20 (hypomanic/manic=14, depressed=16).

© 2019 Janiri D et al. *JAMA Psychiatry.*

Online References

1. Fitzgerald PB, Laird AR, Maller J, Daskalakis ZJ. A meta-analytic study of changes in brain activation in depression. *Hum Brain Mapp*. 2008;29(6):683-695. doi:10.1002/hbm.20426
2. Hamilton JP, Etkin A, Furman DJ, Lemus MG, Johnson RF, Gotlib IH. Functional Neuroimaging of Major Depressive Disorder: A Meta-Analysis and New Integration of Baseline Activation and Neural Response Data. *Am J Psychiatry*. 2012;169(7):693-703. doi:10.1176/appi.ajp.2012.11071105
3. Delvecchio G, Fossati P, Boyer P, et al. Common and distinct neural correlates of emotional processing in Bipolar Disorder and Major Depressive Disorder: A voxel-based meta-analysis of functional magnetic resonance imaging studies. *Eur Neuropsychopharmacol*. 2012;22(2):100-113. doi:10.1016/j.euroneuro.2011.07.003
4. Diener C, Kuehner C, Brusniak W, Ubl B, Wessa M, Flor H. A meta-analysis of neurofunctional imaging studies of emotion and cognition in major depression. *Neuroimage*. 2012;61(3):677-685. doi:10.1016/j.neuroimage.2012.04.005
5. Graham J, Salimi-Khorshidi G, Hagan C, et al. Meta-analytic evidence for neuroimaging models of depression: state or trait? *J Affect Disord*. 2013;151(2):423-431. doi:10.1016/j.jad.2013.07.002
6. Zhang WN, Chang SH, Guo LY, Zhang KL, Wang J. The neural correlates of reward-related processing in major depressive disorder: A meta-analysis of functional magnetic resonance imaging studies. *J Affect Disord*. 2013;151(2):531-539. doi:10.1016/j.jad.2013.06.039
7. Lai C-H. Patterns of Cortico-Limbic Activations During Visual Processing of Sad Faces in Depression Patients: A Coordinate-Based Meta-Analysis. *J Neuropsychiatry Clin Neurosci*. 2014;26(1):34-43. doi:10.1176/appi.neuropsych.12060143
8. Palmer SM, Crewther SG, Carey LM. A Meta-Analysis of Changes in Brain Activity in Clinical Depression. *Front Hum Neurosci*. 2015;8(January):1-19. doi:10.3389/fnhum.2014.01045
9. Wang X-L, Du M-Y, Chen T-L, et al. Neural correlates during working memory processing in major depressive disorder. *Prog Neuropsychopharmacol Biol Psychiatry*. 2015;56:101-108. doi:10.1016/j.pnpbp.2014.08.011
10. Müller VI, Cieslik EC, Serbanescu I, Laird AR, Fox PT, Eickhoff SB. Altered Brain Activity in Unipolar Depression Revisited. *JAMA Psychiatry*. 2017;74(1):47. doi:10.1001/jamapsychiatry.2016.2783
11. Keren H, O'Callaghan G, Vidal-Ribas P, et al. Reward Processing in Depression: A Conceptual and Meta-Analytic Review Across fMRI and EEG Studies. *Am J Psychiatry*. 2018;(19):appi.ajp.2018.1. doi:10.1176/appi.ajp.2018.17101124
12. Chen CH, Suckling J, Lennox BR, Ooi C, Bullmore ET. A quantitative meta-analysis of fMRI studies in bipolar disorder. *Bipolar Disord*. 2011;13(1):1-15. doi:10.1111/j.1399-5618.2011.00893.x
13. Houenou J, Frommberger J, Carde S, et al. Neuroimaging-based markers of bipolar disorder: Evidence from two meta-analyses. *J Affect Disord*. 2011;132(3):344-355. doi:10.1016/j.jad.2011.03.016
14. Delvecchio G, Sugranyes G, Frangou S. Evidence of diagnostic specificity in the neural correlates of facial affect processing in bipolar disorder and schizophrenia: A meta-analysis of functional imaging studies.

© 2019 Janiri D et al. *JAMA Psychiatry.*

Psychol Med. 2013;43(3):553-569. doi:10.1017/S0033291712001432

15. Hajek T, Alda M, Hajek E, Ivanoff J. Functional neuroanatomy of response inhibition in bipolar disorders - Combined voxel based and cognitive performance meta-analysis. *J Psychiatr Res*. 2013;47(12):1955-1966. doi:10.1016/j.jpsychires.2013.08.015
16. Alústiza I, Radua J, Pla M, Martin R, Ortuño F. Meta-analysis of functional magnetic resonance imaging studies of timing and cognitive control in schizophrenia and bipolar disorder: Evidence of a primary time deficit. *Schizophr Res*. 2017;188:21-32. doi:10.1016/j.schres.2017.01.039
17. Etkin A, Wager TD. Functional Neuroimaging of Anxiety: A Meta-Analysis of Emotional Processing in PTSD, Social Anxiety Disorder, and Specific Phobia. *Am J Psychiatry*. 2007;164(10):1476-1488. doi:10.1176/appi.ajp.2007.07030504
18. Hayes JP, Hayes SM, Mikedis AM, et al. Quantitative meta-analysis of neural activity in posttraumatic stress disorder. *Biol Mood Anxiety Disord*. 2012;2(1):9. doi:10.1186/2045-5380-2-9
19. Hattingh CJ, Ipser J, Tromp SA, et al. Functional magnetic resonance imaging during emotion recognition in social anxiety disorder: an activation likelihood meta-analysis. *Front Hum Neurosci*. 2013;6(January):1-7. doi:10.3389/fnhum.2012.00347
20. Ramage AE, Laird AR, Eickhoff SB, et al. A coordinate-based meta-analytic model of trauma processing in posttraumatic stress disorder. *Hum Brain Mapp*. 2013;34(12):3392-3399. doi:10.1002/hbm.22155
21. Sartory G, Cwik J, Knuppertz H, et al. In Search of the Trauma Memory: A Meta-Analysis of Functional Neuroimaging Studies of Symptom Provocation in Posttraumatic Stress Disorder (PTSD). *PLoS One*. 2013;8(3). doi:10.1371/journal.pone.0058150
22. Ipser JC, Singh L, Stein DJ. Meta-analysis of functional brain imaging in specific phobia. *Psychiatry Clin Neurosci*. 2013;67(5):311-322. doi:10.1111/pcn.12055
23. Stark EA, Parsons CE, Van Hartevelt TJ, et al. Post-traumatic stress influences the brain even in the absence of symptoms: A systematic, quantitative meta-analysis of neuroimaging studies. *Neurosci Biobehav Rev*. 2015;56:207-221. doi:10.1016/j.neubiorev.2015.07.007
24. Boccia M, D'Amico S, Bianchini F, Marano A, Giannini AM, Piccardi L. Different neural modifications underpin PTSD after different traumatic events: an fMRI meta-analytic study. *Brain Imaging Behav*. 2016;10(1):226-237. doi:10.1007/s11682-015-9387-3
25. Gentili C, Cristea IA, Angstadt M, et al. Beyond emotions: A meta-analysis of neural response within face processing system in social anxiety. *Exp Biol Med*. 2016;241(3):225-237. doi:10.1177/1535370215603514
26. Wang H-Y, Zhang X-X, Si C-P, et al. Prefrontoparietal dysfunction during emotion regulation in anxiety disorder: a meta-analysis of functional magnetic resonance imaging studies. *Neuropsychiatr Dis Treat*. 2018;Volume 14:1183-1198. doi:10.2147/NDT.S165677
27. Laird AR, Robinson JL, McMillan KM, et al. Comparison of the disparity between Talairach and MNI coordinates in functional neuroimaging data: Validation of the Lancaster transform. *Neuroimage*. 2010;51(2):677-683. doi:10.1016/j.neuroimage.2010.02.048
28. Eickhoff SB, Nichols TE, Laird AR, et al. Behavior, sensitivity, and power of activation likelihood

© 2019 Janiri D et al. *JAMA Psychiatry.*

estimation characterized by massive empirical simulation. *Neuroimage*. 2016;137:70-85. doi:10.1016/j.neuroimage.2016.04.072

29. Eickhoff S, Laird A, Grefkes C, Wang LE, Zilles K, Fox PT. Coordinate-based ALE meta-analysis of neuroimaging data: a random-effects approach based on empirical estimates of spatial uncertainty. *Hum Brain Mapp*. 2009;30(9):2907-2926. doi:10.1002/hbm.20718.

30. Eickhoff SB, Bzdok D, Laird AR, Kurth F, Fox PT. Activation likelihood estimation revisited. *Neuroimage*. 2012;59(3):2349-2361. doi:10.1016/j.neuroimage.2011.09.017.

31. Sprooten E, Rasgon A, Goodman M, et al. Addressing reverse inference in psychiatric neuroimaging: Meta-analyses of task-related brain activation in common mental disorders. Hum Brain Mapp. 2017;38(4):1846-1864. doi:10.1002/hbm.23486

32. Insel T, Cuthbert B, Garvey M, et al. Research Domain Criteria (RDoC): Toward a new classification framework for research on mental disorders. *Am J Psychiatry*. 2010;167(7):748-751. doi:10.1176/appi.ajp.2010.09091379

33. Pessoa L. Understanding Brain Networks. *Phys Life Rev*. 2015;11(3):400-435. doi:10.1016/j.plrev.2014.03.005.

34. Price CJ, Friston KJ. Functional ontologies for cognition: The systematic definition of structure and function. *Cogn Neuropsychol*. 2005;22(3):262-275. doi:10.1080/02643290442000095

35. Radua J, Mataix-Cols D, Phillips ML, et al. A new meta-analytic method for neuroimaging studies that combines reported peak coordinates and statistical parametric maps. *Eur Psychiatry*. 2012;27(8):605-611. doi:10.1016/j.eurpsy.2011.04.001

36. Radua J, Rubia K, Canales-Rodríguez EJ, Pomarol-Clotet E, Fusar-Poli P, Mataix-Cols D. Anisotropic kernels for coordinate-based meta-analyses of neuroimaging studies. *Front Psychiatry*. 2014;5(FEB):1-8. doi:10.3389/fpsyt.2014.00013

© 2019 Janiri D et al. *JAMA Psychiatry.*
